# Periodic Light Modulations for Low‐Cost Wide‐Field Imaging of Luminescence Kinetics Under Ambient Light

**DOI:** 10.1002/advs.202413291

**Published:** 2025-01-17

**Authors:** Hélène Merceron, Ian Coghill, Aliénor Lahlou, Marie‐Aude Plamont, Ludovic Jullien, Thomas Le Saux

**Affiliations:** ^1^ PASTEUR Département de chimie École normale supérieure PSL University, Sorbonne Université, CNRS 24, rue Lhomond Paris 75005 France; ^2^ IMPMC UMR 7590 Sorbonne Université, CNRS, MNHN, IRD, Museum National d'Histoire Naturelle 4 place Jussieu Paris 75005 France; ^3^ Sony Computer Science Laboratories Paris 75005 France

**Keywords:** kinetic fingerprint under ambient light, luminescence lifetime imaging, wide‐field luminescence kinetics imaging

## Abstract

Imaging luminescence kinetics is invaluable in many fields, including biology and chemistry. However, the luminescence lifetime of most photo‐activated states is in the low ns‐µs range and its measurement requires adding costly image intensifiers to cameras to access the fast phenomena present. Here, the Rectified Imaging under Optical Modulation (RIOM) and Heterodyne Imaging under Optical Modulation (HIOM) protocols make this possible with standard low‐cost cameras only, even under ambient light. RIOM and HIOM originate from a thorough theoretical analysis, which showed that modulated illumination of any reversibly photoactivable luminophore probed at high frequency generates components of the luminescence response that are detectable at low frequency: RIOM harnesses the mean luminescence response to modulated light at a single frequency, whereas HIOM exploits the luminescence response to modulated light at two close frequencies. When the luminophore behavior obeys a two‐state model, the frequency dependence of the RIOM and HIOM observables can be used to extract a photoactivation time. In this work, their ability to retrieve maps of the luminescence lifetime of a reversibly photoswitchable protein, and phosphorescent microsensors are demonstrated. Where the luminophore does not obey a two‐state model, RIOM and HIOM can still retrieve rich information, as here exemplified by the recovery of kinetic fingerprints of the physiological state of a plant.

## Graphical abstract

Imaging fast luminescence kinetics has required costly camera setups. The RIOM and HIOM protocols relying on modulated illumination are introduced to make this possible with standard low‐cost cameras, even under ambient light. These are implemented to measure the photoactivation time of a reversibly photoswitchable protein and microsensors and recover kinetic fingerprints of the physiological state of a plant.

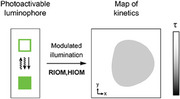

## 1 Introduction

Imaging luminescent systems has countless applications, including bioimaging,^[^ ^1^, ^2^, ^3^ ^]^ active packaging,^[^ ^4^ ^]^ encryption and anti‐counterfeiting,^[^ ^5^, ^6^, ^7^, ^8^ ^]^ mapping oxygenation and dye‐assisted surgery,^[^ ^9^, ^10^ ^]^ and process control of photoactive materials.^[^ ^11^, ^12^, ^13^, ^14^, ^15^ ^]^ In many of these, there is a strong interest in benefiting from multiplexing opportunities at low cost and possibly under adverse optical conditions such as the presence of a background of endogenous luminescence or ambient light.

Contrast information is often retrieved in the spectral domain.^[^ ^16^ ^]^ Here the number of discernable luminophores is intrinsically limited by their bandwidth of absorption and emission. Hence, 3–4 luminophores can typically be spectrally discriminated in real‐time luminescence imaging. This number can further increase to 7–9 with advanced data processing, but at the cost of a degraded photon budget and an increased computation time.^[^ ^17^, ^18^ ^]^ Furthermore, spectral discrimination necessitates extensive hardware (light sources, optics corrected for chromatic aberration, dichroic mirrors, optical filters) and demanding data processing for image extraction in the presence of interfering light.

Complementary contrast information can be retrieved from analyzing the kinetics of the luminophore photocycle. The latter involves the photo‐activation of the luminophore and its relaxation back to its initial unactivated state by photochemical and/or thermal relaxation and the associated rate constants add a discriminative kinetic dimension to the spectral domain.^[^ ^19^ ^]^ Hence, contrast against an interfering background can be achieved by tailoring the lifetime of the targeted luminophores in a range departing from the ones associated with endogenous emitters^[^ ^20^ ^]^ and fluctuations of ambient light.^[^ ^21^ ^]^ Yet, the lifetime of most photo‐activated states is in the low ns‐µs range and its measurement with a camera, normally limited to kHz/ms (frequency/time) detection, is challenging. In time‐gated imaging,^[^ ^22^, ^23^ ^]^ a pulsed light source synchronized with a time‐gated microchannel plate image intensifier coupled to a camera excites the luminophore and detects luminescence over a short time after a tunable delay. The luminescence lifetime is then recovered by varying the gate delay. In frequency domain luminescence lifetime imaging,^[^ ^24^, ^25^, ^26^, ^27^ ^]^ a continuous wave light source, modulated at high angular frequency *ω*, excites the luminophore, and the image is obtained with a specialized dual tap CMOS sensor or a camera coupled to a microchannel plate image intensifier modulated at an angular frequency near (heterodyne mode) or at (homodyne mode) *ω*. The luminescence lifetime is determined from the phase delay and modulation depth of the luminescence signal relative to that of the excitation light. Hence, at present, both types of optical setups have to be equipped with sophisticated and costly detectors endowed with high‐frequency modulation or fast gating in order to image luminescence kinetics. Here, we introduce, validate, and exploit two protocols that enable this with simple low‐cost optical setups featuring standard cameras.

## 2 Results

### 2.1 Principle

We harness that any reversibly photoactivable luminophore probed at high frequency generates components of the luminescence dynamic response that are detectable at low frequency: i) the mean luminescence response to modulated light at a single frequency and the luminescence response to constant light at the mean value of the modulated light differ;^[^ ^28^, ^29^, ^30^ ^]^ ii) the luminescence response to modulated light at two frequencies generates luminescence modulation at linear combinations of the two exciting frequencies, and at their difference in particular.^[^ ^31^ ^]^ The preceding features have already been exploited for discriminating spectrally similar luminophores.^[^ ^29^, ^30^, ^31^ ^]^

**Figure 1:**
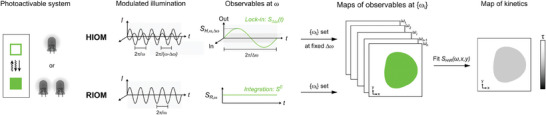
Principle of the HIOM and RIOM protocols. Modulated illumination involving one or two light sources photo‐activates a luminophore, which deactivates via a light‐driven pathway (wavy arrow) or thermal relaxation (straight arrow). In HIOM, the amplitude *S_H_* of the quadrature‐projected component of the luminescence modulated at low angular frequency *Δω* is imaged at low acquisition frequency under illumination modulated at two high angular frequencies *ω* and *ω‐Δω*. The map of luminescence lifetime *τ* is retrieved from exploiting the *S_H_* dependence on *ω*, observed at the constant difference of angular frequency *Δω*. In RIOM, the average luminescence *S_R_* is imaged at a low acquisition frequency under illumination modulated at a high angular frequency *ω*. The map of luminescence lifetime *τ* is retrieved from exploiting the dependence of *S_R_* on *ω*.on ω.

In this report, we aimed to go beyond the use of this, targeting instead the recovery of photo‐activation kinetics, even in the presence of ambient light. With this aim, we performed an in‐depth theoretical analysis with a well‐established two‐state photodynamic model (see Sections 1 and A–D in the Supporting Information).^[^ ^28^ ^]^ It led us to introduce two protocols, based on two different observables, for wide‐field luminescence kinetics imaging (**Figure** **1**): Heterodyne Imaging under Optical Modulation (HIOM) and Rectified Imaging under Optical Modulation (RIOM). In HIOM, the sample is exposed to modulated illumination at two high angular frequencies *ω* and *ω−Δω*. When this dual excitation is synchronized in antiphase, the HIOM observable is the amplitude of the quadrature‐projected luminescence component at the low *Δω* angular frequency. In RIOM, the sample is submitted to modulated illumination at one high angular frequency *ω*, and the RIOM observable is the mean luminescence.

Accessing the luminescence kinetics requires recording the HIOM or RIOM observables under informed conditions (see Sections 1 and A–D in the Supporting Information). The mean illumination intensity must be adjusted to roughly balance the rates of photo‐activation and deactivation of the luminophore, while *ω* needs to be scanned over a 2–3 orders of magnitude wide range around the inverse of the photo‐activation time. The resulting *ω*‐dependence forms, at each image pixel, a kinetic fingerprint, which can be further processed with robust fitting functions (see Sections 1 and A–D in the Supporting Information) to yield a map of the photo‐activation time, assuming the behavior approximates a two‐state photodynamic model (see Section E in the Supporting Information).

### 2.2 HIOM and RIOM Validation for Lifetime Measurements

HIOM and RIOM have been experimentally validated with luminophores exhibiting luminescence kinetics spanning a wide s‐µs range using two home‐built optical setups, an epifluorescence microscope and a fluorescence macroimager, both featuring low‐cost illumination sources, in the form of Light Emitting Diodes (LEDs), and cameras with low‐frequency acquisition.^[^ ^32^ ^]^

**Figure 2:**
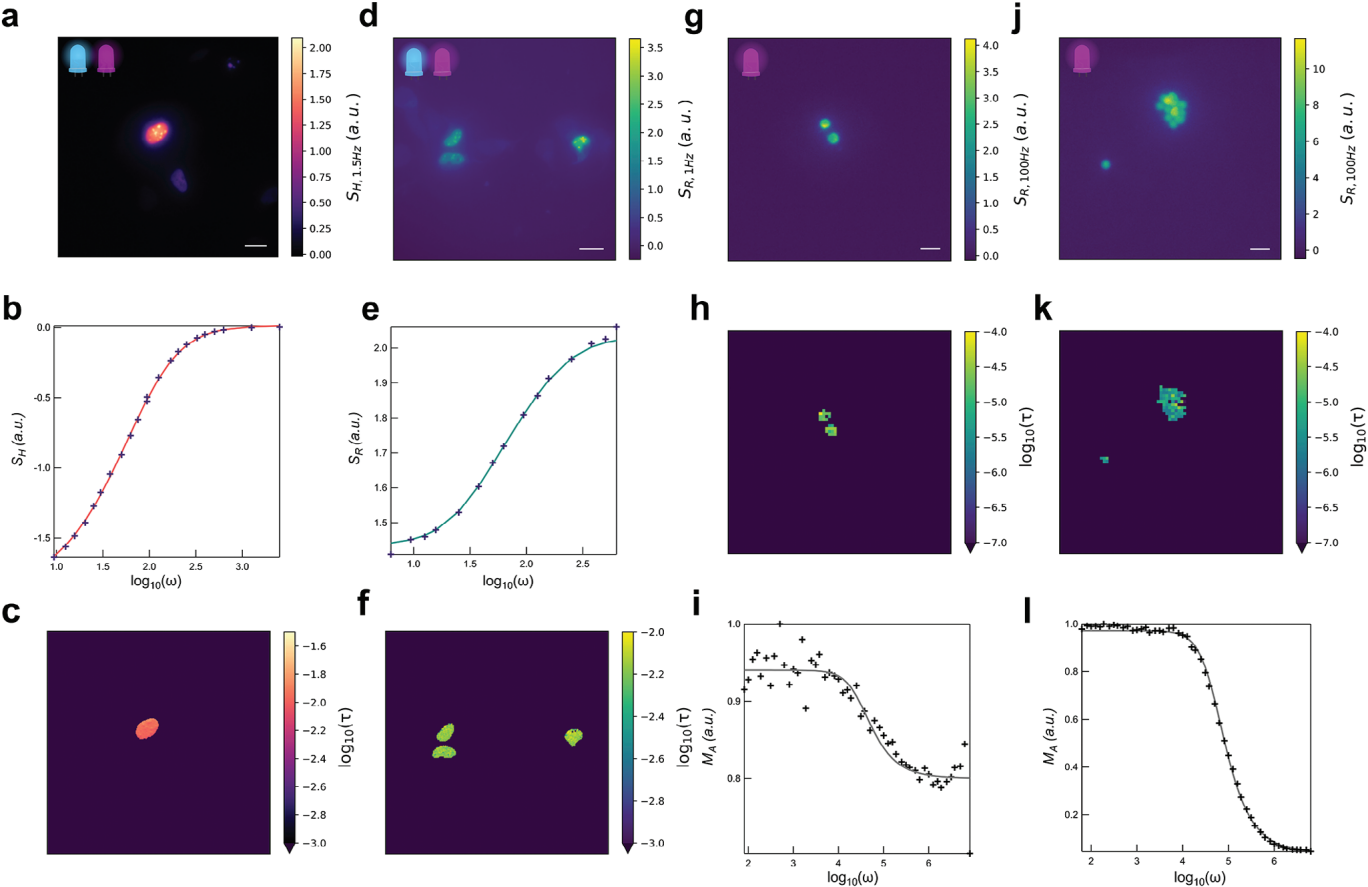
Validation of the HIOM and RIOM protocols. a,d) 12 Hz acquired image of the HIOM *S_H_* (a) and RIOM *S_R_* (d) observables collected at 525 nm from a fixed H2B‐Dronpa‐2 expressing HeLa cell under dual antiphase sinusoidally modulated illumination at 405 and 480 nm. Scale bars: 40 µm; b,e) *ω*‐Dependence of the HIOM (b) and RIOM (e) observables collected from the cell nucleus. Markers: Experiments, Solid line: Fit with Equations (74) (b) and (73) (e); c,f) HIOM (c) and RIOM (f) image of the Dronpa‐2 fluorescence photoswitching time; g,j) 12 Hz acquired images of the RIOM signal *S_R_* emitted at 675 nm from PdOEP‐(g) and PtOEP‐(j) loaded 4.5 µm diameter carboxylate polystyrene microbeads under sinusoidally modulated 405 nm light. Scale bar: 20 µm; h,k) RIOM obtained map of the phosphorescence lifetime retrieved from the microbeads displayed in g and j respectively; i,l) Reference synchronous detection obtained dependence of the phosphorescence signal at 675 nm from the PdOEP‐(i) and PtOEP‐(l) loaded 4.5 µm diameter carboxylate polystyrene microbeads on the frequency of the modulated excitation light at 540 nm. See Text and Tables S3–S7 (Supporting Information) for information on the parameters of acquisition and fitting. 2 independent repeats were performed for experiments (a,b,c), and 10 for experiments (d,e,f), (g,h,i), (j,k,l).

We first used our microscope to evaluate HIOM on fixed HeLa cells labeled at their nuclei with the fluorescent protein Dronpa‐2, which photoswitches from a bright to dark state under blue light, and vice versa under violet light.^[^ ^33^, ^34^, ^35^ ^]^ We recorded fluorescence images at 12 Hz acquisition frequency upon sinusoidally modulating 470 (blue) and 405 (violet) nm lights in antiphase around mean intensity levels of 0.25 and 0.13 E.m^−2^.s^−1^ (6.4 and 4.0 W.cm^−2^) with 100% amplitude, and with one illuminator at a frequency 1 Hz lower than the other (**Figure** **2**a). The *ω*‐dependence of the HIOM observable was fitted over the [1 Hz; 100 Hz] range (Figure 2b) to deliver a satisfactory image of the Dronpa‐2 photoswitching time *τ* (Figure 2c). We found a photoswitching time, *τ* = 12.8 ± 0.5 ms over the nucleus, in agreement with that expected, *τ* = 9.5 ± 1 ms, based on the light intensities used and the Dronpa‐2 photoswitching properties.^[^ ^32^, ^35^ ^]^

We then evaluated RIOM by imaging the same sample at 12 Hz acquisition frequency while applying sinusoidally modulated 470 and 405 nm lights in antiphase around 0.07 and 0.15 E.m^−2^.s^−1^ (1.8 and 6.4 W.cm^−2^) with 100% amplitude within the [1 Hz; 100 Hz] range (Figure 2d). After fitting the *ω*‐dependence of the RIOM observable at each pixel (Figure 2e), we retrieved a satisfactory image of the Dronpa‐2 photoswitching time (Figure 2f). We found *τ* = 8.2 ± 1.4 ms over the nucleus. Further data processing over 10 Dronpa‐2 labeled cells yielded 11 ± 3 ms for the mean value and standard deviation of the photoswitching time, in line with the *τ* = 10 ± 1 ms expected based on the light intensities used and the Dronpa‐2 photoswitching properties.^[^ ^32^, ^35^ ^]^

We could similarly build the wide‐field image of the photoswitching time of a Dronpa‐2 solution contained in the microchambers of a microfluidic device using the RIOM protocol on our fluorescence macroimager (see Figure S4a–c, Supporting Information). Here, slower kinetics were anticipated (*τ* = 545 ± 50 ms) due to the lower light intensities available on the instrument.^[^ ^32^, ^35^ ^]^ Our result with RIOM (*τ* = 485 ± 15 ms) was in agreement with the expected value. A further experiment was then performed, whereby RIOM was applied to image Dronpa‐2 solution at logarithmically decreasing concentrations (see Figure S5a–e, Supporting Information). Beyond showing that RIOM enables reliable extraction of the Dronpa‐2 photoswitching time down to the 0.1 µm range with this setup, we showed that this imaging protocol was as efficient as the Speed Out‐of‐Phase Imaging after Optical Modulation (OPIOM) protocol, which was established to exhibit superior sensitivity under adverse optical conditions.^[^ ^21^ ^]^

In the above validations, the illumination waveforms used were sinusoidal. However, HIOM and RIOM can also be implemented using other periodic modulation types. As a representative example, we explored square‐wave modulation, whereby, by extending our theoretical analysis, we showed that we could still build reliable fitting functions (see Sections 1, B, and D in the Supporting Information). Hence, we applied square‐wave modulated illumination with fixed HeLa cells labeled with Dronpa‐2 at their nuclei (Figure S6a–f, Supporting Information) and retrieved satisfactory HIOM‐ and RIOM‐maps of the photoswitching time: we respectively found *τ* = 8.8 ± 0.4 ms and *τ* = 14.4 ± 0.2 ms over the nucleus whereas *τ* = 11.4 ± 0.2 ms was anticipated.^[^ ^32^, ^35^ ^]^

After Dronpa‐2, we turned to luminophores endowed with faster photokinetics to evaluate the scope of our protocols. Hence, we used RIOM with our microscope to generate lifetime images from phosphors emitting phosphorescence after their photoactivation. We adopted Palladium octaethylporphyrin (PdOEP) and Platinum octaethylporphyrin (PtOEP), which are widely used as oxygen sensors and in organic light‐emitting diodes (OLEDs).^[^ ^36^, ^37^ ^]^ On a monolayer of immobilized 4.5 µm diameter carboxylate polystyrene microbeads, labeled with either PdOEP or PtOEP (Figure 2g,j), we applied 405 nm light, sinusoidally modulated around 8 and 30 mE.m^−2^.s^−1^ (0.24 and 0.91 W.cm^−2^), respectively, with 100% amplitude within the [10^2^,10^5^ Hz] range. After fitting the kinetic fingerprints at each pixel, we retrieved maps of PdOEP and PtOEP phosphorescence lifetimes (Figure 2h,k) which were in line with lifetime values found from direct measurement with synchronous detection (Figure 2i,l): *τ* = 21 ± 2 and *τ* = 34 ± 6 µs, respectively. A similar series of experiments was performed to extract the distribution of the luminescence lifetime of immobilized 3.0 µm diameter carboxylate polystyrene microbeads labeled with PtOEP and 4.5 µm diameter beads labeled with PdOEP (Figure S7, Supporting Information). Thus, we demonstrated that maps of lifetimes down to a few µs can satisfactorily be retrieved with our approach.

### 2.3 HIOM and RIOM Validation for Acquiring Kinetic Fingerprints

**Figure 3:**
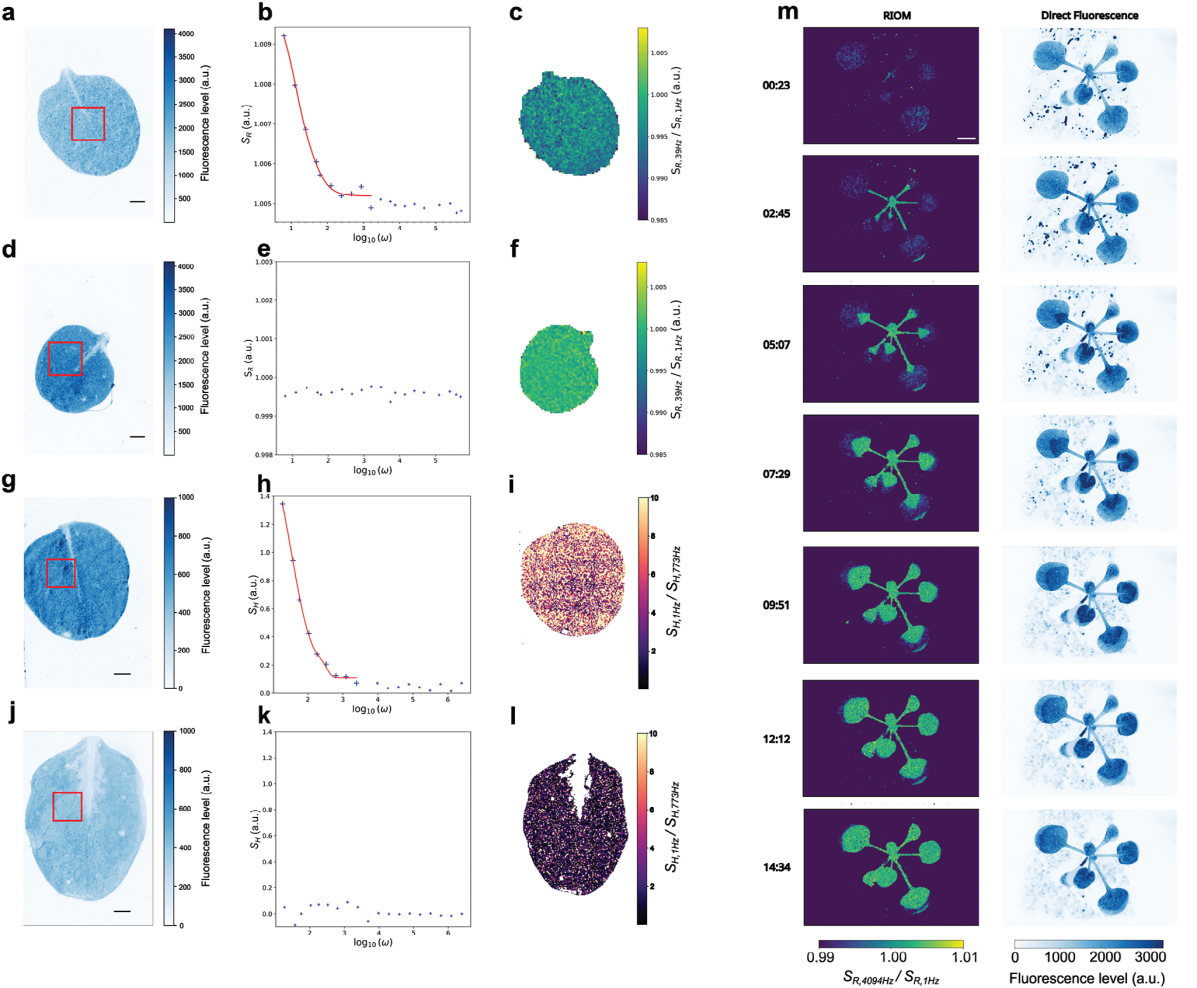
Application of the RIOM and HIOM protocols for reporting on the physiological state of plants. a,d) First macroscopic fluorescence image frame of the fluorescence level emitted at 690 nm acquired at 1 Hz from a leaf of wild‐type *Arabidopsis thaliana* (Columbia‐0) under sinusoidally modulated RIOM illumination at 470 nm and constant interfering light exhibiting a broad spectrum over [400, 800 nm] without (a) and with (d) DCMU application; b,e) *ω*‐Dependence of the RIOM signal *S_R_* emitted from the framed zone in a and d. Markers: experimental data; solid line: smoothing function eye guideline; c,f) Leaf image of the ratio of the RIOM signal at 1 Hz versus 39 Hz without (c) and with (f) DCMU application; g,j) First macroscopic fluorescence image frame of the fluorescence level emitted at 690 nm acquired at 12 Hz from a leaf of wild type *Arabidopsis thaliana* (Columbia‐0) under dual antiphase sinusoidally modulated HIOM illumination with two 470 nm LEDs and constant interfering light exhibiting a broad spectrum over [400, 800 nm] without (g) and with (j) DCMU application; h,k)* ω*‐Dependence of the HIOM signal *S_H_* emitted from the framed zone in g and j; i,l) Leaf image of the ratio of the HIOM signal at 1 Hz versus 773 Hz without (i) and with (l) DCMU application; m) Maps of the dynamic evolution of the ratio of the RIOM signals at 1 Hz versus 4094 Hz (left column) and direct fluorescence (right column) under sinusoidally modulated light at 470 nm and constant interfering light exhibiting a broad spectrum over [400, 800 nm] from a full wild type *Arabidopsis thaliana* (Columbia‐0) upon injection of a solution of DCMU into the soil, over the course of ≈15.5 h. Scale bar: a,d,g,j 1 mm; m 2 mm. See Text and Tables S3 and S4 (Supporting Information) for information on the parameters of acquisition. Independent repeats, >2 (a‐f); 2 (g‐l); 2 (m).

In cases where a lifetime is not sought, or where the luminophore's behavior cannot be described by a two‐state dynamic model, our protocols can still be of value for end users, allowing them to retrieve rich information in the form of kinetic fingerprints. In order to demonstrate their use for this, we applied them for the recovery of the photodynamic behavior of leaves, which can be of interest in remote imaging of the physiological state of plants. Endogenous fluorescence from the photosynthetic apparatus (**Figure** **3**a,d,g,j) is used to evidence stresses in plants from its light‐induced rise in the [1 ms, 1 s] range (fluorescence induction curves).^[^ ^38^ ^]^ However, the current protocols are restricted to dark conditions which are limiting for sensing applications^[^ ^38^ ^]^ and its imaging implementation has necessitated a fast camera.^[^ ^39^ ^]^ Hence, we evaluated HIOM and RIOM for collecting kinetic fingerprints of unstressed and stressed plants all the while being illuminated with interfering ambient light.

We first implemented RIOM (Figure 3a,d) and HIOM (Figure 3g,j) on leaves of wild‐type *Arabidopsis thaliana* using sinusoidally modulated 470 nm illumination, piloted over frequency ranges well covering the [1 ms; 1 s] time range used to acquire fluorescence induction curves: [1 Hz; 80 000 Hz] and [3 Hz; 393 221 Hz], respectively. In the case of HIOM, a second illuminator, modulated at a frequency 1 Hz lower than the other, was implemented. The mean light applied at 470 nm with both protocols was 100 µE.m^−2^.s^−1^ (2.6 mW.cm^−2^), and the oscillation amplitude was 100%. On top of the modulated illumination, a constant interfering light exhibiting a broad spectrum (400 to 800 nm), was applied. As displayed in Figure 3b,h, the kinetic fingerprints associated with reversible photoactivation of the photosynthetic apparatus drop above 10^2^ Hz (628 rad s^−1^) and then remain constant at higher frequencies. Interestingly, these drops disappeared when the leaf was stressed with 3‐(3,4‐dichlorophenyl)‐1,1‐dimethylurea (DCMU) (Figure 3e,k), a herbicide inhibiting photosynthesis. In the absence of a two‐state photoactivation model for photosynthesis, we used the ratio between the RIOM observable at high and low frequencies to report on the presence of DCMU in the leaves (Figure 3c,f,i,l). Using this, we performed a further experiment to follow the uptake and spread of DCMU in an *A. thaliana* plant exposed to DCMU at its roots (Figure 3m). The wave of increasing ratio, signifying the local presence of DCMU, smoothly progressed from the roots to the leaves, in lockstep with the wave of increased chlorophyll fluorescence (a measure used by others for reporting on local DCMU‐induced stress), and on a similar timescale (≈15 h) as seen in their work.^[^ ^40^ ^]^

Here, the kinetic fingerprints delivered by RIOM and HIOM intrinsically convey more discriminative information than solely the fluorescence level as a stress reporter for sensing the physiological state of plants: instead of evidencing stress through a change in endogenous fluorescence level, which is common to all the stresses, one can exploit the shape and position of entire Bode diagrams, such as those displayed in Figure 3b,e,h,k, for stress discrimination. Furthermore, contrary to endogenous fluorescence level, the RIOM/HIOM kinetic fingerprints are unaffected by interfering ambient light.

## 3 Discussion

The present work illustrates that the proper exploitation of the photocycle of the (photo)chemical reactions encountered by a reversibly photoactivable luminophore can remove the need to rely on costly detectors for the extraction of fast dynamic information.

We first demonstrated that a modulated intensifier is not required in order to retrieve luminescence kinetic information in both homodyne (with RIOM) and heterodyne (with HIOM) modes.

We also evidenced that the frequency dependence of the RIOM/HIOM signals yields this luminescence kinetic information even in the presence of ambient light. In macroscale luminescence imaging,^[^ ^21^ ^]^ such a goal usually requires sophisticated equipment involving modulated illumination and image acquisition in homodyne detection^[^ ^41^, ^42^ ^]^ and pulsed excitation and time‐gated detection.^[^ ^43^, ^44^ ^]^ This goal is here achieved by exploiting the difference of frequencies involved in the applied RIOM/HIOM modulated illumination and in the fluctuations of ambient light. However, ambient light can contribute to driving the mean extent of photoactivation of a reversibly photoactivable luminophore. Hence, its contribution should favorably be taken into account to fix the mean light intensities of the applied modulated illumination to benefit from optimal signals in RIOM and HIOM.

HIOM and RIOM exhibit interesting complementarities. RIOM is simpler to implement and process than HIOM, which requires cross‐term and aliasing‐free dual‐frequency light excitation and Fourier Transform analysis. In contrast, although both protocols can overcome the detrimental interference of ambient light for retrieving kinetic information, RIOM is only relevant when ambient light does not change during the measurement whereas HIOM can deal with changing ambient light provided that it does so at a frequency lower than *Δω*.

## 4 Conclusion

We demonstrated that the HIOM and RIOM imaging protocols implemented on low‐cost setups featuring standard cameras can reliably deliver wide‐field maps of luminescence lifetime down to the microsecond range for reversibly photo‐convertible luminophores engaged in a two‐state photodynamic exchange, which is often appropriate. When the detailed photochemical mechanisms are not available, HIOM and RIOM more generally provide kinetic fingerprints, which contain rich and easily exploitable information as here exemplified by discriminating the plant's physiological state even under interfering ambient light.

## 5 Experimental Section

### Production of the Samples of Metal Complex‐Loaded Polystyrene Microbeads

A total of 1 mm stock solutions of platinum octaethylporphyrin (PtOEP; Sigma‐Aldrich, Burlington, MA, US) and palladium octaethylporphyrin (PdOEP; Sigma‐Aldrich, Burlington, MA, US) in tetrahydrofuran (THF; Sigma‐Aldrich, Burlington, MA, US) were prepared and stored at 4 °C. They were always sonicated for 5 min before use.

Two batches of 4.50 µm diameter carboxylated polystyrene microbeads were produced, one loaded with PtOEP and the other with PdOEP. 140 µL of the 1 mm THF stock solution was mixed at 500 rpm with 260 µL of ultrapure water and 100 µL of a 2.5% (w/w) polystyrene bead suspension (Polybead Carboxylate Microspheres 4.50 µm; Polysciences Inc., Warrington, PA, US). The resulting suspensions were then dialyzed overnight in a 0.5–3 mL dialysis cassette (Slide A‐Lyzer; Thermo Scientific Inc., Waltham, MA, US), with a 3.5 K molecular weight cutoff (MWCO), immersed in ultrapure water. The suspensions of dye‐loaded polystyrene beads were stored in sealed vials at 4 °C. The preceding suspensions were used to prepare a monolayer of metal complex‐loaded polystyrene beads immobilized on an agarose pad. 125 µL of low‐gelling temperature agarose (Sigma‐Aldrich, Burlington, MA, US) in PBS (pH 7.4, 50 mm sodium phosphate, 150 mm NaCl) buffer was deposited in a microchamber delimited with a 250 µm‐thick spacer (Gene Frames AB0578; Thermo Scientific Inc., Waltham, MA, US) on a circular glass coverslip and the microchamber was capped with a second circular glass coverslip. After 15 min at 4 °C, the agarose became solid and the upper coverslip was removed. 1 µL of the suspension of metal complex‐loaded polystyrene beads was then deposited on the surface of the agarose pad. After 15 min, the top coverslip was replaced to seal the sample.

### Production and Purification of Dronpa‐2

The Dronpa‐2 plasmid with an N‐terminal hexahistidine tag was transformed in the *E. coli* TOP10 strain. Cells were grown in Terrific Broth at 37 °C. The expression was induced at 16 °C by the addition of IPTG to a final concentration of 1 mm at an optical density of 0.6 at 600 nm. The cells were collected after 16 h of expression and lysed by sonication in lysis buffer (50 mm PBS with 150 mm NaCl at pH 7.4, 1 mg.mL*^−^* ^1^ DNAse, 5 mm MgCl_2_ and 1 mm phenyl‐methylsulfonyl fluoride, and a cocktail of protease inhibitors [0 469 313 2001, Roche, Basel, Switzerland]). After lysis, the mixture was incubated on ice for 2 h for DNA digestion. The insoluble material was removed by centrifugation and the supernatant was incubated overnight with Ni‐NTA agarose beads (Thermo Scientific Inc., Waltham, MA, US) at 4 °C in a rotary mixer. The protein‐loaded Ni‐NTA column was washed with 20 column volumes of N1 buffer (50 mm PBS, 150 mm NaCl, 20 mm imidazole, pH 7.4) and 5 column volumes of N2 buffer (50 mm PBS, 150 mm NaCl, 40 mm imidazole, pH 7.4). The bound protein was eluted with N3 buffer (50 mm PBS, 150 mm NaCl, 0.5 m imidazole, pH 7.4). Imidazole was removed from the protein fractions using a PD‐10 column (Cytiva, Marlborough, MA, US) and replaced with 50 mm PBS buffer (pH 7.4, 150 mm NaCl).

### Production of Dronpa‐2‐Labeled Mammalian Cells

Hela cells were grown at 37 °C in DMEM high glucose media complemented with 10% fetal bovine serum (Thermo Scientific Inc., Waltham, MA, US), and air with an increased CO_2_ concentration (5%). The cells were transiently transfected with GeneJuice (Sigma‐Aldrich, Burlington, MA, US), according to the manufacturer's protocol, then washed with DPBS (Thermo Scientific Inc., Waltham, MA, US) and fixed with 2% paraformaldehyde solution in DPBS.

### Cultivation of Arabidopsis Thaliana

*Arabidopsis thaliana* (Columbia‐0) plants were grown under controlled conditions: photoperiod = 8 h (9h00‐17h00), day temperature = 21 °C, night temperature = 17–21 °C, light intensity = 100 µE.m*^−^* ^2^.s*^−^* ^1^ (white light).

### Microfluidic Device

The microfluidic device was composed of a circular glass coverslip (0.17 mm thick, 40 mm diameter; Thermo Scientific Inc., Waltham, MA, US) and a PDMS stamp (RTV615; General Electric, Fairfield, CT, US) including six 250 µm *×* 125 µm *×* 20 µm chambers separated by 100 µm *×* 20 µm walls. Each chamber was connected, by a 25 µm *×* 20 µm channel, to a sample reservoir (punched into the PDMS stamp). Before assembly, the coverslip and the PDMS stamp were rinsed with ethanol and dried under an airflow. The glass surface of the microdevice is oriented toward the imaging objective for acquiring images. In order to fill the micro‐chambers with the desired solutions, the PDMS stamp was first placed under a vacuum (50 mbar) for 3 min to remove dissolved air before the device's reservoirs were filled with the sample solutions, resulting in the autonomous uptake of the solutions into the microchamber.

### Epifluorescence Microscope

The setup is equipped with several light sources: a UV 405 nm LED (LHUV‐0405, Lumileds, San Jose, CA), two blue 470 nm LEDs (LXZ1‐PB01, Lumileds, San Jose, CA) and two green 540 nm LEDs (L1C1‐LME1000000000, Lumileds, San Jose, CA). The LEDs are individually powered by LED drivers (LEDD1B, Thorlabs, Newton, NJ), a pair of which can be modulated with a desired phase and frequency using a waveform generator (33612A, Keysight Technologies, Santa Rosa, CA). The light from each LED is collimated using a high numerical aperture condenser lens (ACL25416U, Thorlabs Inc., Newton, NJ; *f* = 16 mm). For the blue and green LED pairs, their collimated light beams are first combined using a 50:50 beam splitter (CCM1‐BS013, Thorlabs Inc., Newton, NJ, US) before being filtered with a corresponding excitation filter (blue = HQ480/40, green = ET540/40; Chroma Technology Corp., Bellows Falls, VT, US). The two resulting light beams are then combined with a dichroic mirror (FF506‐Di03, IDEX Health & Science LLC, Rochester, NY) into a single beam which passes through a second dichroic mirror (T425LPXR, Chroma Technology Corp., Bellows Falls, VT, US) to mix with the collimated light beam coming from the UV LED, filtered using a bandpass filter (ZET405/20X, Chroma Technology Corp., Bellows Falls, VT, US). An achromatic doublet (*f* = 75 mm; AC254‐075‐A, Thorlabs Inc., Newton, NJ, US) is used to focus the collimated light to the back focal plane of the objective, after being reflected by a dichroic filter with its edge wavelength at either 506 nm (FF506‐Di03, IDEX Health & Science LLC, Rochester, NY), for when working with Dronpa‐2 containing samples, or 560 nm (FF560‐Di01, IDEX Health & Science LLC), for dye‐loaded beads. Depending on the magnification desired, the objective used was either a 10*×* (Fluar, Zeiss, Jena, Germany; N.A. 0.5) or a 50*×* (MPlanFLN, Olympus Corp., Tokyo, Japan; N.A. 0.8) objective. Fluorescence emission was collected by the objective; filtered by a bandpass filter, centered at either 525 nm (FF01‐525/30, IDEX Health & Science LLC, Rochester, NY), for Dronpa‐2 containing samples, or 675 nm (FF01‐675‐67, IDEX Health & Science LLC, Rochester, NY), for dye‐loaded beads; and reflected by a mirror (MPG01‐350‐700, IDEX Health & Science LLC, Rochester, NY) before being refocused onto the sensor of an iXon 897 EMCCD camera (Andor Technology, Belfast, UK) by an *f* = 150 mm achromatic doublet (ACA254‐150‐A, Thorlabs Inc., Newton, NJ, US).

It is also possible with this setup to record the overall luminescence signal from the sample with a fast photomultiplier tube point detector (H10492‐002, Hamamatsu, Hamamatsu City, Japan), enabling synchronous detection‐based measurement of the luminescence lifetime. In order to allow for this, a portion of the emitted light is separated out with a 50:50 beam splitter (CCM1‐BS013, Thorlabs Inc., Newton, NJ, US) and delivered to the detector. In the synchronous detection‐based measurement protocol, the excitation light is modulated and the light emitted from the sample is demodulated with synchronous detection with a lock‐in amplifier (HF2LI 50 MHz, Zurich instruments, Zurich, Switzerland).

### Fluorescence Macroimager

An image of the setup, and a diagram of its optical components, shown in the form of a computer‐rendered CAD model, created in Rhinoceros 3D (Robert McNeel & Associates, Seattle, WA, US), are shown in Figure S3 (Supporting Information). These components are held using a mixture of standard optomechanical and 3D‐printed components. The details of each and every component, aside from the relevant optics and electronics are not detailed here. One element that is worth mentioning is that the instrument features a dark box, used to prevent environmental light from reaching the sample and interfering with the measurements. The setup consists of two illumination paths and one imaging path, with their optical axes separated. The imaging path, vertically aligned, consists of a macroscope objective (1X/WF, Nikon, Tokyo, Japan), to collimate the light originating from the sample plane; an emission filter, selectable depending on the luminophore, to pass only the luminesced light; and a camera objective (AF Nikkor 50 mm f/1.8D, Nikon, Tokyo, Japan) to focus the collimated light onto the image sensor of 1936 *×* 1216 pixels [11.345 mm *×* 7.126 mm] greyscale global‐shutter camera (UI‐3060CP‐M‐GL, IDS Imaging Development Systems GmbH, Obersulm, Germany). Regarding the illumination system, the two arms of the system each project light onto the sample at an angle of 30° vertical and are offset by an azimuth angle of 40° from each other.

The LEDs, and accordingly the filters and dichroic mirror, present in the illumination arms were different in the different experiments. The description of the optical components of the illumination arms which follow, refers to the configuration used in the experiment with the Dronpa‐2 sample. The modifications made for the experiments with *Arabidopsis thaliana* are detailed in the paragraph which follows. The path of light from one of the LEDs, a blue LED (470 nm, L1RXBLU1000000000, Lumileds, San Jose, CA, USA), labeled as object 15 in Figure S3 (Supporting Information), will be referred to first. The light from this LED is collimated using a high numerical aperture condenser lens (ACL25416U, Thorlabs Inc., Newton, NJ, US), before being filtered with a corresponding excitation filter (ET470/40X, Chroma Technology Corp., Bellows Falls, VT, US). After passing through the dichroic mirror (T425LPXR, Chroma Technology Corp., Bellows Falls, VT, US), the light is focused onto the small 2.5 *×* 2.5 mm end of a tapered light pipe (65‐840, Edmund Optics Inc., Barrington, NJ, US) using a plano‐convex lens (LA1805‐A, Thorlabs Inc., Newton, NJ, US). The light pipe is used to homogenize the light injected into it, through multiple internal reflections, providing homogeneous light over the pipe's 5 *×* 5 mm exit face. The lens assembly used after, a matched achromatic doublet pair (MAP1040100‐A, Thorlabs Inc., Newton, NJ, US), conjugates the exit face of the light pipe to the sample plane, illuminating the sample plane with an ≈12.5 *×* 12.5 mm illumination. The same illumination arm features a second LED, marked as object 9. It is a 405 nm LED (LHUV‐0405‐A065, Lumileds, San Jose, CA, USA), which is coupled with an appropriate excitation filter (ZET405/20X, Chroma Technology Corp., Bellows Falls, VT, US). The light from this LED, after collimation and filtering, is reflected by the dichroic mirror and also injected into the light pipe, where it then gets delivered uniformly to the sample in the same manner as was detailed for the blue LED. On the other illumination arm, a second of the same 405 nm LED and corresponding filter were used, in order to allow for higher light intensities to be achieved at 405 nm.

As was mentioned in the previous paragraph, the LEDs, filters, and dichroic used in the experiments with *Arabidopsis thaliana* were different from those used in the experiments with Dronpa‐2. The modifications made are detailed here. The LEDs were arranged such that the left illumination arm contained one blue LED (the same as that detailed above), used in combination with the same corresponding excitation filter as that detailed above; and the right illumination arm contained a second of the same type of blue LED and corresponding filter, together with a green LED (L1C1‐LME1000000000, Lumileds, San Jose, CA, USA), used with no excitation filter. A dichroic mirror (FF506‐Di03‐25 *×* 36, IDEX Health & Science LLC, Rochester, NY, US) was used to combine the light from the sources in the right illumination arm.

The LEDs were driven in different ways, depending on the experiment. In the work with Dronpa‐2, high values of intensity were desired, therefore the LEDs were driven using an LED driver (DC4104, Thorlabs Inc., Newton, NJ, US), whose outputs were modulated using signals from a waveform generator (T3AFG80, Teledyne Technologies, Thousand Oaks, CA, USA). In the case of the experiments with *Arabidopsis*, where lower light intensities were desired, the LEDs were driven directly with the waveform generator. In all cases, sinusoidal modulation commencement on the waveform generator, and the triggering of camera frames were achieved using trigger signals generated using a data acquisition board (DAQ; USB‐1604HS‐2AO, Digilent, Pullman, WA, USA).

### Light Intensity Measurement

The light intensities on the imaging setups were measured either by exploiting fluorescent actinometers^[^ ^32^ ^]^ or by using a power meter.

### Data Processing

In the frame of a two‐state photodynamic model, two fitting functions are used to retrieve the characteristic time *τ* from the dependence of the observables of RIOM and HIOM on the applied angular frequency/ies: i) In RIOM, the three floating parameters are adopted fitting function given in Equation (1):

(1) $$S_{R}=\frac{p_{1}\left(1+4{\left(\omega \tau \right)}^{2}\right)}{p_{2}+5{\left(\omega \tau \right)}^{2}+4{\left(\omega \tau \right)}^{4}}+p_{3}$$

ii) In HIOM, the three floating parameters are adopted fitting function given in Equation (2):

(2) $$S_{H}=\frac{p_{1}\left(2+3\left(\gamma \omega \tau \right)+3{\left(\gamma \omega \tau \right)}^{2}\right)}{p_{2}+8\left(\gamma \omega \tau \right)+12{\left(\gamma \omega \tau \right)}^{2}+8{\left(\gamma \omega \tau \right)}^{3}+4{\left(\gamma \omega \tau \right)}^{4}}+p_{3}$$

where γ  =  1 and γ  =  1.85 with sine‐wave and square‐wave modulated illumination respectively.

### Statistical Analysis

The number of repeats performed for each luminophore/technique is indicated at the appropriate positions in the text and figure captions. In cases where the characteristic time is reported for a single experiment, the error given corresponds to the standard deviation reported from the fitting procedure. Where it is reported for a number of repeat experiments, its value corresponds to the mean, and the error is the standard deviation over those repeats. The errors given for theoretically predicted characteristic times are the errors reported in the literature. The manner in which the raw experimental data were processed for each luminophore/technique, too extensive to detail here, is provided in great detail in Section 2 of the Supporting Information. Such processing was performed using codes written in the programming language Python. These have been made available via the DOI indicated in the Code availability section to enable the reproduction of the data analysis.

## Conflict of Interest

The authors declare no conflict of interest.

## Author Contributions

T. L. S. and L. J. performed conceptualization. T. L. S. and L. J. performed the methodology. I. C., A.L., T. L. S., and H. M.developed software.; I. C., A. L., T. L. S., and H. M. performed validation. I. C., A. L., T. L. S., H. M. performed formal analysis. I. C., A. L., T. L. S., and H. M. performed the investigation. H. M. and M.‐A. P. managed resources. I. C., A. L., T. L. S., and H. M. performed data curation. I. C., L. J., T. L. S., and H. M. wrote the original draft. I. C., L. J., A. L., T. L. S., H. M. performed writing – review & editing. I. C., L. J., A. L., T. L. S., and H. M. performed visualization. L. J. and T. L. S. performed supervision. L. J. performed project administration. L. J. and T. L. S. performed funding acquisition.

## Supporting information



Supporting Information

## Data Availability

The data that support the findings of this study are available from the corresponding author upon reasonable request.
