# Supplementary material for: Periodic Light Modulations for Low‐Cost Wide‐Field Imaging of Luminescence Kinetics Under Ambient Light
Source: Adv Sci (Weinh). 2025 Jan 17;12(10):2413291. doi: 10.1002/advs.202413291 (PMC11904971; doi:10.1002/advs.202413291)
Supplement: Supplementary file 1 — Supporting Information [file ADVS-12-2413291-s001.pdf]

ADVANCED SCIENCE
Open Access

Supporting Information

for *Adv. Sci.*, DOI 10.1002/advs.202413291

Periodic Light Modulations for Low-Cost Wide-Field Imaging of Luminescence Kinetics Under Ambient Light

Hélène Merceron, Ian Coghill, Aliénor Lahlou, Marie-Aude Plamont, Ludovic Jullien and Thomas Le Saux**

Periodic light modulations for low cost wide-field imaging of luminescence kinetics under ambient light

Hélène Merceron,[1,2,†] Ian Coghill,[1,†] Aliénor Lahlou,[1,3] Marie-Aude Plamont,[1]
Ludovic Jullien,[1,*] Thomas Le Saux,[1,*]

December 3, 2024

(1) PASTEUR, Départment de Chimie, École normale supérieure, PSL Research University, Sorbonne Université, CNRS, 24, rue Lhomond, 75005 Paris, France

(2) IMPMC UMR 7590 Sorbonne Université, CNRS, MNHN, IRD, Museum National d'Histoire Naturelle, 61 rue Buffon 75005 Paris France.

(3) Sony Computer Science Laboratories, Paris, France.

[†] Co-first authors.

Corresponding authors: Ludovic.Jullien@ens.psl.eu, Thomas.Lesaux@ens.psl.eu

Contents

1 Theoretical responses of reversibly photo-convertible luminophores to modulated illuminations 4

1.1 The model . . . 4

1.2 Response to periodic light modulation . . . 4

1.2.1 Master equation . . . 4

1.2.2 Concentrations . . . 6

1.2.3 Luminescence intensity . . . 8

1.2.3.1 General expressions . . . 8

1.2.3.2 Temporal dependences of the modulated illumination . . . 9

1.2.3.3 Observables of RIOM and HIOM . . . 10

1.2.3.4 Resonance conditions of the observables of RIOM and HIOM . . . 10

1.3 Extraction of the characteristic time associated with the photo-conversion . . . 11

1.4 Implementation of the RIOM and HIOM protocols for luminescence lifetime imaging . . . 12

1.4.1 Luminophores . . . 12

1.4.2 Equipments . . . 12

1.4.3 Procedure . . . 13

1.4.3.1 Generic HIOM protocol (Figure S1) 13
1.4.3.2 Generic RIOM protocol (Figure S1) 15

2 Materials and methods 16

2.1 Instruments 16
2.1.1 Epifluorescence microscope 16
2.1.2 Fluorescence macroimager 16
2.1.3 Measurement of light intensity 17
2.1.3.1 Epifluorescence microscope 17
2.1.3.2 Fluorescence macroimager 18
2.1.4 Generation of harmonics-free sine wave modulated illumination 18
2.1.5 Acquisition protocols 18
2.1.5.1 Dronpa-2 labeled cells under sine-wave modulated illumination 18
2.1.5.2 Dronpa-2 Filled Microfluidic Chambers under sinusoidal modulated illumination . . . 18
2.1.5.3 Droplets of Dronpa-2 solution at various concentrations under sinusoidal modulated illumination 19
2.1.5.4 Dronpa-2 labeled cells under square-wave modulated illumination 22
2.1.5.5 Luminescent metal complexes and dyes embedded in polystyrene beads 24
2.1.5.6 Arabidopsis Thaliana 27
2.1.6 Acquisition parameters used for HIOM and RIOM imaging 31
2.1.7 Fitting parameters used in HIOM and RIOM images 32

A Sinusoidal modulation at a single angular frequency 33

A.1 Light modulation at a single wavelength 33
A.1.1 Generic maps 33
A.1.2 Dimensionalized maps 34
A.1.2.1 Phosphorescent probes 35
A.1.2.2 Reversibly photo-convertible fluorophores 36
A.2 Light modulation at two wavelengths 38
A.2.1 Significance of the phase lag φ 38
A.2.2 Generic maps 41
A.2.3 Dimensionalized maps 42

B Square-wave modulation at a single angular frequency 44

B.1 Light modulation at a single wavelength 44

B.1.1 Generic maps 45
B.1.2 Dimensionalized maps 46
B.1.2.1 Phosphorescent probes 46
B.1.2.2 Reversibly photo-convertible fluorophores 48
B.2 Light modulation at two wavelengths 50
B.2.1 Generic maps 51
B.2.2 Dimensionalized maps 52

C Sinusoidal modulation at two angular frequencies **53**
C.1 Significance of the phase lag φ 53
C.2 Generic maps 55
C.3 Dimensionalized maps 56
C.3.1 Phosphorescent probes 56
C.3.2 Reversibly photo-convertible fluorophores 58

D Square-wave modulation at two angular frequencies **60**
D.1 Generic maps 61
D.2 Dimensionalized maps 62
D.2.1 Phosphorescent probes 62
D.2.2 Reversibly photo-convertible fluorophores 64

E Reduction of photo(physical)chemical mechanisms to a two-state exchange **66**
E.1 A two-state electronic model 66
E.2 A three-state electronic model 67
E.3 A reversibly photo-convertible luminophore 69

1 Theoretical responses of reversibly photo-convertible luminophores to modulated illuminations

1.1 The model

The dynamic behavior of a reversibly photo-convertible luminescent probe P illuminated with a light of intensity $I(t)$ involving two components $I_1(t)$ and $I_2(t)$ at wavelengths λ_1 and λ_2 is assumed to be reliably described by the two-state exchange (1) in the range of investigated frequencies of light modulation

$$\mathsf{1} \quad \underset{k_{21}(t)}{\overset{k_{12}(t)}{\rightleftharpoons}} \quad \mathsf{2} \tag{1}$$

In the reaction (1), the thermodynamically most stable state $\mathsf{1}$ is photochemically converted to the thermodynamically less stable state $\mathsf{2}$ at rate constant $k_{12}(t) = \sigma_{12,1}I_1(t) + \sigma_{12,2}I_2(t)$ from which it can relax back to the initial state $\mathsf{1}$ either by a photochemically- or a thermally-driven process at rate constant $k_{21}(t) = \sigma_{21,1}I_1(t) + \sigma_{21,2}I_2(t) + k_{21}^{\Delta}$ where $\sigma_{12,1}I_1(t)$, $\sigma_{12,2}I_2(t)$, $\sigma_{21,1}I_1(t)$, $\sigma_{21,2}I_2(t)$, and k_{21}^{Δ} are respectively the photochemical and the thermal contributions of the rate constants. In that case, the molecular action cross-sections for photoisomerization $\sigma_{12,1}$ and $\sigma_{21,1}$ (at λ_1), $\sigma_{12,2}$ and $\sigma_{21,2}$ (at λ_2), and the thermal rate constant k_{21}^{Δ} fully define the behavior of the photo-convertible probe. In the following, λ_1 and λ_2 can be either different or identical. Similarly, the components of illumination $I_1(t)$ and $I_2(t)$ can as well more favorably promote the photoconversion of the state **1** to the state **2**, or the one of the state **2** to the state **1**.

We assume that the system is closed and either uniformly illuminated or that it can be considered homogeneous at any time of its evolution. Then we rely on the two-state exchange (1) to write Eqs.(2–3) describing the concentration evolutions:

$$\frac{d1}{dt} = -k_{12}(t)\,1 + k_{21}(t)\,2 \tag{2}$$

$$\frac{d2}{dt} = k_{12}(t)\,1 - k_{21}(t)\,2. \tag{3}$$

1.2 Response to periodic light modulation

In the following subsections, we analyze the response of the luminescence emission from the reversibly photo-convertible luminescent probe P when it is submitted to various periodic light modulations. We first introduce the principle of theoretical analysis, which is common to all the presently considered periodic light modulations. Then we apply this theoretical analysis to specific cases.

1.2.1 Master equation

We consider that the reversibly photo-convertible luminescent probe P is submitted to periodic illumination. To be at the most general in the following, illumination is assume to involve two components: a periodic illumination $I_1(t)$ at

wavelength λ_1 with average light intensity I_1^0 and a periodic illumination $I_2(t)$ at wavelength λ_2 with average light intensity I_2^0.

In the most general case, we write

$$I(t) = I_1(t) + I_2(t) \tag{4}$$
$$I_1(t) = I_1^0\left[1 + \alpha h_1(t)\right] \tag{5}$$
$$I_2(t) = I_2^0\left[1 + \alpha\delta h_2(t)\right] \tag{6}$$

with δ = 0 or 1. In Eqs.(5,6), α and $\alpha\delta$ measure the amplitude of light modulation, and $h_1(t)$ and $h_2(t)$ designate periodic functions. The following theoretical analysis notably enables us to cover the case of the absence of illumination at wavelength λ_2 (by making $I_2^0 = 0$) or the case of non-modulated illumination at wavelength λ_2 (by making δ = 0).

We introduce the notations

$$I^0 = I_1^0 + I_2^0 \tag{7}$$
$$k_{12}^0 = k_{12,1}^0 + k_{12,2}^0 \tag{8}$$
$$k_{21}^0 = k_{21,1}^0 + k_{21,2}^0 + k_{21}^\Delta \tag{9}$$

where

$$k_{12,1}^0 = \sigma_{12,1} I_1^0 \tag{10}$$
$$k_{21,1}^0 = \sigma_{21,1} I_1^0 \tag{11}$$
$$k_{12,2}^0 = \sigma_{12,2} I_2^0 \tag{12}$$
$$k_{21,2}^0 = \sigma_{21,2} I_2^0 \tag{13}$$

Eqs.(4,5,6) are used to express the rate constants with Eqs.(14,15)

$$k_{12}(t) = k_{12,1}^0\left[1 + \alpha h_1(t)\right] + k_{12,2}^0\left[1 + \alpha\delta h_2(t)\right] \tag{14}$$
$$k_{21}(t) = k_{21,1}^0\left[1 + \alpha h_1(t)\right] + k_{21,2}^0\left[1 + \alpha\delta h_2(t)\right] + k_{21}^\Delta. \tag{15}$$

Then we introduce

$$\tau_{12}^0 = \frac{1}{k_{12}^0 + k_{21}^0} \tag{16}$$

which designates the relaxation time of the reversibly photo-convertible luminophore when it is submitted to illumination at constant light intensity I^0, and 1^0 and 2^0 the concentrations of **1** and **2** at the associated photostationnary state reached after τ_{12}^0

$$1^0 = P_{\rm tot} - 2^0 = \frac{1}{1 + K_{12}^0} P_{\rm tot} \tag{17}$$

where

$$K_{12}^0 = \frac{k_{12}^0}{k_{21}^0} \tag{18}$$

and the total concentration in reversibly photo-convertible probe P, $P_{\text{tot}} = 1 + 2$.

Upon expanding the concentration expressions by introducing the function $f(t)$

$$2 = 2^0 + \alpha f(t) \tag{19}$$

$$1 = 1^0 - \alpha f(t), \tag{20}$$

the system of differential equations governing the temporal evolution of the concentrations in 1 and 2 is solved with Eqs.(2,3) to yield

$$\frac{df(x)}{dx} = -f(x) + [\mathfrak{a}_1 - \mathfrak{b}_1 f(x)]\, h_1(x) + \delta\, [\mathfrak{a}_2 - \mathfrak{b}_2 f(x)]\, h_2(x) \tag{21}$$

where

$$x = \frac{t}{\tau_{12}^0} \tag{22}$$

$$\mathfrak{a}_1 = \rho_{12}^0 \Delta_{12,1}^0 \tau_{12}^0 \tag{23}$$

$$\mathfrak{b}_1 = \alpha\left(\sigma_{12,1} + \sigma_{21,1}\right) I_1^0 \tau_{12}^0 \tag{24}$$

$$\mathfrak{a}_2 = \rho_{12}^0 \Delta_{12,2}^0 \tau_{12}^0 \tag{25}$$

$$\mathfrak{b}_2 = \alpha\left(\sigma_{12,2} + \sigma_{21,2}\right) I_2^0 \tau_{12}^0 \tag{26}$$

and

$$\rho_{12}^0 = k_{12}^0 1^0 = k_{21}^0 2^0 \tag{27}$$

$$\Delta_{12,1}^0 = \frac{k_{12,1}^0}{k_{12,1}^0 + k_{12,2}^0} - \frac{k_{21,1}^0}{k_{21,1}^0 + k_{21,2}^0 + k_{21}^\Delta} \tag{28}$$

$$\Delta_{12,2}^0 = \frac{k_{12,2}^0}{k_{12,1}^0 + k_{12,2}^0} - \frac{k_{21,2}^0}{k_{21,1}^0 + k_{21,2}^0 + k_{21}^\Delta} \tag{29}$$

designate the steady-state rate of reaction (1) and the differences of the relative contributions of the average of the modulated light (I_1^0 and I_2^0 respectively) to drive the transition from 1 to 2 or from 2 to 1 respectively.

1.2.2 Concentrations

Beyond the relaxation time τ_{12}^0, one enters into a permanent regime in which $f(x)$ is a continuous periodic function. In the cases considered in this work, light modulation involves one (denoted ω) or two (denoted ω_1 and ω_2) fundamental modulation frequencies.

In the first situation, the Fourier series associated to $f(x)$ can be written

$$f(\theta x) = a^0 + \sum_{n=1}^{+\infty} \left[a^{n,\cos} \cos(n\theta x) + b^{n,\sin} \sin(n\theta x)\right] \tag{30}$$

where

$$\theta = \omega\tau_{12}^0, \tag{31}$$

and $a^{n,\cos}$ and $b^{n,\sin}$ designate the amplitudes of the n-th components of the Fourier series.

In contrast, in the second situation, the Fourier series associated to $f(x)$ can be expressed as

$$f(\theta_1 x, \theta_2 x) = \sum_{n=0}^{+\infty} \sum_{m=-\infty}^{+\infty} \left\{a^{n,m,\cos} \cos[(n\theta_1 + m\theta_2)x] + b^{n,m,\sin} \sin[(n\theta_1 + m\theta_2)x]\right\} \tag{32}$$

where

$$\theta_1 = \omega_1\tau_{12}^0 \tag{33}$$

$$\theta_2 = \omega_2\tau_{12}^0 \tag{34}$$

and $a^0 = a^{0,0,\cos}$, $a^{n,m,\cos}$, and $b^{n,m,\sin}$ designate the amplitudes of the zeroth and $\{n,m\}$-th components of the Fourier series.

Either the $a^{n,\cos}$ and $b^{n,\sin}$, or the a^0, $a^{n,m,\cos}$, and $b^{n,m,\sin}$ terms can be extracted from Eq.(21) upon identifying the amplitudes of the components of the same order (harmonic balance). The resulting set of equations can then be transformed to explicit the amplitudes of the concentration modulations at all modulation frequencies. Thus we write either

$$2 = \mathtt{2}^{\mathtt{o}} + \alpha \sum_{n=1}^{+\infty} \left[\mathtt{2}^{\mathtt{n},\sin} \sin(n\theta x) + \mathtt{2}^{\mathtt{n},\cos} \cos(n\theta x)\right] \tag{35}$$

$$1 = \mathtt{1}^{\mathtt{o}} - \alpha \sum_{n=1}^{+\infty} \left[\mathtt{2}^{\mathtt{n},\sin} \sin(n\theta x) + \mathtt{2}^{\mathtt{n},\cos} \cos(n\theta x)\right] \tag{36}$$

where

$$\mathtt{2}^{\mathtt{o}} = 2^0 + \alpha a^0 \tag{37}$$

$$\mathtt{1}^{\mathtt{o}} = 1^0 - \alpha a^0 \tag{38}$$

$$\mathtt{2}^{\mathtt{n},\sin} = -\mathtt{1}^{\mathtt{n},\sin} = b^{n,\sin} \tag{39}$$

$$\mathtt{2}^{\mathtt{n},\cos} = -\mathtt{1}^{\mathtt{n},\cos} = a^{n,\cos}. \tag{40}$$

or

$$2 = \mathtt{2}^{\mathtt{o}} + \alpha \sum_{n=0}^{+\infty} \sum_{m=-\infty}^{+\infty} \left\{\mathtt{2}^{\mathtt{n,m,sin}} \sin[(n\theta_1 + m\theta_2)x] + \mathtt{2}^{\mathtt{n,m,cos}} \cos[(n\theta_1 + m\theta_2)x]\right\} \tag{41}$$

$$1 = \mathtt{1}^{\mathtt{o}} - \alpha \sum_{n=0}^{+\infty} \sum_{m=-\infty}^{+\infty} \left\{\mathtt{2}^{\mathtt{n,m,sin}} \sin[(n\theta_1 + m\theta_2)x] + \mathtt{2}^{\mathtt{n,m,cos}} \cos[(n\theta_1 + m\theta_2)x]\right\} \tag{42}$$

where

$$\mathfrak{2}^{\mathfrak{o}} = 2^0 + \alpha a^0 \quad (43)$$

$$\mathfrak{1}^{\mathfrak{o}} = 1^0 - \alpha a^0 \quad (44)$$

$$\mathfrak{2}^{\mathfrak{n,m,sin}} = -\mathfrak{1}^{\mathfrak{n,m,sin}} = b^{n,m,\sin} \quad (45)$$

$$\mathfrak{2}^{\mathfrak{n,m,cos}} = -\mathfrak{1}^{\mathfrak{n,m,cos}} = a^{n,m,\cos}. \quad (46)$$

with $a^0 = a^{0,0,\cos}$.

1.2.3 Luminescence intensity

1.2.3.1 General expressions Reversibly photo-convertible luminophores are relevant for applying the HIOM and RIOM imaging protocols as long as the kinetic model given in Eq.(1) accounts for their dynamic behavior under illumination at an appropriate time scale. In section E, we successively examined fluorescent probes, phosphorescent probes, reversibly photo-convertible fluorophores, and the photosynthetic apparatus and showed that Eq.(1) does account for their dynamic behavior under illumination at an appropriate time scale. Moreover, we derived the relation linking the time evolution of the concentrations in their states $\mathsf{1}$ and $\mathsf{2}$ with the time evolution of their luminescence signal at the corresponding time scale. Two cases have to be considered:

- In the case of the phosphorescent probes, their luminescence signal is proportional to the concentration of the triplet state $\mathsf{2}$ and the time evolution of their phosphorescence intensity is governed by Eqs.(35,41);
- In the case of fluorescent probes, reversibly photo-convertible fluorophores, and the photosynthetic apparatus, the time evolution of their fluorescence signal involves terms which are products of concentrations and light intensity. Hence,we first defined in Eq.(47) the observable O_j associated to the observation at the wavelength λ_j with j = 1 or 2

$$O_j(t) = Q_{1,j}1(t) + Q_{2,j}2(t). \quad (47)$$

Then we subsequently extracted fluorescence emission $I_{\mathrm{F}}(t)$ from Eq.(48)

$$I_{\mathrm{F}}(t) = O_1(t)I_1(t) + O_2(t)I_2(t). \quad (48)$$

When the temporal dependence of $1(t)$ and $2(t)$ is given in Eqs.(35,36)

$$O_j(t) = \mathfrak{O}_{\mathrm{j}}^{\mathfrak{o}} + \sum_{n=1}^{\infty}\left[\mathfrak{O}_{\mathrm{j}}^{\mathrm{n,sin}}\sin(n\theta x) + \mathfrak{O}_{\mathrm{j}}^{\mathrm{n,cos}}\cos(n\theta x)\right]. \quad (49)$$

with

$$\mathfrak{O}_{\mathrm{j}}^{\mathfrak{o}} = Q_{1,j}\mathfrak{1}^{\mathfrak{o}} + Q_{2,j}\mathfrak{2}^{\mathfrak{o}} = Q_{1,j}1^0 + Q_{2,j}2^0 + (Q_{2,j} - Q_{1,j})\,\alpha a^0 \quad (50)$$

$$\mathfrak{O}_{\mathrm{j}}^{\mathrm{n,sin}} = (Q_{2,j} - Q_{1,j})\,\alpha b^{n,\sin} \quad (51)$$

$$\mathfrak{O}_{\mathrm{j}}^{\mathrm{n,cos}} = (Q_{2,j} - Q_{1,j})\,\alpha a^{n,\cos} \quad (52)$$

and

$$I_F(t) = \mathfrak{I}_{\mathfrak{F}}^{\mathfrak{o}} + \sum_{n=1}^{\infty} \left[\mathfrak{I}_{\mathfrak{F}}^{\mathfrak{n},\sin} \sin(n\theta x) + \mathfrak{I}_{\mathfrak{F}}^{\mathfrak{n},\cos} \cos(n\theta x) \right]. \tag{53}$$

In contrast to the expressions of the amplitudes of the $O_j(t)$ terms which are generic, the expressions of the amplitudes of the $I_{\mathrm{F}}(t)$ terms depend on the temporal dependence of the illumination.

When the temporal dependence of $1(t)$ and $2(t)$ is given in Eqs.(41,42)

$$O_j(t) = \sum_{n=0}^{+\infty} \sum_{m=-\infty}^{+\infty} \left\{ \mathfrak{O}_{\mathrm{j}}^{\mathfrak{n},\mathfrak{m},\mathfrak{sin}} \sin\left[(n\theta_1 + m\theta_2)\, x\right] + \mathfrak{O}_{\mathrm{j}}^{\mathfrak{n},\mathfrak{m},\mathfrak{cos}} \cos\left[(n\theta_1 + m\theta_2)\, x\right] \right\} \tag{54}$$

with

$$\mathfrak{O}_{\mathrm{j}}^{\mathfrak{o}} = \mathfrak{O}_{\mathrm{j}}^{\mathfrak{o},\mathfrak{o},\cos} = Q_{1,j} 1^0 + Q_{2,j} 2^0 + (Q_{2,j} - Q_{1,j})\, \alpha a^0 \tag{55}$$

$$\mathfrak{O}_{\mathrm{j}}^{\mathfrak{n},\mathfrak{m},\sin} = (Q_{2,j} - Q_{1,j})\, \alpha b^{n,m,\sin} \tag{56}$$

$$\mathfrak{O}_{\mathrm{j}}^{\mathfrak{n},\mathfrak{m},\cos} = (Q_{2,j} - Q_{1,j})\, \alpha a^{n,m,\cos} \tag{57}$$

and

$$I_F(t) = \sum_{n=0}^{+\infty} \sum_{m=-\infty}^{+\infty} \left\{ \mathfrak{I}_{\mathfrak{F}}^{\mathfrak{n},\mathfrak{m},\mathfrak{sin}} \sin\left[(n\theta_1 + m\theta_2)\, x\right] + \mathfrak{I}_{\mathfrak{F}}^{\mathfrak{n},\mathfrak{m},\mathfrak{cos}} \cos\left[(n\theta_1 + m\theta_2)\, x\right] \right\}. \tag{58}$$

Again, whereas the expressions of the amplitudes of the $O_j(t)$ terms are generic, the expressions of the amplitudes of the $I_{\mathrm{F}}(t)$ terms depend on the temporal dependence of the illumination.

1.2.3.2 Temporal dependences of the modulated illumination For both RIOM and HIOM imaging protocols, we considered two types of modulated illumination:

- Sinusoidal modulation:
  - for RIOM

$$I(t) = I_1^0 \left[1 + \alpha h_1(t)\right] + I_2^0 \left[1 + \alpha\delta h_2(t)\right] \tag{59}$$

$$h_1(t) = \sin(\omega t) \tag{60}$$

$$h_2(t) = \sin(\omega t + \varphi) \tag{61}$$

    with $\delta = 0$, or $\delta = 1$ with $\varphi = \pi$;
  - for HIOM

$$I(t) = I_1^0 \left[1 + \alpha h_1(t)\right] + I_2^0 \left[1 + \alpha h_2(t)\right] \tag{62}$$

$$h_1(t) = \sin(\omega_1 t) \tag{63}$$

$$h_2(t) = \sin(\omega_2 t + \varphi) \tag{64}$$

- Square-wave modulation, as a representative of more complex modulated illumination
  - for RIOM

$$I(t) = I_1^0\left[1+\alpha h_1(t)\right] + I_2^0\left[1+\alpha\delta h_2(t)\right] \quad (65)$$

$$h_1(t) = \frac{4}{\pi}\sum_{p=0}^{2}\frac{1}{2\times p+1}\sin[(2\times p+1)\theta x] \quad (66)$$

$$h_2(t) = \frac{4}{\pi}\sum_{p=0}^{2}\frac{1}{2\times p+1}\sin[(2\times p+1)\theta_2 x+\varphi] \quad (67)$$

with $\delta = 0$, or $\delta = 1$ with $\varphi = \pi$;

  - for HIOM

$$I(t) = I_1^0\left[1+\alpha h_1(t)\right] + I_2^0\left[1+\alpha\delta h_2(t)\right] \quad (68)$$

$$h_1(t) = \frac{4}{\pi}\sum_{p=0}^{2}\frac{1}{2\times p+1}\sin[(2\times p+1)\theta_1 x] \quad (69)$$

$$h_2(t) = \frac{4}{\pi}\sum_{p=0}^{2}\frac{1}{2\times p+1}\sin[(2\times p+1)\theta_2 x+\varphi] \quad (70)$$

with $\delta = 0$ or $\delta = 1$

1.2.3.3 Observables of RIOM and HIOM In relation to the preceding temporal dependences of the modulated illumination

- the RIOM observable S_R is the average value of the luminescence signal $S(t)$ (either global or from each analyzed pixel) from the reversibly photo-convertible luminophore over an integer number of periods of modulated illumination. In practice,

$$S_R = \frac{1}{kT}\int_0^{kT} S(t)dt; \quad (71)$$

- the HIOM observable S_H is the amplitude of either the in-phase (when $\varphi = \pi/2$ or $\varphi = -\pi/2$) or the quadrature-delayed (when $\varphi = 0$ or $\varphi = \pi$) component of the luminescence signal $S(t)$ (either global or from each analyzed pixel) from the reversibly photo-convertible luminophore modulated at $\omega_1 - \omega_2$ angular frequency. In this work, we retained the quadrature-delayed component of the luminescence signal with $\varphi = \pi$. In practice,

$$S_H = \frac{2}{kT}\int_0^{kT} S(t)\cos\left[(\omega_1-\omega_2)\,t\right]dt. \quad (72)$$

1.2.3.4 Resonance conditions of the observables of RIOM and HIOM To get non-vanishing H-OPIOM and RIOM signals, the parameters of the modulated illumination must be tuned in 2-3 orders of magnitude-wide ranges fixed by

matching the mean light intensity/ies and frequency/ies of the modulated illumination with simple functions of the rate constants associated with the luminophore photoactivation: the former must generate balanced proportions of the ground and photoactivated states whereas the latter must be in the range of the inverse of the photoactivation time.

Hence, as an outcome of the extensive investigation performed in the Appendix, we could evidence resonance conditions, which optimize the amplitude of the RIOM and HIOM observables as a function of the control parameters of the modulated illumination driving the values of the terms (K_{12}^0,θ) for RIOM, and (K_{12}^0,θ_1,θ_2) for HIOM (see Tables S1 and S2).

Table S1: Resonance conditions of the RIOM observable.

**Luminophore**	**Modulated illumination**	δ	φ	**log$_{10}$**K_{12}^0 **range**	**log$_{10}$**$K_{12}^{0,\max}$	**log$_{10}$**θ
Phosphorophores	Sine-wave	0	–	[-0.5;2.5]	0.72	≤ 0
Fluorophores	Sine-wave	0	–	[-2;1]	-0.20	≤ 0
Fluorophores	Sine-wave	1	π	[-2;0]	-0.66	≤ 0
Phosphorophores	Square-wave	0	–	[-1;2]	0.54	≤ 0
Fluorophores	Square-wave	0	–	[-2;1]	-0.21	≤ 0
Fluorophores	Square-wave	1	π	[-2;1]	-0.56	≤ 0

Table S2: Resonance conditions of the HIOM observable.

**Luminophore**	**Modulated illumination**	δ	φ	**log$_{10}$**K_{12}^0 **range**	**log$_{10}$**$K_{12}^{0,\max}$	**log$_{10}$**θ
Phosphorophores	Sine-wave	1	π	[-1;2.5]	0.64	≤ 0
Fluorophores	Sine-wave	1	π	[-3;1]	-0.69	≤ 0
Phosphorophores	Square-wave	1	π	[-1;2]	0.54	≤ 0
Fluorophores	Square-wave	1	π	[-2;1]	-0.56	≤ 0

1.3 Extraction of the characteristic time associated with the photo-conversion

The amplitudes of the time varying terms contained in Eqs.(49,53) and Eqs.(54,58) depend on θ, and θ_1 and θ_2 respectively. Hence, the investigation of the dependence of the observables of RIOM and HIOM on the applied angular frequency/ies is prone to yield the characteristic time τ_{12}^0. However, this retrieval necessitates to identify appropriate fitting functions.

Once the time dependence of the applied modulated illumination is known, one can introduce it into the master differential equation given in Eq.(21) and retrieve analytic expressions of the time varying terms contained in Eqs.(49,53) and Eqs.(54,58) upon truncating the Fourier expansion (30) or (32) at any order. In principle, the highest the order, the better the fitting function. However, the increase in the truncation order increases as well the number of fitting parameters.

In the Appendix, we have considered truncation at first and second order for retrieving the fitting function sought for, which ensured both a satisfactory fit over a range of angular frequencies delivering significant values of the RIOM and HIOM signals while limiting the number of fitted parameters and thereby providing robustness of the fit. It resulted in

two fitting functions to retrieve the characteristic time τ_{12}^0 from the dependence of the observables of RIOM and HIOM on the applied angular frequency/ies:

- In RIOM, we adopted the three floating parameter fitting function given in Eq.(73)

$$\mathcal{L}(\omega, p_1, p_2, p_3) = \frac{p_1(1 + 4(\omega\tau_{12}^0)^2)}{p_2 + 5(\omega\tau_{12}^0)^2 + 4(\omega\tau_{12}^0)^4} + p_3; \tag{73}$$

- In HIOM, we adopted the three floating parameter fitting function given in Eq.(74)

$$\mathcal{L}(\omega, p_1, p_2, p_3) = \frac{p_1(2 + 3(\gamma\omega\tau_{12}^0) + 3(\gamma\omega\tau_{12}^0)^2)}{p_2 + 8(\gamma\omega\tau_{12}^0) + 12(\gamma\omega\tau_{12}^0)^2 + 8(\gamma\omega\tau_{12}^0)^3 + 4(\gamma\omega\tau_{12}^0)^4} + p_3 \tag{74}$$

  where $\gamma = 1$ and $\gamma = 1.85$ with sine-wave and square-wave modulated illumination respectively.

The errors on the extracted luminescence lifetime provided in the Main Text and in the Supporting Information refer to errors given by the fits.

1.4 Implementation of the RIOM and HIOM protocols for luminescence lifetime imaging

1.4.1 Luminophores

The RIOM and HIOM protocols can be applied to retrieve maps of luminescence lifetime or kinetic fingerprints from any reversibly photoactivable luminophore understood in the most general sense. Hence, illumination should promote the luminophore to a photoactivated state from which it can relax back to the initial unactivated state either by thermal relaxation or by another illumination. Several examples of reversibly photoactivable luminophores are given in Section E and in the Main Text.

1.4.2 Equipments

In this manuscript, the RIOM and HIOM protocols have been implemented for luminescence lifetime imaging by using an epifluorescence microscope and a fluorescence macroscope. However, they can be as well implemented with alternative light detectors than cameras (e.g. photomultiplyers, MPPC, etc) when one is only interested to extract luminescence kinetic information at low frequency of luminescence detection without any requirement for imaging.

Key is here to be able to modulate the intensity of the excitation light at one frequency (with RIOM) or two frequencies (with HIOM) over a frequency window covering a 3 to 4 orders of magnitude-wide range around the inverse of the expected value of the luminescence lifetime to be mapped. When modulating illumination at two frequencies for HIOM, we noticed that using two independent light sources individually modulated at one frequency only is more favorable than having one light source modulated at two frequencies. Indeed, in the latter case, non-linearities arising at the current generator may contribute to degrade the HIOM signal.

The light modulation has to be either sinusoidal or square-wave if the goal is then to retrieve the lifetime by fitting with Eqs. (73–74). However, any type of periodic light modulation is presumably relevant for extracting kinetic fingerprints.

1.4.3 Procedure

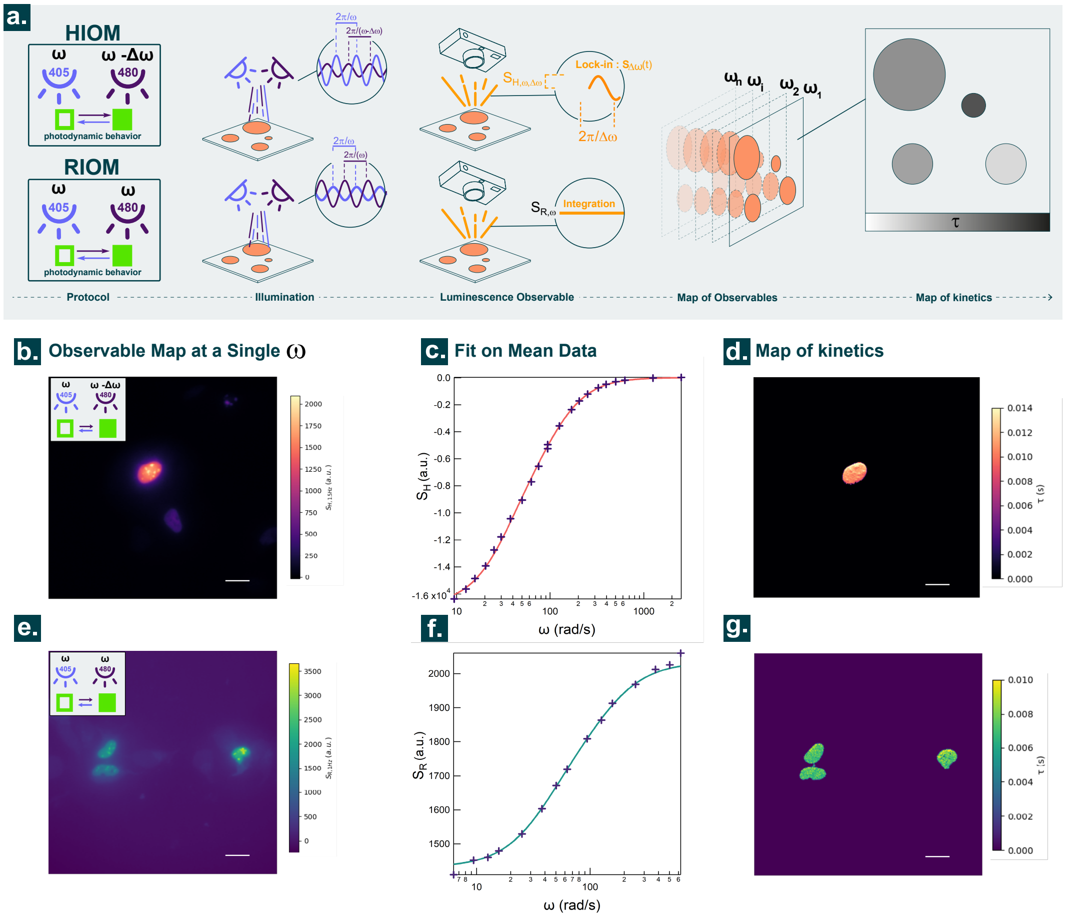

Figure S1: *Generic procedure for implementing the RIOM and HIOM protocols for luminescence lifetime imaging.* **a**: Flow of the HIOM and RIOM protocols. Modulated illumination involving one or two light sources photo-activates a luminophore, which deactivates via a light-driven pathway or thermal relaxation. In HIOM, the observable is the amplitude S_H of the quadrature-projected component of the luminescence modulated at low angular frequency $\Delta\omega$, which is imaged at low acquisition frequency under illumination modulated at two high angular frequencies ω and $\omega - \Delta\omega$. The map of luminescence lifetime τ is retrieved from exploiting the S_H dependence on ω observed at the constant difference of angular frequency $\Delta\omega$. In RIOM, the observable is the average luminescence S_R, which is imaged at low acquisition frequency under illumination modulated at high angular frequency ω. The map of luminescence lifetime τ is retrieved from exploiting the dependence of S_R on ω; **b**–**g**: The images of the HIOM S_H (**b**) and RIOM S_R (**e**) observables of the luminophore (here Dronpa-2) collected at the wavelength emission are acquired under periodically modulated illumination in a 3 to 4 orders of magnitude-wide range of frequency around the inverse of the expected value of the luminescence lifetime to be mapped. The ω -dependence of the HIOM (**c**) and RIOM (**f**) observables provides a kinetic fingerprint of the luminophore. When the luminophore is engaged in a two-state photodynamic model, the ω-dependence of the HIOM and RIOM observables can be further fitted with Eqs. (74) and (73) respectively, in order to yield HIOM (**d**) and RIOM (**g**) image of the luminescence photoactivation time of the luminophore.

1.4.3.1 Generic HIOM protocol (Figure S1)

1. Have a guess about the orders of magnitude of the photoactivation cross sections $\sigma_{12,1}$, $\sigma_{12,2}$, $\sigma_{21,1}$, $\sigma_{21,2}$, and of

the thermal rate constant k_{21}^{Δ}, which characterize the photochemical and thermal thermokinetic parameters of the photoactivation/deactivation process described in Eq.(1) for the targeted luminophore;[1]

2. Fix the mean intensities of the excitation lights, I_1^0 and I_2^0,[2,3] to the resonance condition, which makes the exchange constant K_{12}^0 defined in Eq.(18) to adopt the value given in Table S2. If your setup cannot reach the optimal values of light intensity,[4] be at the closest to the resonance condition in order to benefit from the highest values of the HIOM signal;

3. Once the light intensities I_1^0 and I_2^0 have been fixed, compute the photoactivation time τ_{12}^0 defined in Eq.(16);

4. Define the range of angular frequencies $[\omega_{\min}; \omega_{\max}]$ to be spanned.[5] The central value of the range is defined as $\omega_{central} = 1/\tau_{12}^0$ and the range has ideally to extend at least two orders of magnitude away from $\omega_{central}$ on both its sides;

5. Define the value of the angular frequency $\Delta\omega$ at which extraction of the HIOM observable S_H will be performed. It should be lower than half the angular frequency of acquisition of the camera $\omega_{camera} = 2\pi f_{camera}$;

6. Record a movie of luminophore luminescence image $S(x, y, t)$ or a time series of luminophore luminescence signal $S(t)$ over an integer number k of the period $T = 2\pi/\omega$ of the modulated illumination upon applying dual modulation of illumination at an excitation wavelength in the most red-shifted absorption band of the luminophore at angular frequencies ω and $\omega - \Delta\omega$ for a range of ω values regularly spanning the $[\omega_{\min}; \omega_{\max}]$ range in logarithmic scale;

7. Process the data to generate the HIOM observable S_H at each investigated value of ω by exploiting Eq.(72) where $\omega_1 - \omega_2 = \Delta\omega$;

[1]The photoactivation cross sections are the product of the cross section for light absorption – measuring the effective molecular surface for light absorption – by the quantum yield of photoactivation, which measures the probability that photoactivation occurs after light absorption. The cross section for light absorption is linked to the molar absorption coefficient ε by Eq.(75)

$$\sigma(\lambda_{\text{exc}})\left(\text{m}^2.\text{mol}^{-1}\right) = 0.1 \times \varepsilon(\lambda_{\text{exc}})\left(\text{mol.L}^{-1}.\text{cm}^{-1}\right) \tag{75}$$

[2]Note that I_2^0 is vanishing when the back reaction shown in Eq.(1) is not driven by light (case of thermal relaxation) or that only one light drives both the forward and backward reaction (1).

[3]In this manuscript, we provide the values of the light intensities in E.m^{-2}.s^{-1} (or mol. of photons.m^{-2}.s^{-1}). This unit is currently used in actinometry. However, it is not often used in other fields such as optical microscopy, in which the researchers prefer to adopt W.m^{-2}. We provide below the conversion between both units. We consider a monochromatic light of wavelength λ_{exc}. Its values in E.m^{-2}.s^{-1} and W.m^{-2} are respectively denoted as $I(\lambda_{\text{exc}}, \text{E.m}^{-2}.\text{s}^{-1})$ and $I(\lambda_{\text{exc}}, \text{W.m}^{-2})$. The relation between $I(\lambda_{\text{exc}}, \text{E.m}^{-2}.\text{s}^{-1})$ and $I(\lambda_{\text{exc}}, \text{W.m}^{-2})$ is given in Eq.(76)

$$I(\lambda_{\text{exc}}, \text{W.m}^{-2}) = \frac{hcN_A}{\lambda_{\text{exc}}} \times I(\lambda_{\text{exc}}, \text{E.m}^{-2}.\text{s}^{-1}) \approx 0.12 \times \frac{\text{I}(\lambda_{\text{exc}}, \text{E.m}^{-2}.\text{s}^{-1})}{\lambda_{\text{exc}}\,(\text{m})} \tag{76}$$

with the Planck constant $h = 6.63\ 10^{-34}$ m^2.kg.s^{-1}, speed of light in a vacuum $c = 3.00\ 10^8$ m.s^{-1}, the Avogadro number $N_A = 6.02\ 10^{23}$ mol^{-1}, and where λ_{exc} is in m.

[4]Protocols to measure light intensity are provided in reference.[1]

[5]The angular frequency ω and the frequency f are linked by the relation: $\omega = 2\pi f$.

8. Plot the dependence of $S_H(\omega)$, which provides the HIOM kinetic fingerprint of the luminophore sought for;

9. If the photoactivation of the luminophore obeys the two-state photodynamic model shown in Eq.(1), it is further possible to retrieve the photoactivation time of the luminophore, τ_{12}^0, from the preceding kinetic fingerprint by exploiting the fitting function given in Eq.(74).

1.4.3.2 Generic RIOM protocol (Figure S1)

1. Have a guess about the orders of magnitude of the photoactivation cross sections $\sigma_{12,1}$, $\sigma_{12,2}$, $\sigma_{21,1}$, $\sigma_{21,2}$, and of the thermal rate constant k_{21}^{Δ}, which characterize the photochemical and thermal thermokinetic parameters of the photoactivation/deactivation process described in Eq.(1) for the targeted luminophore;

2. Fix the mean intensities of the excitation lights, I_1^0 and I_2^0, to the resonance condition, which makes the exchange constant K_{12}^0 defined in Eq.(18) to adopt the value given in Table S1. If your setup cannot reach the optimal values of light intensity, be at the closest to the resonance condition in order to benefit from the highest values of the RIOM signal;

3. Once the light intensities I_1^0 and I_2^0 have been fixed, compute the photoactivation time τ_{12}^0 defined in Eq.(16);

4. Define the range of angular frequencies $[\omega_{\min}; \omega_{\max}]$ to be spanned. The central value of the range is defined as $\omega_{central} = 1/\tau_{12}^0$ and the range has ideally to extend at least two orders of magnitude away from $\omega_{central}$ on both its sides;

5. Record a movie of luminophore luminescence image $S(x, y, t)$ or a time series of luminophore luminescence signal $S(t)$ over an integer number k of the period $T = 2\pi/\omega$ of the modulated illumination upon applying dual modulation of illumination at an excitation wavelength in the most red-shifted absorption band of the luminophore at angular frequency ω for a range of ω values regularly spanning the $[\omega_{\min}; \omega_{\max}]$ range in logarithmic scale;

6. Process the data to generate the RIOM observable S_R at each investigated value of ω by exploiting Eq.(71);

7. Plot the dependence of $S_R(\omega)$, which provides the RIOM kinetic fingerprint of the luminophore sought for;

8. If the photoactivation of the luminophore obeys the two-state photodynamic model shown in Eq.(1), it is further possible to retrieve the photoactivation time of the luminophore, τ_{12}^0, from the preceding kinetic fingerprint by exploiting the fitting function given in Eq.(73).

2 Materials and methods

2.1 Instruments

2.1.1 Epifluorescence microscope

A diagram of the components of the home-built inverted epifluorescence microscope is shown in Figure S2.

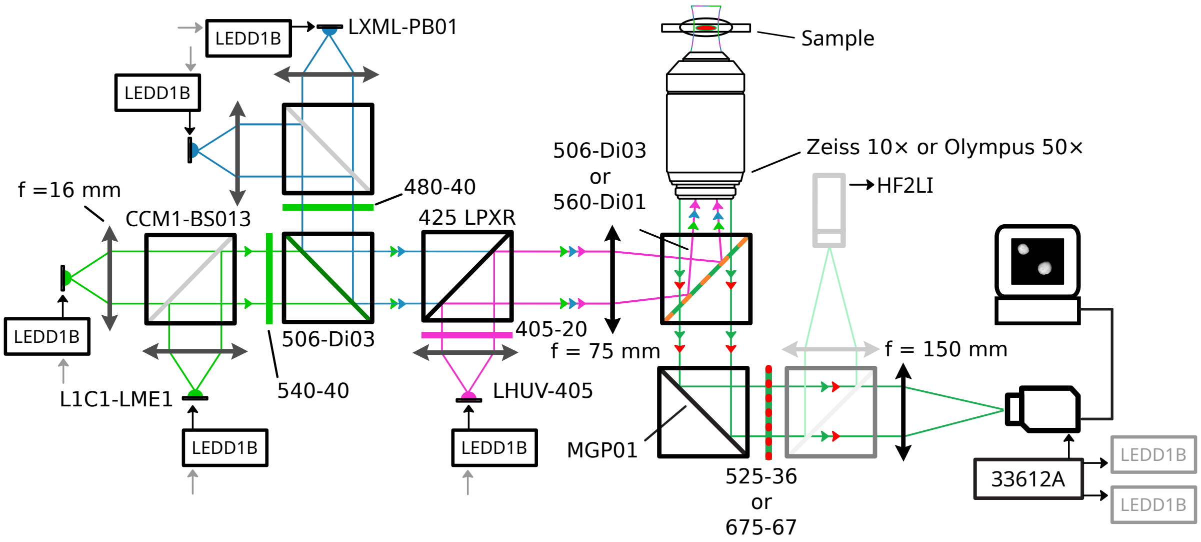

Figure S2: *Diagram of the components of the home-built fluorescence microscope.*

2.1.2 Fluorescence macroimager

An image of the setup, and a diagram of its optical components, shown in the form of a computer rendered CAD model, created in Rhinoceros 3D (Robert McNeel & Associates, Seattle, WA, US), are shown in Figure S3.

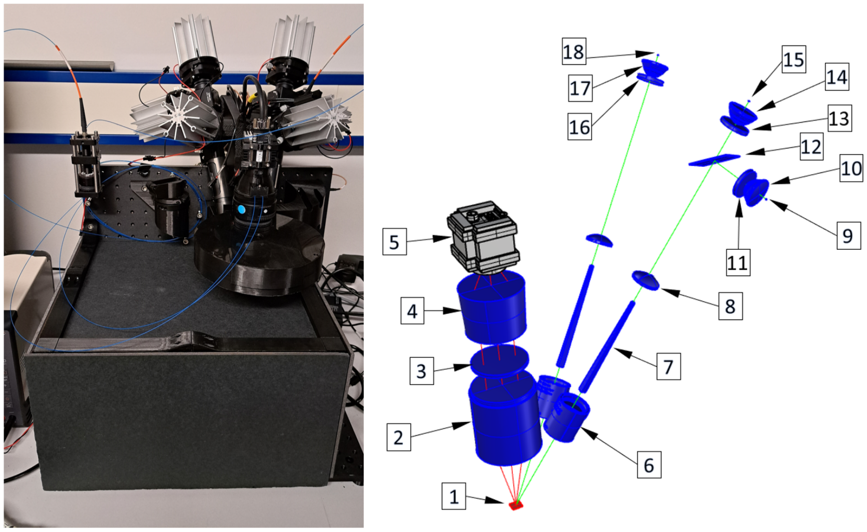

Figure S3: *Image of the home built fluorescence macroimager (left) and 3D CAD rendered diagram of its optical elements (right).* 1: Sample; 2: Microscope Objective; 3: Emission Filter; 4: Camera Objective ($f = 50$ mm); 5: Camera; 6: Matched Achromatic Doublets; 7: Tapered Lightpipes; 8: Planoconvex Lens; 9: 405 nm LED; 10: Condenser; 11: Filter; 12: Dichroic Mirror; 13: Filter; 14: Condenser; 15: 470 nm LED; 16: Excitation Filter; 17: Condenser; 18: 405 nm LED. The LEDs present in this Figure are for the experiments with the Dronpa-2 solution.

2.1.3 Measurement of light intensity

The light intensities on the imaging setups were measured either through exploiting fluorescent actinometers[1] or by using a power meter.

2.1.3.1 Epifluorescence microscope The camera in this setup could not reach a high enough frequency, when using the full field of view, to be able to video the decay of the fluorescent actinometer at the light intensities involved, and therefore obtain the map of light intensity over the entire field of view. In light of this, the field of view was reduced until it was possible to achieve a frame rate high enough to record the fluorescence decay. Hence, the mean light intensity applied at 470 and 405 nm in the experiments on fixed cells labeled at the nucleus with the fluorescent protein Dronpa-2 was directly measured from analyzing the time evolution of the Dronpa-2 fluorescence recorded at 196 Hz over area of 64×64 pixels^2 upon turning on constant 470 nm light and then turning on constant 405 nm light over over the 470 nm background.

The mean light intensity applied at 405 nm in the experiment on phosphorescent labelled beads was measured with the same protocol but by recording the time evolution of the fluorescence of a 32.5 μM Dronpa-2 solution. Once the light intensity at 470 nm was known for all the experiments conditions, the light intensity at 540 nm was measured according to the protocol described in[1] by recording the fluorescence emission of a 9 μM solution of 7-Hydroxy-9H-(1,3-dichloro-9,9-dimethylacridin-2-one) acting as a light intensity transferring fluorophore under excitation at 470 nm and at 540 nm.

2.1.3.2 Fluorescence macroimager In order to set the minimum and maximum voltage levels to be sent to the LED driver, or LEDs directly (in the case of the experiments on Arabidopsis), to achieve the desired minumum, maximum and therefore average light intensity values of the sinusoidally modulated light from the LEDs, a power meter (S170C and PM100A,Thorlabs Inc., Newton, NJ, US) coupled with a measurement of the illumination area was used to measure the light intensity. The area was determined by imaging the illumination coverage at the sample plane with a steel ruler next to it for scale, and performing the measurement within image analysis software. Since this method is only valid in the case of the illumination being uniform, the uniformity was evalutated using the procedure referenced above, with the fluorescent actinometer Dronpa-2. The illumination was found to be highly uniform, with a deviation not exceeding 10%.

2.1.4 Generation of harmonics-free sine wave modulated illumination

Since LEDs generate heat, and that heat causes their output light intensity to decrease, a purely sinusoidal electric drive signal results in a nonpure sinusoidal illumination response, worsening with higher drive currents.

In the case of the experiment with Dronpa-2 in the microfluidic device, imaged with the macroscope, the LEDs needed to be driven with high currents, and therefore it was pertinent to correct for the deviation from a pure sinusoidal modulation. Regarding the protocol, it was run ahead of time, generating waveforms which when sent to the LEDs, would result in a purely sinusoidal illumination profile. The protocol involved the recording of the oscillating illumination light using a silicon photomultiplier (SiPM) module (PE3315-WL-TIA-SP, Ketek GmbH, Munich, Germany) coupled to the DAQ card. The frequency content, along with corresponding phase information, of the signal is recovered from Fourier Transform, and for the undesired frequency components, added to the original signal in antiphase.[2] In this way, the unwanted frequencies can be cancelled out.

Such a correction protocol was not implemented for the other experiments performed either with the microscope or with the macroscope on Arabidopsis plants.[6]

2.1.5 Acquisition protocols

2.1.5.1 Dronpa-2 labeled cells under sine-wave modulated illumination Frame series of each acquisition under the microscope were post-processed under Python with the numpy and pandas libraries. A threshold was determined with the Otsu algorithm. A mask was then applied on each frame based on this threshold to determine the region of interest (ROI). Background was defined as everything out of the ROI. Background contribution was averaged outside the ROI and then retrieved from the signal in the ROI.

2.1.5.2 Dronpa-2 Filled Microfluidic Chambers under sinusoidal modulated illumination In this experiment, each chamber of the PDMS microfluidic device was filled with a 32.5 μM Dronpa-2 solution in PBS (Phosphate Buffered Saline, 0.01 M, [NaCl 0.138 M; KCl 0.0027 M], pH 7.4) containing BSA (Bovine Serum Albumin, 50 mg/mL).

[6] In the latter case, the light levels used were much lower and harmonic correction was not required.

In terms of illumination, for implementing the RIOM protocol, the 470 nm illumination was oscillated between practically 0 and near its maximum possible. This resulted in an average intensity level of 1.8 mE.m^{-2}.s^{-1}. The average level of the 405 nm illumination was then set to a level which resulted in a balanced forward and backward conversion rate. This resulted in an average level of mE.m^{-2}.s^{-1}. The frequencies explored here, in Hertz, were the following: [102.4, 51.2, 25.6, 12.8, 6.4, 3.2, 1.6, 0.8, 0.4, 0.2, 0.1, 0.05, 0.025, 0.0125]. In order to maximize the amplitude of modulation of the Dronpa-2 fluorescence, the two wavelengths were modulated in antiphase. In regard to imaging, the camera was operated at a frequency of 0.0167 Hz, with an exposure time of 20 s, for a duration of 240 s, where the first frame was triggered with a delay of 20 s. The excitation filter used in this case was a 540/50 nm bandpass filter (86-366, Edmund Optics Inc., Barrington, NJ, US). Following measurement, the frames corresponding to each frequency were averaged, resulting in one RIOM image per frequency.

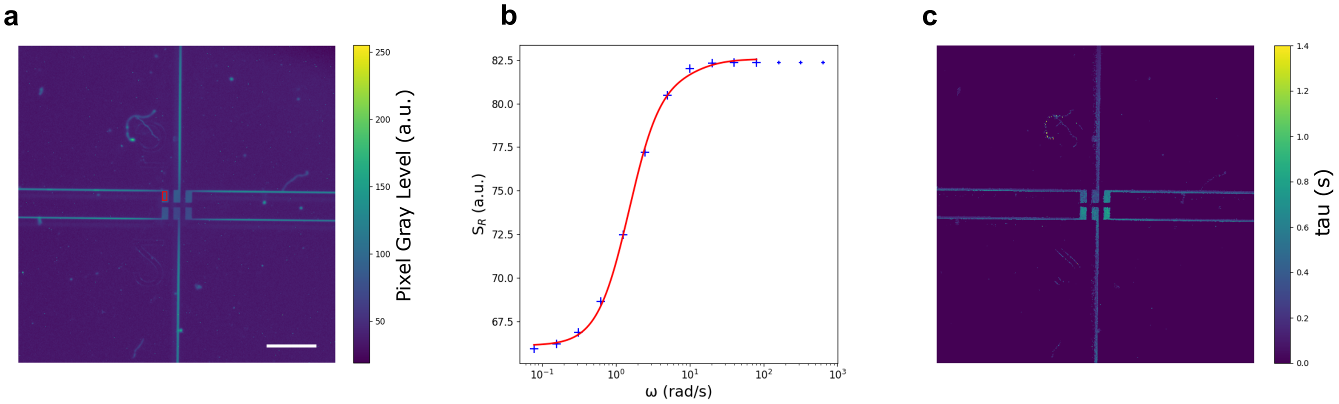

Figure S4: *RIOM for wide-field imaging of the Dronpa-2 photoswitching time under sinusoidally modulated illumination in macrofluorescence imaging.* **a**: 12.5 mHz acquired image of the RIOM signal emitted at 515 nm from a 32.5 μM Dronpa-2 solution in PBS (Phosphate Buffered Saline: 0.01 M, NaCl: 0.138 M; KCl: 0.0027 M, pH 7.4) containing BSA (Bovine Serum Albumin, 50 mg/mL) under sinusoidally modulated illumination at 470 nm ($I_1 = 1.8$ mE.m^{-2}.s^{-1}, 4.7 10^{-2} W.cm^{-2}; $\omega_1 = 643$ rad.s^{-1}, $f_1 = 102$ Hz) and antiphase sinusoidally modulated light at 405 nm ($I_2 = 3.6$ mE.m^{-2}.s^{-1}, 1.1 10^{-1} W.cm^{-2}; $\omega_2 = 643$ rad.s^{-1}, $f_2 = 102$ Hz). See Table S4; **b**: ω_1-Dependence of the Dronpa-2 RIOM signal emitted from the chamber frame in red in **a**. Large markers: Experimental points included in fitting procedure, Small markers: Experimental points not included in fitting procedure, Solid line: Fit with Eq.(73). See Table S7; **c**: RIOM Image of the Dronpa-2 fluorescence photoswitching time. Scale bar: 1 mm.

2.1.5.3 Droplets of Dronpa-2 solution at various concentrations under sinusoidal modulated illumination

Sample Preparation Purified Dronpa-2 stock solution (130 μM; in DPBS [pH = 7.4]) was diluted with PBS (pH = 7.4, 50 mM sodium phosphate, 150 mM NaCl) to generate a series of Dronpa-2 solutions having concentrations of 4, 2, 1, 0.5, 0.25, 0.125, 0.0625, 0.03125 and 0.015625 μM. Then, 0.5 μL aliquots of each solution were deposited on a circular glass coverslip, resulting in the arrangement shown in Figure S5a. Immediately after, a second circular glass coverslip was placed on top, with a spacer (Gene Frames AB0578; Thermo Scientific Inc., Waltham, MA, US) separating them.

Acquisition Protocol The protocol was applied using the macroscope system. In this case, the illumination area was reduced by a factor of 4 by inverting the lightpipes of the illumination system, and changing the associated light injection lens to one of a longer focal length (LA1027-A, Thorlabs Inc., Newton, NJ, US). In doing so, the maximum achievable light intensities were increased by a factor of 4, thus the LEDs could be operated at a lower power to achieve the desired intensities. As a result, the generation of undesired frequency components due to heating was minimal, meaning that harmonic correction to obtain clean sinusoidal signals was not required.

The average level of the 470 nm illumination, which was sinusoidally oscillated around this average value, was set to be 2.8 mE.m^{-2}.s^{-1}, while that for the 405 nm illumination was 0.9 mE.m^{-2}.s^{-1}, which was also oscillated around its average value but in antiphase with the 470 nm signal. The two average levels give an overall time constant of 1.05 s. The experiment was conducted by sinusoidally oscillating the two illumination lights, in antiphase as mentioned, over a range of frequencies in the range [0.01:2] Hz, in a sequential manner. In regard to fluorescence signal collection, the macroscope's camera was operated at a frequency giving 20 sample points per sinusoid period, with an exposure time of 20 ms and a gain of 0. The frequencies explored, and number of periods used for each, were [f;No. Periods]: [0.01;5], [0.02;5], [0.04;5], [0.08;10], [0.1;10], [0.2;10], [0.4;10], [0.8;20], [1;20], [2;40]. The experiment was run three times.

Image Processing and Results The data acquired in this series of experiments enable us to retrieve not only the RIOM signal, but also the Speed OPIOM signal, which is here used as a reference imaging protocol with established high sensitivity.[3]

Processing began with the selection of an ROI for each sample blob within the image (Figure S5b). Following this, the mean pixel intensity over each ROI was extracted over all frames, and for each frequency, generating a signal for each sample blob for each frequency. In the calculation of the mean over the ROIs, values below 10 and exceeding 4000 were excluded. Then, for each frequency, and for each ROI, the mean and also the out-of-phase frequency component of the last 3 sinusoid periods were calculated. The dependance of these with frequency for each ROI and each of the 3 experiment runs is shown in Figure S5e. The RIOM amplitude (i.e., the RIOM signal at the highest frequency minus that at the lowest frequency) and out-of-phase signal at 0.1 Hz, corresponding to approximately the Speed-OPIOM resonance frequency, is shown as a function of concentration in Figure S5c.

These curves first confirm that RIOM is as sensitive as Speed OPIOM to retrieve information on the concentration of the Dronpa-2 solution: the measured concentration decreases exponentially as was desired down to the 0.1 μM range with both imaging protocols. Moreover, the angular frequency-dependence of the RIOM and Speed OPIOM signals was fitted in order to extract the Dronpa-2 photoswitching time for each case (Figure S5d).[7] In line with the concentration

[7]Eq.(73) has been used to fit the RIOM data. Speed OPIOM data have been fitted with Eq.(77)[4]

$$\mathcal{L}(\omega, p_1, p_2) = p_1 \frac{(\omega\tau_{12}^0)}{1 + (\omega\tau_{12}^0)^2} + p_2. \tag{77}$$

range reliably delivering the RIOM and Speed OPIOM observables, we observed that the photoswitching time can be satisfactorily obtained down to concentrations of the 0.1 μM range. It should be here noted, however, that this concentration range is indicative since it is pertinent only for this luminophore, instrument and acquisition settings.

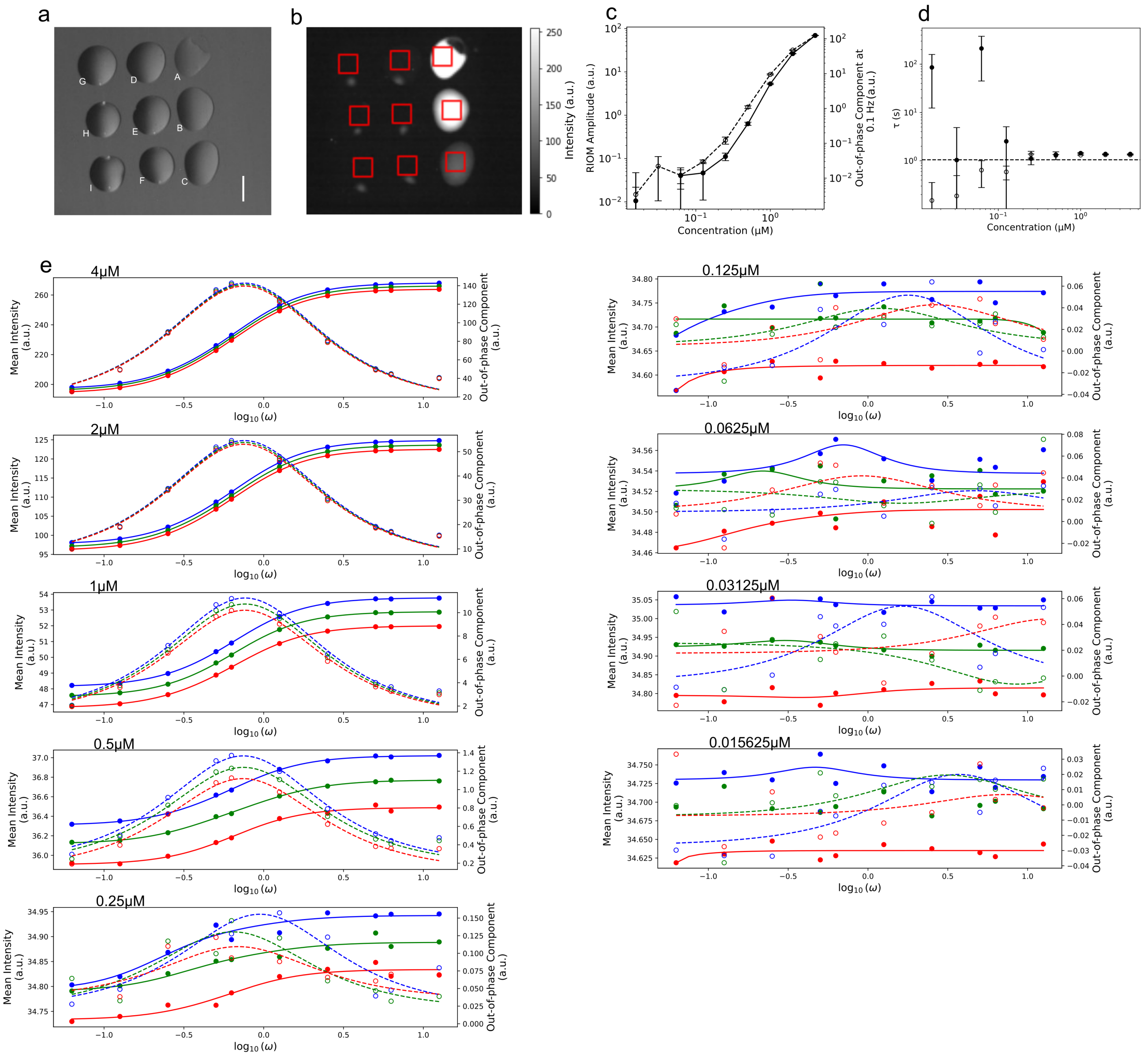

Figure S5: *RIOM protocol sensitivity when applied with the macroscope imaging system with droplets of solution at decreasing Dronpa-2 concentration.* **a**: Image of the deposited droplets of Dronpa-2 solution, taken with a side-on white light illumination, at concentrations: 4, 2, 1, 0.5, 0.25, 0.125, 0.0625, 0.03125 and 0.015625 μM; Scale bar: 1 mm; **b**: Normalized image frame with ROIs marked corresponding to the droplets at different concentrations; e: Dependance of the RIOM signal (disks) and out-of-phase Speed OPIOM component (circles), determined on the final 3 oscillation periods, as a function of angular frequency; and applied fits [Eqs.(73) and (77)] for extraction of the time constant, for each Dronpa-2 solution concentration. Experiment 1 (blue), 2 (green) and 3 (red); fitted curve on the RIOM (solid lines) and Out-of-phase Speed OPIOM signals (dotted lines); **c**: Out-of-phase Speed OPIOM signal level at 0.1 Hz (circles), and RIOM amplitude (disks), as a function of concentration; **d**: Extracted time constant at each concentration with RIOM (disks) and from the Out-of-phase Speed OPIOM signal (circles); dotted line: expected time constant.

2.1.5.4 Dronpa-2 labeled cells under square-wave modulated illumination We evaluated the HIOM and RIOM protocols under square-wave modulated illumination with the epifluorescence microscope. We first recorded images of

fixed cells labeled at the nucleus with the fluorescent protein Dronpa-2 emitting at 525 nm at 12 Hz acquisition frequency upon square-wave modulating the 480 and 405 nm lights in antiphase around 0.10 and 0.16 E.m^{-2}.s^{-1} (2.6 and 4.9 W.cm^{-2}) average light intensities with 84% duty cycle at frequencies departing from each other by 0.5 Hz in the [1 Hz; 100 Hz] range (Figure S6a).

Frame series of each acquisition under the microscope were post-processed under Python with the numpy and pandas libraries. A threshold was determined with the Otsu algorithm. A mask was then applied on each frame based on this threshold to determine the region of interest (ROI). Background was defined as everything out of the ROI. Background contribution was averaged outside the ROI and then retrieved from the signal in the ROI.

After satisfactorily fitting the dependence of the HIOM signal modulated at 0.5 Hz at each pixel on the angular frequency of the 480 nm light modulation (Figure S6b), we retrieved an HIOM image of the Dronpa-2 photoswitching time τ (Figure S6c), which proved consistent with the expectation: we found $\langle\tau\rangle = 8.8 \pm 0.4$ ms over the nucleus whereas $\langle\tau\rangle = 11.4 \pm 0.2$ ms was anticipated from exploiting the mean light intensities and the photoswitching information at 480 and 405 nm for Dronpa-2.[1],[5] We then evaluated RIOM by imaging the same sample of Dronpa-2 labeled fixed cells at 12 Hz acquisition frequency upon square-wave modulating the 480 and 405 nm lights in antiphase around 0.10 and 0.16 E.m^{-2}.s^{-1} (2.6 and 4.9 W.cm^{-2}) average light intensities with 84% duty cycle in the [1 Hz; 100 Hz] range (Figure S6d). After fitting the dependence of the RIOM signal on the angular frequency of the modulated illumination at each pixel (Figure S6e), we retrieved the RIOM image of the Dronpa-2 photoswitching time (Figure S6f): we found $\langle\tau\rangle = 14.4 \pm 0.2$ ms over the nucleus again in line with $\langle\tau\rangle = 11.4 \pm 0.2$ ms computed from the light intensities and the Dronpa-2 photoswitching information.[1],[5]

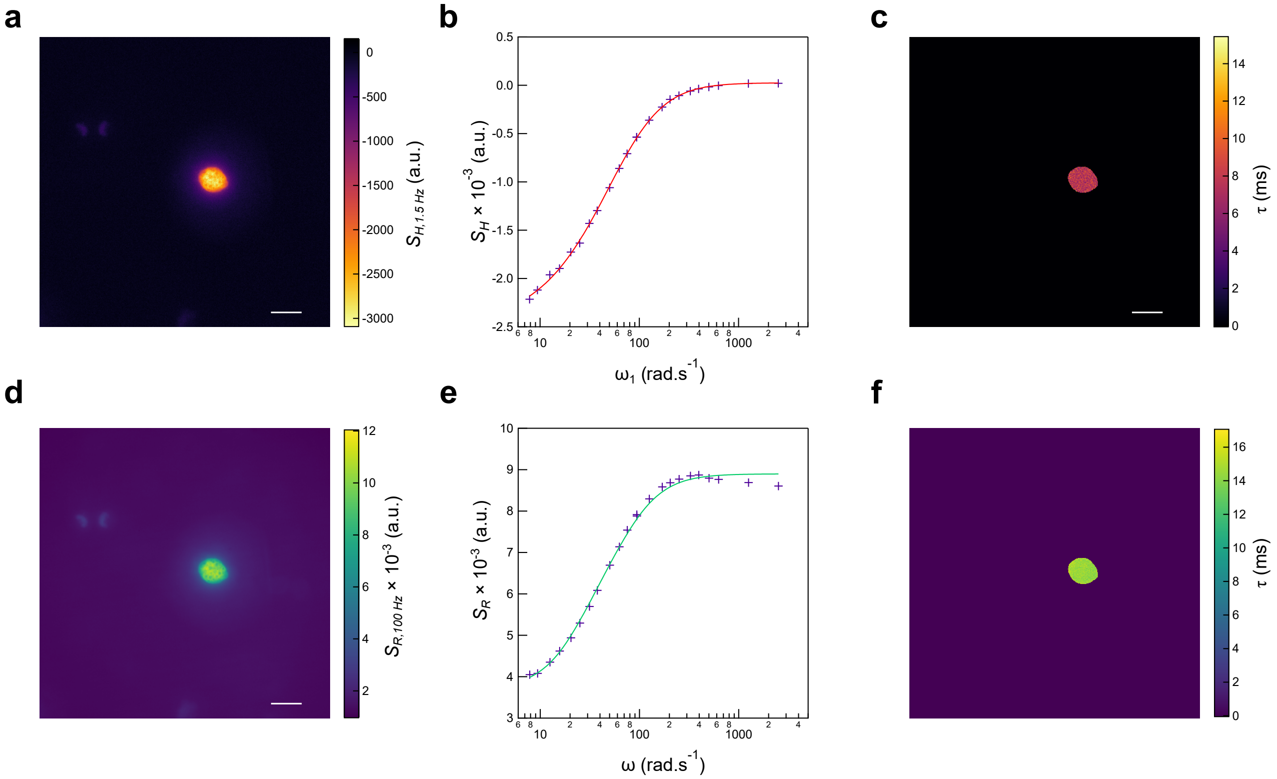

Figure S6: *Validation of HIOM and RIOM for wide-field imaging under square-wave modulated illumination.* **a**: 12 Hz acquired image of the HIOM signal emitted at 525 nm from a fixed H2B-Dronpa-2 expressing U2OS cell under dual antiphase square-wave modulated illumination at 480 ($I_1 = 0.10$ E.m^{-2}.s^{-1}, 2.6 W.cm^{-2}; $\omega_1 = 5\pi/2$ rad.s^{-1}, $f_1 = 1.25$ Hz) and 405 nm ($I_2 = 0.16$ E.m^{-2}.s^{-1}, 4.9 W.cm^{-2}; $\omega_2 = 7\pi/2$ rad.s^{-1}, $f_2 = 1.75$ Hz). See Table S4; **b**: ω_1-Dependence of the Dronpa-2 HIOM signal emitted from the cell nucleus at constant angular frequency $\Delta\omega = \omega_1 - \omega_2$. Markers: Experiments, Solid line: Fit with Eq.(74). See Table S6; **c**: RIOM Image of the Dronpa-2 fluorescence photoswitching time;**d**: 12 Hz acquired image of the RIOM signal emitted at 525 nm from a fixed H2B-Dronpa-2 expressing U2OS cell under dual antiphase square-wave modulated illumination at 480 ($I_1 = 0.10$ E.m^{-2}.s^{-1}, 2.6 W.cm^{-2}; $\omega_1 = 5\pi/2$ rad.s^{-1}, $f_1 = 1.25$ Hz) and 405 nm ($I_2 = 0.16$ E.m^{-2}.s^{-1}, 4.9 W.cm^{-2}; $\omega_2 = 7\pi/2$ rad.s^{-1}, $f_2 = 1.75$ Hz). See Table S4; **e**: ω_1-Dependence of the Dronpa-2 RIOM signal emitted from the cell nucleus. Markers: Experiments, Solid line: Fit with Eq.(73). See Table S7; **f**: RIOM Image of the Dronpa-2 fluorescence photoswitching time. Scale bar: 20 μm.

2.1.5.5 Luminescent metal complexes and dyes embedded in polystyrene beads The RIOM imaging experiments on monolayers of dye-loaded polystyrene beads were performed under sinusoidally modulated illumination at

- 405 nm with average intensity of:
  - 8 mE.m^{-2}.s^{-1} for 4.5μm Palladium-loaded beads (Figures 3a,b);
  - 30 mE.m^{-2}.s^{-1} for 4.5μm Platinum-loaded beads (Figures 3d,e,g);
  - 80 mE.m^{-2}.s^{-1} for 3, 6 and 10 μm Platinum-loaded beads (Figure 3h,i);
- 540 nm with average intensity 140 mE.m^{-2}.s^{-1} for the dependence of the normalized overall phosphorescence

signal at 675 nm over the whole image of PdOEP-(Figure 3c) and PtOEP-(Figure 3f) loaded 4.5μm diameter carboxylate polystyrene microbeads on the frequency of the modulated excitation light.

In each case, these light intensities were chosen by approaching the light intensity theoretically predicted at resonance for maximizing the RIOM signal while considering further experimental constraints such as photobleaching of the samples or limitation of the accessible LED light intensity when using high-frequency modulation.

For each modulation frequency, the camera acquired 12 frames with an acquisition frequency set at 12 Hz (exposure time was 0.0833 s for each frame for a total duration of 1 s for a modulation frequency). Over the 1 s of the duration of the acquisition, it has been made sure that there was an integer number of periods of the modulated signal. For each modulation frequency, triggering of the camera acquisition was synchronized with the start of the periodic excitation light (using the option "External start" in the Solis software, Andor Technology). Light was collected through a 675/67 nm bandpass filter (Semrock FF02-675/67-25). The explored frequency range of light modulation was $[100\text{ Hz}; 100\text{ kHz}]$ for the Platinum- and Palladium-loaded beads;

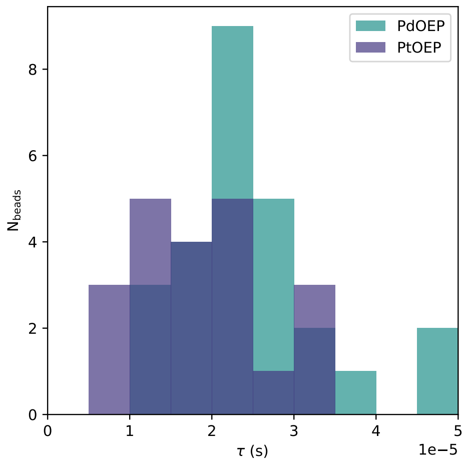

Figure S7: *RIOM wide-field image-retrieved distribution of the phosphorescence lifetime from metal complex-loaded carboxylate polystyrene microbeads under sinusoidally modulated 405 nm light in epifluorescence microscopy.* **a**: PtOEP-loaded 3 μm diameter carboxylate polystyrene microbeads ($n = 25$). $I = 30$ mE.m^{-2}.s^{-1}, 0.24 W.cm^{-2}; ω= 628 rad.s^{-1}, f = 100 Hz; **b**: PdOEP-loaded 4.5 μm diameter carboxylate polystyrene microbeads ($n = 25$). $I = 8$ mE.m^{-2}.s^{-1}, 0.91 W.cm^{-2}; ω= 628 rad.s^{-1}, f = 100 Hz. $\lambda_{em} = 675$ nm. See Table S4. The averaged lifetime measured for PtOEP-labeled beads is 19 µs and the standard deviation is 6 µs. The averaged lifetime measured for PdOEP-labeled beads is 26 µs and the standard deviation is 7 µs.

A binning of 4 was applied on each recorded frame at each frequency to enhance global convergence of the fitting process. Frame series of each acquisition under the microscope were post-processed under Python with the numpy and pandas libraries. A threshold was determined with the Isodata algorithm. A mask was then applied on each frame based on this threshold to determine the region of interest (ROI). Background is defined as everything out of the ROI. Background contribution is averaged outside the ROI and then retrieved from the signal in the ROI. The dependence of the RIOM

signal on the angular frequency of the modulated illumination was fitted at each pixel to deliver the RIOM image of the photoconversion time of the luminescent dye. Average values for each dye and size of particle can be found in Table S4.

For 3 μm Platinum-loaded and 4.5 μm Palladium-loaded beads, we further built an histogram of the photoconversion time obtained on 25 beads upon averaging the RIOM signal over a region of interest attributed manually to one bead in order to retrieve the mean value and standard deviation of the photoconversion times.

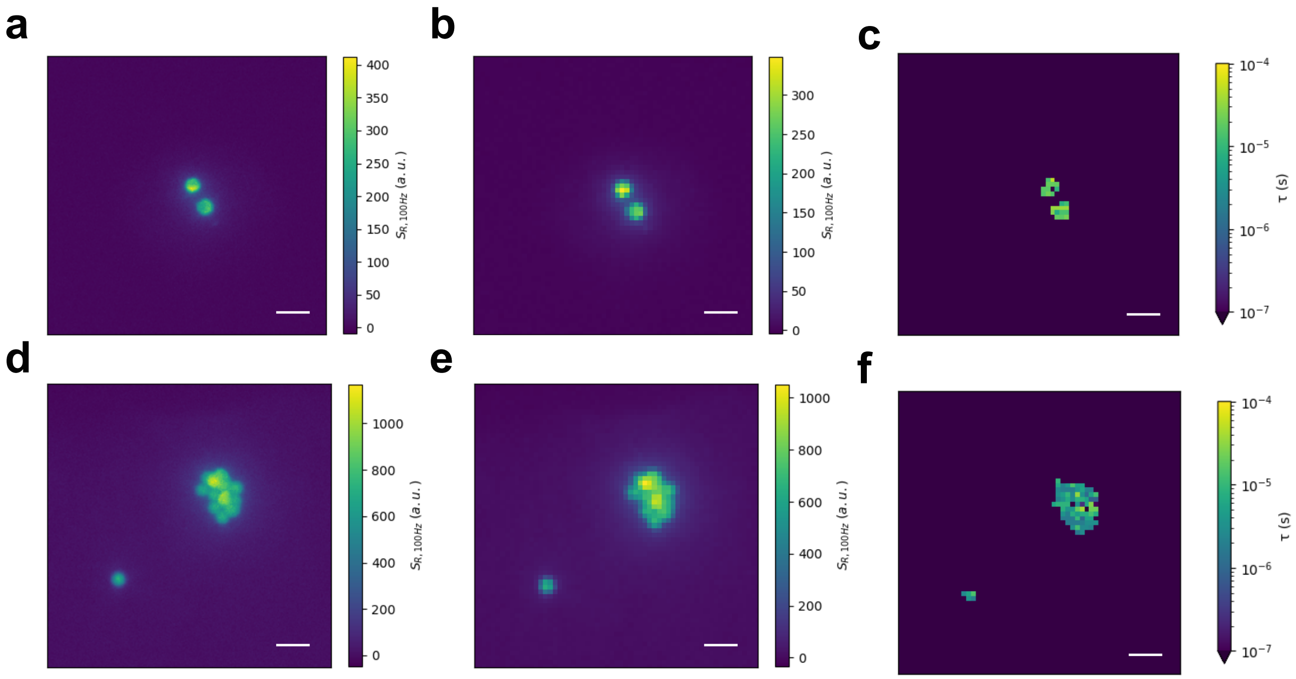

Figure S8: *RIOM wide-field images of 4.5 μm diameter labeled polystyrene beads under sinusoidally modulated illumination in epifluorescence microscopy.* **a,b,d,e**: 12 Hz acquired image of the RIOM signal from metal complexes and dye without (**a**,**d**) and with (**b**,**e**) 4 binning: **a**,**b**: Palladium octaethylporphyrin (PdOEP; $\lambda_{\rm exc} = 405$ nm, $\lambda_{\rm em} = 675$ nm; $I = 8$ mE.m^{-2}.s^{-1}, 0.24 W.cm^{-2}; $\omega = 628$ rad.s^{-1}, $f = 100$ Hz); **d**,**e**: Platinum octaethylporphyrin (PtOEP; $\lambda_{\rm exc} = 405$ nm, $\lambda_{\rm em} = 675$ nm; $I = 30$ mE.m^{-2}.s^{-1}, 0.91 W.cm^{-2}; $\omega = 628$ rad.s^{-1}, $f = 100$ Hz). See Table S4; **c,f**: RIOM image of the photoconversion time of PdOEP (**c**) and PtOEP (**f**) retrieved from the dependence of the images **b** and **e** respectively on the frequency of the modulated light. See Table S7. Scale bar: 10 μm.

The photoconversion time retrieved by RIOM for the 3 μm PtOEP beads, 4.5 μm PtOEP beads, and 4.5 μm PdOEP beads was compared to the photoconversion time obtained by using demodulation. The modulated amplitude M_A of the emission signal of the beads loaded with the metal complexes was retrieved by using synchronous detection and its dependence on the frequency of modulation (Figure S9) enabled to determine the phosphorescence lifetime of the metal complexes with Eq.(78).

$$M_A(f) = \frac{p_1}{\sqrt{1 + (2\pi f \tau)^2}} + p_2 \qquad (78)$$

Here the sample preparation was identical for each sample to experiments for RIOM signal acquisition. Each sample

was excited between $[10\text{ Hz}; 400\text{ kHz}]$.

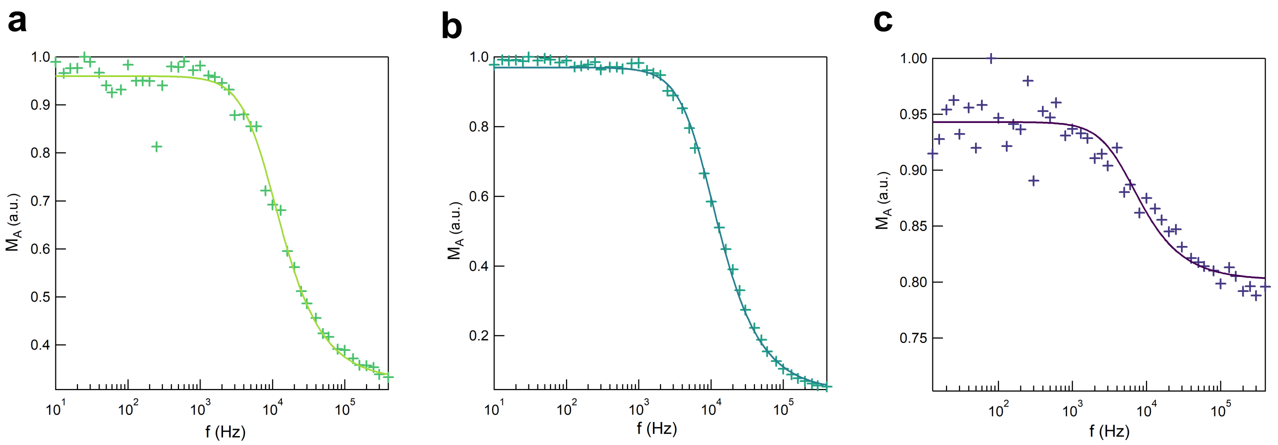

Figure S9: *Dependence of the normalized amplitude of the modulation M_A of microbeads labeled with metal complexes on the frequency of the modulated light excitation f.* **a** : 3 μm Platinum octaethylporphyrin (PtOEP; $\lambda_{\text{exc}} = 540$ nm, $\lambda_{\text{em}} = 675$ nm; $I = 8$ mE.m^{-2}.s^{-1}, 1.76 W.cm^{-2}); **b**: 4.5 μm Platinum octaethylporphyrin (PtOEP; $\lambda_{\text{exc}} = 540$ nm, $\lambda_{\text{em}} = 675$ nm; $I = 140$ mE.m^{-2}.s^{-1}, 3.17 W.cm^{-2}); **c**: 4.5 μm Palladium octaethylporphyrin (PdOEP; $\lambda_{\text{exc}} = 540$ nm, $\lambda_{\text{em}} = 675$ nm; $I = 140$ mE.m^{-2}.s^{-1}, 3.17 W.cm^{-2}). See Tables S5 and S8 for the parameters of acquisition and fitting respectively.

2.1.5.6 Arabidopsis Thaliana In all of these experiments, the measurement protocols were run while the sample was exposed to a sunlight condition, provided by the lime LED detailed in the setup section. Its illumination power at the sample was set to be 160 μE.m^{-2}.s^{-1} – a small amount more than the light intensity that the plants were grown under. In all of the experiments detailed below, the fluorescence was imaged through a 690/50 nm bandpass filter (AT690/50m, Chroma Technology Corp., Bellows Falls, VT, US).

HIOM with leaves At the beginning of the measurement protocol, the leaves were in a dark-adapted state. The protocol begun with the exposure of the leaf, for 15 minutes, to a constant 470 nm light at 100 μE.m^{-2}.s^{-1}, supplied using the combined light intensity of the 470 nm LEDs on the right and left illumination paths. It is important to mention here that this is on top of the artificial sunlight which has been added with the lime LED – it is present throughout the whole protocol. After the 15 minutes of constant light, the oscillation protocol began. The frequencies explored, in Hz, for one illumination arm were [3, 6, 9, 17, 29, 53, 101, 197, 389, 773, 1541, 3077, 6149, 12293, 24581, 49157, 98309, 196613, 393221, 393221, 196613, 98309, 49157, 24581, 12293, 6149, 3077, 1541, 773, 389, 197, 101, 53, 29, 17, 9, 6, 3]. At the second illumination arm the frequencies were 1 Hz lower than those of the first illumination arm and began in antiphase with respect the first illumination arm. Both arms were modulated between 0 μE.m^{-2}.s^{-1} and two times the constant level used in the constant light step, giving a high value of 200 μE.m^{-2}.s^{-1}, for all frequencies. At each frequency, the light level was held at the constant level for 1 second, followed by 6 seconds of oscillation, and finally 1 second of the constant level again. During all of this, the camera was set to capture frames at 12 frames per second,

with an exposure time of 80 ms. Then after this 8 s acquisition block, a recovery time, whereby the leaf remains exposed to the constant light level, was applied for 6 seconds, during which no frames were recorded, and after which, the next frequency was tested.

In the first step in regard to data processing, a signal was extracted using an ROI on the leaf within the video. Only valid pixels were included in the extraction. In order to screen pixels for inclusion, the signal for each pixel, over the number of frames, was examined and only included in the final signal if it never exceeded a level of 4000, close to the maximum possible pixel level of 4096, or dropped below 200, a very low level. The resulting signal was then processed by, at each frequency, extracting the quadrature delayed amplitude for the signal section between 2 seconds and 4 seconds. A plot of amplitude vs frequency, i.e., a Bode plot, for the untreated leaf, and only for the frequencies swept from low to high, is provided in Figure 2 of the Main Text. In Figure S10 the same plot is provided but for all data points, i.e., the full increasing and decreasing frequency sweeps. It allows for it to be seen that hysteresis is not present. This is important since the response of the leaf can change with time due to regulatory processes. We have some confidence therefore that there are no significant changes to the leaf's state over the protocol duration. Another important verification that was performed was to confirm that the equipment did not result in some characteristic in the Bode diagram. In order to do so, a fluorophore, Rhodamine B in ethanol (30 μM), was used where a flat response was expected. During each of the experiments, a solution of this fluorophore, held between two standard microscope cover slips with a spacer, was held in position near to the leaf to provide a reference. The resulting Bode diagram (Figure S10) depicts a flat response, verifying that the response is actually due to the sample and not the equipment.

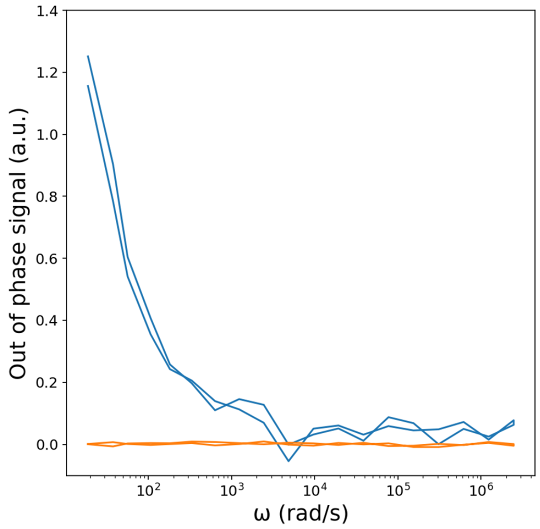

Figure S10: *Bode diagrams of the response from an untreated leaf (BLUE) and Rhodamine B in ethanol (30 μM, ORANGE) using the HIOM protocol, taken over an ROI within the video frames.*

In the case of being able to image the kinetic response of the plant, processing needed to be applied to each pixel of the image. In order to improve the signal to noise ratio, and reduce processing time, the frames were downsampled

by a factor of 4 prior to processing. Then, by using thresholding, the vast majority of pixels not including the leaf were masked. The processing detailed above, for extracting the Bode diagrams, followed by calculation of the ratio between the amplitude at 1 and 773 Hz, was applied to each unmasked pixel of the downsampled image. The resulting images obtained for the treated and untreated cases are displayed in the Main Text of this manuscript.

RIOM with leaves This experiment took a very similar form to the previous. The untreated and treated leaves were prepared in the same manner. As a reminder, the sunlight condition was applied during the complete duration of the experiment. It began with exposure of the sample to a constant 470 nm light at 100 μE.m^{-2}.s^{-1} for 15 minutes followed by the oscillating protocol. The protocol involved, for each frequency, a 10 s constant phase (at the average intensity of the oscillation waveform) followed by a 10 s oscillating phase, whereby the light is oscillated between 0 μE.m^{-2}.s^{-1} and 200 μE.m^{-2}.s^{-1}. During this, camera frames were acquired at a frequency of 0.5 Hz, with an exposure time of practically 1 s. The frequencies included, in Hz, were the following: [1, 2, 4, 8, 10, 20, 39, 74, 138, 256, 475, 879, 1196, 2213, 4094, 7573, 20000, 40000, 60000, 80000, 80000, 60000, 40000, 20000, 7573, 4094, 2213, 1196, 879, 475, 256, 138, 74, 39, 20, 10, 8, 4, 2, 1].

In regard to data processing, the signal within an ROI was extracted in the same manner as in the previous experiment, whereby only pixels whose signal never exceeds 4000 or drops below 200 are kept. The resulting signal is shown in Figure S11. It can be seen that globally the signal drifts, due to the complex regulatory mechanisms of the photosynthetic apparatus. In order to account for such drifts, the response value retained for each frequency was the data point value just after beginning oscillation, divided by the data point value just before oscillation, giving a form of relative response. This, plotted against frequency, gave the Bode diagram. The Bode diagram obtained with the untreated leaf is shown in Figure S11 The response is shown for where the frequencies were swept from low to high, as well as for high to low.

As was the case in the HIOM experiment, as can be seen in Figure S11 the response shows little hysteresis when retesting the same frequencies in the second half of the protocol where the frequencies are run in the reverse order. It was the case here too that the Bode diagrams for the illumination light showed a flat response (Figure S11 providing confidence that any response seen was in fact as a result of the sample and not the equipment. This was made possible by delivering light directly from the LED to the edge of the image sensor using an optical fiber (M137L02, Thorlabs Inc., Newton, NJ, US).

Regarding being able to image the kinetic response of the plant, processing needed to be applied to each pixel of the image. In order to improve the signal to noise ratio, and reduce processing time, the frames were downsampled by a factor of 10 prior to processing. It was seen that there were small reflection spots from the leaf, likely from the reflecting artificial sunlight, whereby the spots saturated the pixel range. In light of this, during downscaling, only pixels which did not, for all frames corresponding to that pixel, exceed 4000 or drop below 200, were included in the downscaling process. Following this, segmentation of the leaf area was performed by thresholding and blob extraction. The processing detailed above, for extracting the Bode diagrams, was applied to each pixel containing the leaf in the downsampled image. The

value retained for each pixel of the resulting image was taken as the ratio between the response at 1 and 39 Hz. The resulting images obtained are displayed in the Main Text of this manuscript.

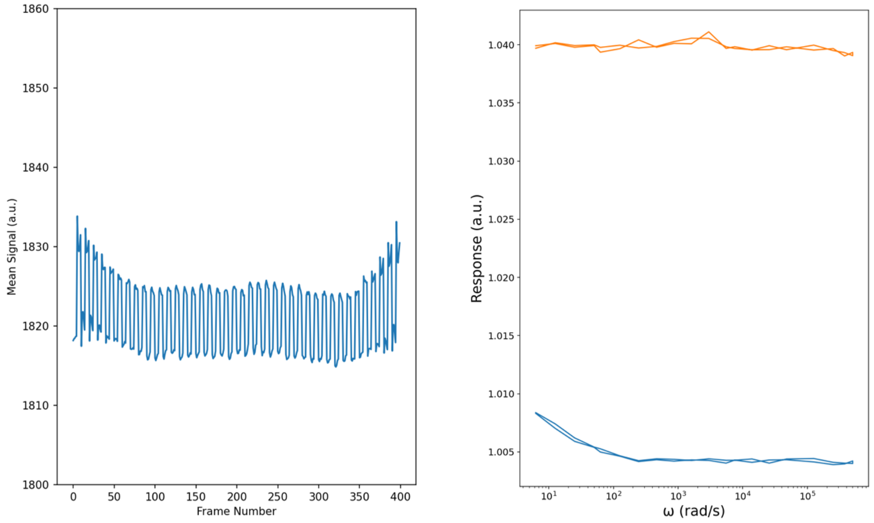

Figure S11: *Mean signal within an ROI (LEFT) for the untreated leaf and Bode diagrams (RIGHT) corresponding to the response of the untreated leaf (blue) and for the excitation light (orange).*

RIOM for investigating DCMU uptake In this experiment, a small but full Arabidopsis plant was transplanted, retaining soil from its original pot, into a small 35 mm diameter plastic Petri dish (Thermo Fisher Scientific, Waltham, MA, US). In general terms, the experiment involved the addition of DCMU to the roots of the plant and the progression of the inhibitor into the plant followed by periodically applying the RIOM protocol, all under the artificial sunlight condition. The experiment began with exposure of the plant to a constant 470 nm light at 100 μE.m^{-2}.s^{-1} for 15 minutes, after which the first RIOM measurement was taken, using the same 470 nm light source. The measurement involved a 10 s constant part (at the average intensity of the oscillation waveform) followed by a 10 s oscillating part, whereby the light was sinusoidally oscillated between 0 μE.m^{-2}.s^{-1} and 200 μE.m^{-2}.s^{-1}, all while the camera captured frames at 0.5 Hz with an exposure time of practically 1 s. The measurements needed to be taken more rapidly than in the RIOM experiment on individual leaves since a dynamic process was being followed, and it was necessary that the sample did not change significantly over the measurement duration. Thus, only the following frequencies were used in this protocol: [1, 1, 4, 10, 39, 138, 475, 1196, 4094, 20000, 60000, 80000]. 5 minutes after the first measurement, the Petri dish was filled with the DCMU solution, until almost the maximum level possible, to ensure contact of the roots with the solution. The measurements were continued, every 14 minutes, for almost 24 hours. Images of the response were determined in the manner detailed in the previous experiment; however masking of pixels was performed in a slightly different manner, no downsizing was performed, and the frequencies used for the ratio making up the final kinetic image were 0 and 4094 Hz. The masking protocol relied upon checking for pixels which followed the expected step-like signal (due to the constant and oscillatory parts for each frequency) but is not provided here. Its precise details are available in the supplied code.

7 of the obtained RIOM images, showing the uptake of DCMU from 23 minutes after DCMU addition, up to almost 15 hours after, are provided in the Main Text. Also provided, in order to allow comparison against that obtained by simply using direct fluorescence, is the first image taken at each measurement time point, where the plant was under constant light.

2.1.6 Acquisition parameters used for HIOM and RIOM imaging

The acquisition parameters, which have been used for HIOM and RIOM imaging are reported in Tables S3 and S4. The acquisition parameters used to acquire the photoconversion time by demodulation with synchronous detection are reported in Table S5.

Table S3: Acquisition parameters used to acquire the HIOM images in the Figures of the Main Text and the Supplementary Information.

Figure	Excitation	Frame number per frequency	λ_1 (nm)	I_1^0 (Ein.m^{-2}.s^{-1})	λ_2 (nm)	I_2^0 (Ein.m^{-2}.s^{-1})	$[f_{\min};f_{\max}]$ (Hz)	Δf (Hz)	α %
2a	Sine wave	144	470	0.25	405	0.13	[1;100]	1	100
S6a	Square wave	48	470	0.10	405	0.16	[1;400]	0.5	84
3g,j	Sine wave	96	470	5 10^{-5}	470	5 10^{-5}	[3;393221]	1	100

Table S4: Acquisition parameters used to acquire the RIOM images in the Figures of the Main Text and the Supplementary Information.

Figure	Excitation	Frame number per frequency	λ_1 (nm)	I_1^0 (Ein.m^{-2}.s^{-1})	λ_2 (nm)	I_2^0 (Ein.m^{-2}.s^{-1})	$[f_{\min};f_{\max}]$ (Hz)	α %
2d	Sine wave	24	470	0.07	405	0.15	[1;100]	100
S6d	Square wave	48	470	0.10	405	0.16	[1;400]	84
S4	Sine wave	4	470	1.8 10^{-3}	405	3.6 10^{-3}	[0.01;100]	100
2g,S7,S8a	Sine wave	12	405	0.008	–	–	[100;10^5]	100
2j,S7,S8d	Sine wave	12	405	0.03	–	–	[100;10^5]	100
3a,d	Sine wave	10	470	1 10^{-4}	–	–	[1;80000]	100
3m	Sine wave	10	470	1 10^{-4}	–	–	[1;80000]	100

Table S5: Acquisition parameters used to acquire the photoconversion time by demodulation with synchronous detection in the Figures of the Main Text and the Supplementary Information.

Figure	Excitation	λ_1 (nm)	I_1^0 (Ein.m^{-2}.s^{-1})	λ_2 (nm)	I_2^0 (Ein.m^{-2}.s^{-1})	$[f_{\min};f_{\max}]$ (Hz)	α %
S9a	Sine wave	540	0.08	–	–	[10;4.10^5]	100
2l,S9b	Sine wave	540	0.14	–	–	[10;4.10^5]	100
2i,S9c	Sine wave	540	0.14	–	–	[10;4.10^5]	100

2.1.7 Fitting parameters used in HIOM and RIOM images

The fitting parameters, which have been used in HIOM and RIOM images are reported in Tables S6 and S7. The fitting parameters used to retrieve the photoconversion time by demodulation with synchronous detection are reported in Table S8.

Table S6: Fitting parameters used in the HIOM images in the Figures of the Main Text and the Supplementary Information.

Figure	Excitation	Fitting function	γ	p_1	p_2	p_3	τ (ms)
2b	Sine wave	Eq.(74)	1	-27722	2.9	126	12.8 ± 0.5
S6b	Square wave	Eq.(74)	1.85	-3997	3.16	24.96	8.8 ± 0.4

Table S7: Fitting parameters used in the RIOM images in the Figures of the Main Text and the Supplementary Information.

Figure	Excitation	Fitting function	p_1	p_2	p_3	τ (ms)
2e	Sine wave	Eq.(73)	-330	0.55	2034	8.2 ± 1.4
S6e	Square wave	Eq.(73)	-3055	0.59	8897	14.4 ± 0.2
S4	Sine wave	Eq.(73)	-12.6	0.766	82.4	480 ± 16
2i,S8c	Sine wave	Eq.(73)	-3.34	0.53	203	0.014 ± 0.006
2l,S8f	Sine wave	Eq.(73)	-8.29	0.43	620	0.0045 ± 0.002

Table S8: Fitting parameters used to retrieve the photoconversion time by demodulation with synchronous detection in the Figures of the Main Text and the Supplementary Information.

Figure	Excitation	Fitting function	p_1	p_2	τ (ms)
S9a	Sine wave	Eq.(78)	0.63	0.33	0.021 ± 0.001
S9b	Sine wave	Eq.(78)	0.93	0.041	0.021 ± 0.002
S9c	Sine wave	Eq.(78)	0.14	0.80	0.034 ± 0.006

In the following sections, we theoretically compute the dependence of the RIOM and HIOM signals of representative reversibly photo-convertible luminophores on the average intensity/ies and angular frequency/ies of different types of periodically modulated illumination. Hence, we extract the dependence of $\mathfrak{2}^{\mathfrak{0}}$, and $\mathfrak{2}^{\mathfrak{1},-\mathfrak{1},\mathfrak{sin}}$ and $\mathfrak{2}^{\mathfrak{1},-\mathfrak{1},\mathfrak{cos}}$ (governing the behavior of the phosphorescent probes in RIOM and HIOM respectively), and $\mathfrak{I}_{\mathfrak{F}}^{\mathfrak{0}}$, and $\mathfrak{I}_{\mathfrak{F}}^{\mathfrak{1},-\mathfrak{1},\mathfrak{sin}}$ and $\mathfrak{I}_{\mathfrak{F}}^{\mathfrak{1},-\mathfrak{1},\mathfrak{cos}}$ (governing the behavior of the other presently considered probes in RIOM and HIOM respectively).

A Sinusoidal modulation at a single angular frequency

In relation to the RIOM protocol, we first considered for $I(t)$ an illumination modulated at the angular frequency of modulation ω and involving in the most general case the superposition of two lights at wavelengths λ_1 (around the averaged value I_1^0) and λ_2 (around the averaged value I_2^0). We adopted the general expression, which is given in Eq.(79):

$$I(t) = I_1^0\left[1+\alpha h_1(t)\right]+I_2^0\left[1+\alpha\delta h_2(t)\right] \tag{79}$$

$$h_1(t) = \sin\left(\omega t\right) \tag{80}$$

$$h_2(t) = \sin\left(\omega t+\varphi\right) \tag{81}$$

It is applied on a luminophore for which the components of illumination $I_1(t)$ and $I_2(t)$ preferentially promote the photoconversion of the state **1** to the state **2** ($\sigma_{12,1} \gg \sigma_{21,1}$), and the one of the state **2** to the state **1** ($\sigma_{12,2} \ll \sigma_{21,2}$) respectively.

A.1 Light modulation at a single wavelength

We first examined the case of light modulation at a single wavelength λ_1 (around the averaged value I_1^0) ($\delta = 0$ in Eq.(79)).

A.1.1 Generic maps

Following the general derivation given in subsection 1.2.1, we analytically retrieved the $2n+1$ unknown terms (a^0,…,a^n,b^n) upon truncating the Fourier expansion (30) at the 7th order. We then established the map of the deviation of the rectification of

- $\mathfrak{2}^{\mathfrak{0}}$ with respect to the luminescence signal 2^0 obtained under constant illumination to address the case of the behavior of the phosphorescent probes in RIOM;

- $\mathfrak{I}_{\mathrm{F}}^{\mathfrak{0}}$ with respect to the luminescence signal I_{F}^0 obtained under constant illumination given in Eq.(90)

$$I_{\mathrm{F}}^0 = \left(Q_{1,1}1^0+Q_{2,1}2^0\right)I_1^0+\left(Q_{1,2}1^0+Q_{2,2}2^0\right)I_2^0 \tag{82}$$

  to address the case of the behavior of the other presently considered fluorescent probes in RIOM.

The result is displayed in Figure S12. It makes clear the range of $\{K_{12}^0, \theta\}$ values for which a significant rectification of the luminescence signal of the probe P can be observed: K_{12}^0 should typically range between $10^{-0.5}$ and $10^{2.5}$ whereas θ should be lower than 10^0 for the phosphorescent probes, and K_{12}^0 should typically range between 10^{-2} and 10^1 whereas θ should be lower than 10^0 for the other presently considered fluorescent probes.

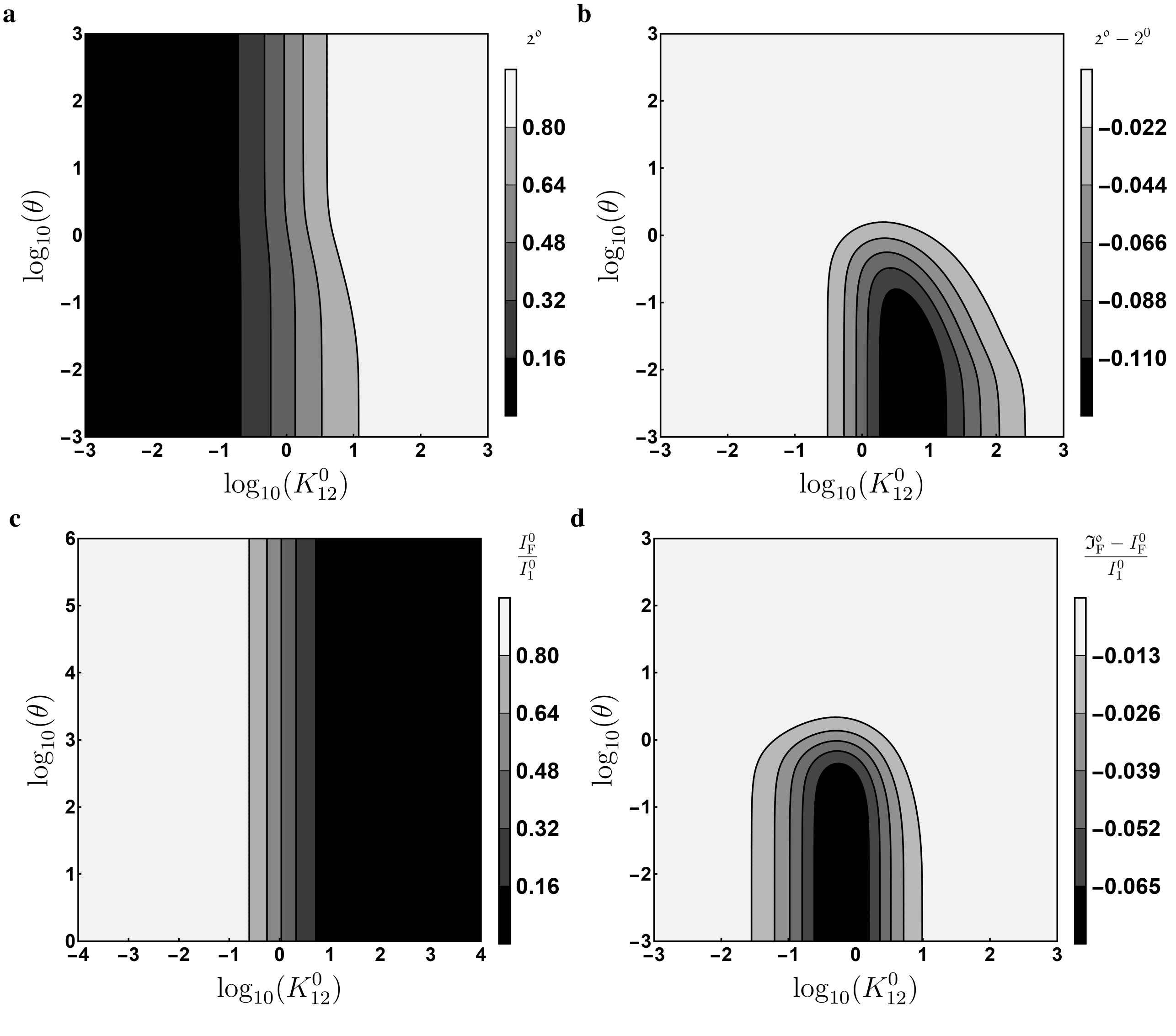

Figure S12: *Dependence of the normalized values of $\mathfrak{z}^{\circ}$ (**a**) and $\mathfrak{z}^{\circ} - 2^0$ (**b**), and I_F^0 (**c**) and $\mathfrak{I}_F^{\circ} - I_F^0$ (**d**) on the dimensionless angular frequency θ and on the constant K_{12}^0 for a luminophore ($P_{\rm tot} = 1$ M, and $Q_{1,1} = 1$, $Q_{1,2} = 0$, $Q_{2,1} = 0$, and $Q_{2,2} = 0$ in **c** and **d**) submitted to the reaction (1) under sinusoidal modulation at a single angular frequency of modulation at the wavelength λ_1 with $\alpha = 1$.*

A.1.2 Dimensionalized maps

The effective implementation of the rectification of the luminescence signal requires to dimensionalize the maps displayed in Figure S12.

A.1.2.1 Phosphorescent probes We first considered the case of phosphorescent probes, which can be photoactivated at the wavelength λ_1 governing the photoconversions from the state 1 to the state 2. Figure S13a displays the dependence of the normalized phosphorescence z° on the angular frequency ω and on the light intensity I_1^0 for a phosphorescent probe, which is characterized by $\lambda_1 = 405$ nm, $\sigma_{12,1} = 50$ m^2.mol^{-1}, $\sigma_{21,1} = 0$ m^2.mol^{-1}, and $k_{21}^{\Delta} = 2 \times 10^5$ s^{-1}. Figure S13b further evaluates the dependence of the normalized difference $z^{\circ}\left(\omega = 10^{10}\right) - z^{\circ}\left(\omega = 10^{0}\right)$ on the light intensity I_1^0, which shows that it is possible to recover a significant rectification of the phosphorescence signal of the probe over a wide range of light intensity I_1^0. At the optimal I_1^0 value, z° increases by 13% when going from low to high frequency of light modulation.

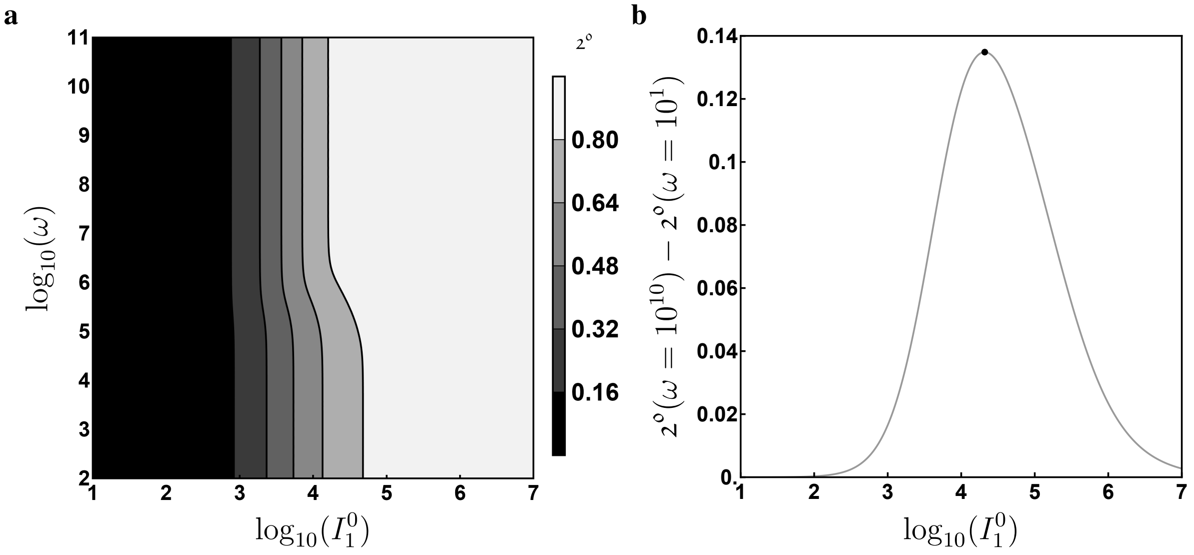

Figure S13: *Dependence of the normalized phosphorescence* z° *on the angular frequency* ω *and on the light intensity* I_1^0 *(**a**) and of the normalized difference* $z^{\circ}\left(\omega = 10^{10}\right) - z^{\circ}\left(\omega = 10^{1}\right)$ *on the light intensity* I_1^0 *(in* Ein.m^{-2}.s^{-1}*) (**b**) for a phosphorescent probe (*$P_{\rm tot} = 1$ M*,* $\sigma_{12,1} = 50$ *m*^{2}*.mol*$^{-1}$*,* $\sigma_{21,1} = 0$ *m*^{2}*.mol*$^{-1}$*, and* $k_{21}^{\Delta} = 2\ 10^5$ *s*$^{-1}$ *submitted to the reaction (1) under sinusoidal modulation at a single angular frequency of modulation at the wavelength* λ_1 *with* $\alpha = 1$.

In order to retrieve the relaxation time τ_{12}^0, one has to process the dependence of z° on the angular frequency ω. To identify a relevant fitting function, we introduced the time dependence of the applied modulated illumination given in Eqs.(79–81) into the master differential equation given in Eq.(21) and retrieved analytic expressions of the time varying terms contained in Eq.(37) upon truncating the Fourier expansion (30) at the 2$^{\rm nd}$ order. Then we analyzed the mathematical structure of the resulting function and we decided to adopt the fitting function given in Eq.(83)

$$\mathcal{L}(\omega, p_1, p_2, p_3) = \frac{p1(1 + 4(\omega\tau_{12}^0)^2)}{p_2 + 5(\omega\tau_{12}^0)^2 + 4(\omega\tau_{12}^0)^4} + p_3 \tag{83}$$

in order to extract the relaxation time $\tau_{12}^{0,\rm fit}$ from the computed dependence of the normalized luminescence z° of the phosphorescent probe on the angular frequency ω for various values of the light intensity I_1^0. Figure S14a displays the results. Figure S14b displays the dependence of the fitted and expected relaxation times $\tau_{12}^{0,\rm fit}$ and $\tau_{12}^{0,\rm th}$ on the light

intensity I_1^0. It shows that there is a satisfactory agreement over the $[10^{2.5}; 10^{4.7}]$ range[8] of light intensity I_1^0 expressed in Ein.m^{-2}.s^{-1}.

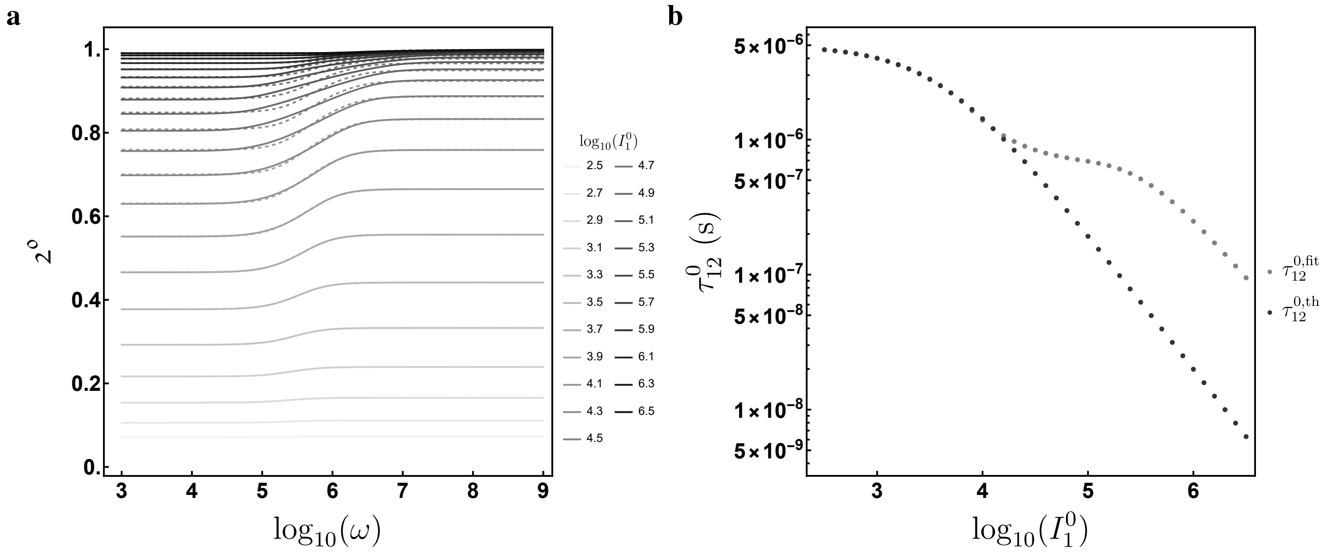

Figure S14: *Retrieval of the relaxation time τ_{12}^0 from processing the dependence of $\mathfrak{I}^{o}$ on the angular frequency ω.* **a**: Dependence of the normalized luminescence $\mathfrak{I}^{o}$ on the angular frequency ω for various values of the light intensity I_1^0. Solid line: Numerical computation; Dashed line: Fit with Eq.(83); **b**: Dependence of the fitted and expected relaxation times $\tau_{12}^{0,\mathrm{fit}}$ and $\tau_{12}^{0,\mathrm{th}}$ on the light intensity I_1^0 (in Ein.m^{-2}.s^{-1}) (**b**). Luminophore: Phosphorescent probe ($P_{\mathrm{tot}} = 1$, $Q_{1,1} = 1$, $Q_{1,2} = 0$, $\sigma_{12,1} = 50$ m^2.mol^{-1}, $\sigma_{21,1} = 0$ m^2.mol^{-1}, and $k_{21}^{\Delta} = 2\ 10^5$ s^{-1}) submitted to the reaction (1) under sinusoidal modulation at a single angular frequency of modulation at the wavelength λ_1 with $\alpha = 1$.

A.1.2.2 Reversibly photo-convertible fluorophores

We then considered the case of reversibly photo-convertible fluorophores, which can be photoswitched at two wavelengths λ_1 and λ_2 governing the photoconversions from the state 1 to the state 2 and from the state 2 to the state 1 respectively. Figure S15a displays the dependence of the normalized luminescence $\mathfrak{I}_F^o$ on the angular frequency ω and on the light intensity I_2^0 for the reversibly photo-convertible fluorescent protein Dronpa-2, which is characterized by $\lambda_1 = 488$ nm and $\lambda_2 = 405$ nm, $\sigma_{12,1} = 198$ m^2.mol^{-1}, $\sigma_{21,1} = 0$ m^2.mol^{-1}, $\sigma_{12,2} = 0$ m^2.mol^{-1}, and $\sigma_{21,2} = 415$ m^2.mol^{-1}, and $k_{21}^{\Delta} = 1.4\ 10^{-2}$ s^{-1}.[5] Figure S15b further evaluates the dependence of the normalized difference $\mathfrak{I}_F^o\left(\omega = 10^{10}\right) - \mathfrak{I}_F^o\left(\omega = 10^{0}\right)$ on the light intensity I_2^0, which shows that it is possible to recover a significant rectification of the fluorescence signal of Dronpa-2 over a wide range of light intensity I_2^0. At the optimal I_2^0 value, $\mathfrak{I}_F^o$ increases by 8% when going from low to high frequency of light modulation.

[8] This range results from accepting up to 50% of difference between $\tau_{12}^{0,\mathrm{fit}}$ and $\tau_{12}^{0,\mathrm{th}}$. This threshold has been adopted for the definition of all subsequent similar ranges.

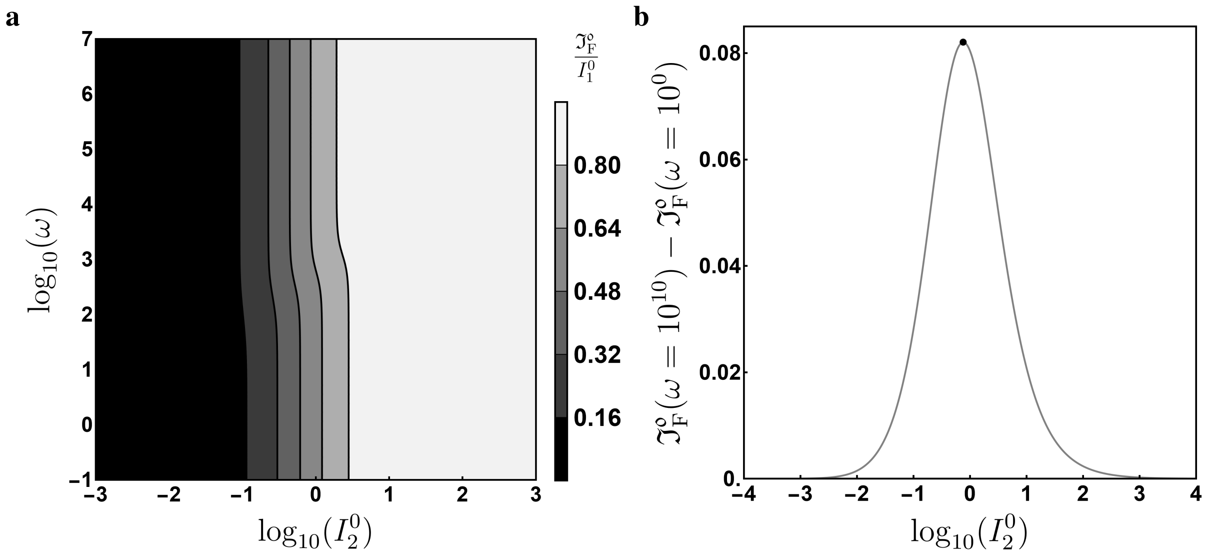

Figure S15: *Dependence of the normalized luminescence* $\mathfrak{I}_F^o$ *on the angular frequency* ω *and on the light intensity* I_2^0 *(**a**) and of the normalized difference* $\mathfrak{I}_F^o\left(\omega = 10^{10}\right) - \mathfrak{I}_F^o\left(\omega = 10^0\right)$ *on the light intensity* I_2^0 *(in* Ein.m^{-2}.s^{-1}*) (**b**) for Dronpa-2 (*$P_{\mathrm{tot}} = 1$ M*,* $Q_{1,1} = 1$*,* $Q_{1,2} = 0$*,* $Q_{2,1} = 0$*,* $Q_{2,2} = 0$*,* $\sigma_{12,1} = 198$ *m*^{2}*.mol*$^{-1}$*,* $\sigma_{21,1} = 0$ *m*^{2}*.mol*$^{-1}$*,* $\sigma_{12,2} = 0$ *m*^{2}*.mol*$^{-1}$*,* $\sigma_{21,2} = 415$ *m*^{2}*.mol*$^{-1}$*, and* $k_{21}^{\Delta} = 1.4\ 10^{-2}$ *s*$^{-1}$ *submitted to the reaction (1) under sinusoidal modulation at a single angular frequency of modulation at the wavelength* λ_1 *with* $\alpha = 1$ *and* $I_1^0 = 1$ *Ein.m*$^{-2}$*.s*$^{-1}$.

In order to retrieve the relaxation time τ_{12}^0, one has to process the dependence of $\mathfrak{I}_F^o$ on the angular frequency ω. To identify a relevant fitting function, we introduced the time dependence of the applied modulated illumination given in Eqs.(79–81) into the master differential equation given in Eq.(21) and retrieved analytic expressions of the time varying terms contained in Eqs.(49,53) upon truncating the Fourier expansion (30) at the 2nd order. Then we analyzed the mathematical structure of the resulting function and we decided to adopt the fitting function given in Eq.(84)

$$\mathcal{L}(\omega, p_1, p_2, p_3) = \frac{p1(1 + 4(\omega\tau_{12}^0)^2)}{p_2 + 5(\omega\tau_{12}^0)^2 + 4(\omega\tau_{12}^0)^4} + p_3 \tag{84}$$

in order to extract the relaxation time $\tau_{12}^{0,\mathrm{fit}}$ from the computed dependence of the normalized luminescence $\mathfrak{I}_F^o$ of Dronpa-2 on the angular frequency ω for various values of the light intensity I_2^0. Figure S16a displays the results. Figure S16b displays the dependence of the fitted and expected relaxation times $\tau_{12}^{0,\mathrm{fit}}$ and $\tau_{12}^{0,\mathrm{th}}$ on the light intensity I_2^0. It shows that there is a satisfactory agreement over the $[10^{-1.4}; 10^2]$ range of light intensity I_2^0 expressed in Ein.m^{-2}.s^{-1}.

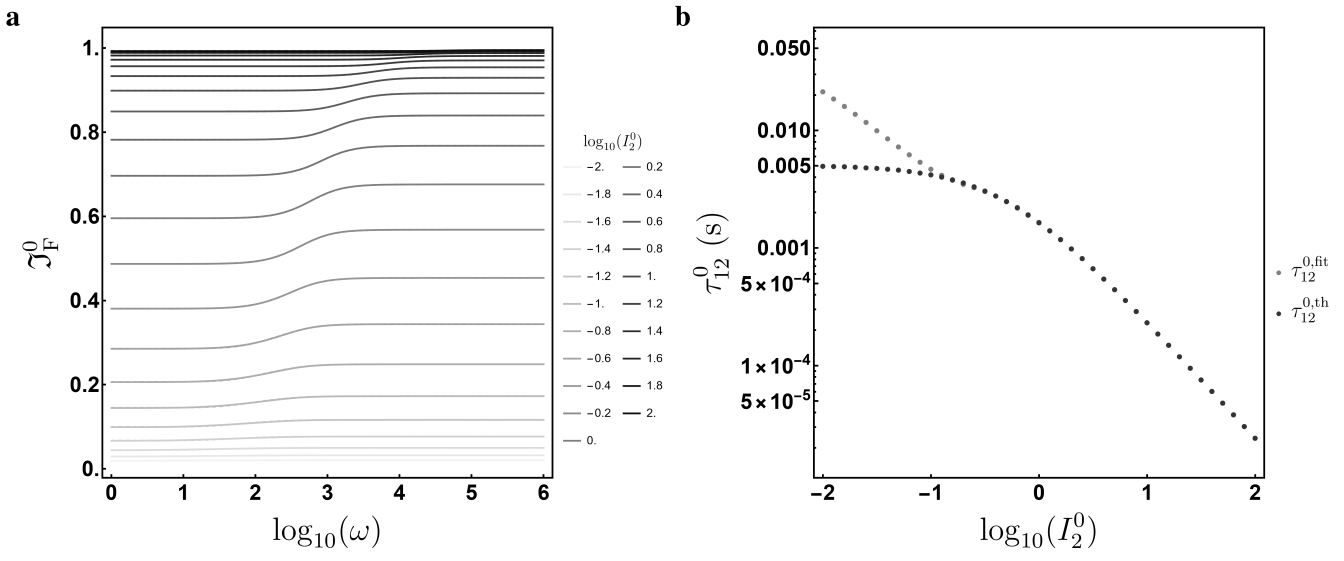

Figure S16: *Retrieval of the relaxation time τ_{12}^0 from processing the dependence of $\mathfrak{I}_F^{\circ}$ on the angular frequency ω.* **a**: Dependence of the normalized luminescence $\mathfrak{I}_F^{\circ}$ on the angular frequency ω for various values of the light intensity I_2^0. Solid line: Numerical computation; Dashed line: Fit with Eq.(84); **b**: Dependence of the fitted and expected relaxation times $\tau_{12}^{0,\mathrm{fit}}$ and $\tau_{12}^{0,\mathrm{th}}$ on the light intensity I_2^0 (in Ein.m^{-2}.s^{-1}) (**b**). Luminophore: Dronpa-2 ($P_{\mathrm{tot}} = 1$, $Q_{1,1} = 1$, $Q_{1,2} = 0, Q_{2,1} = 0, Q_{2,2} = 0, \sigma_{12,1} = 198\,\mathrm{m}^2.\mathrm{mol}^{-1}, \sigma_{21,1} = 0\,\mathrm{m}^2.\mathrm{mol}^{-1}, \sigma_{12,2} = 0\,\mathrm{m}^2.\mathrm{mol}^{-1}, \sigma_{21,2} = 415\,\mathrm{m}^2.\mathrm{mol}^{-1}$, and $k_{21}^{\Delta} = 1.4\ 10^{-2}\ \mathrm{s}^{-1}$) submitted to the reaction (1) under sinusoidal modulation at a single angular frequency of modulation at the wavelength λ_1 with $\alpha = 1$ and $I_1^0 = 1$ Ein.m^{-2}.s^{-1}.

A.2 Light modulation at two wavelengths

In relation to the RIOM protocol on fluorophores reversibly photo-convertible at two distinct wavelength (e.g. Dronpa-2), we then examined for $I(t)$ the superposition of two sinusoidal modulations of large amplitude at a same angular frequency of modulation ω at wavelengths λ_1 (around the averaged value I_1^0) and λ_2 (around the averaged value I_2^0) ($\delta = 1$ in Eq.(79)).

A.2.1 Significance of the phase lag φ

In a first step, we numerically analyzed how the phase lag φ affects the dependence on the dimensionless angular frequency θ and constant K_{12}^0 of the terms a^0, a^1, and b^1, which were expected to be involved in the expressions of $\mathfrak{O}_j^{\circ}$ and $\mathfrak{I}_F^{\circ}$.[2] The results are displayed in Figure S17. As anticipated, a vanishing phase lag would not promote any significant deviation of the concentrations in **1** and **2** states from their values under constant illumination. In contrast, a significant departure can be observed for the three terms a^0, a^1, and b^1 for $\varphi = \frac{\pi}{2}$, π, and $\frac{3\pi}{2}$. As intuitively expected, the most advantageous deviation is observed with $\varphi = \pi$ where the dual modulation exerts a push-pull effect on the extent of the reaction (1).

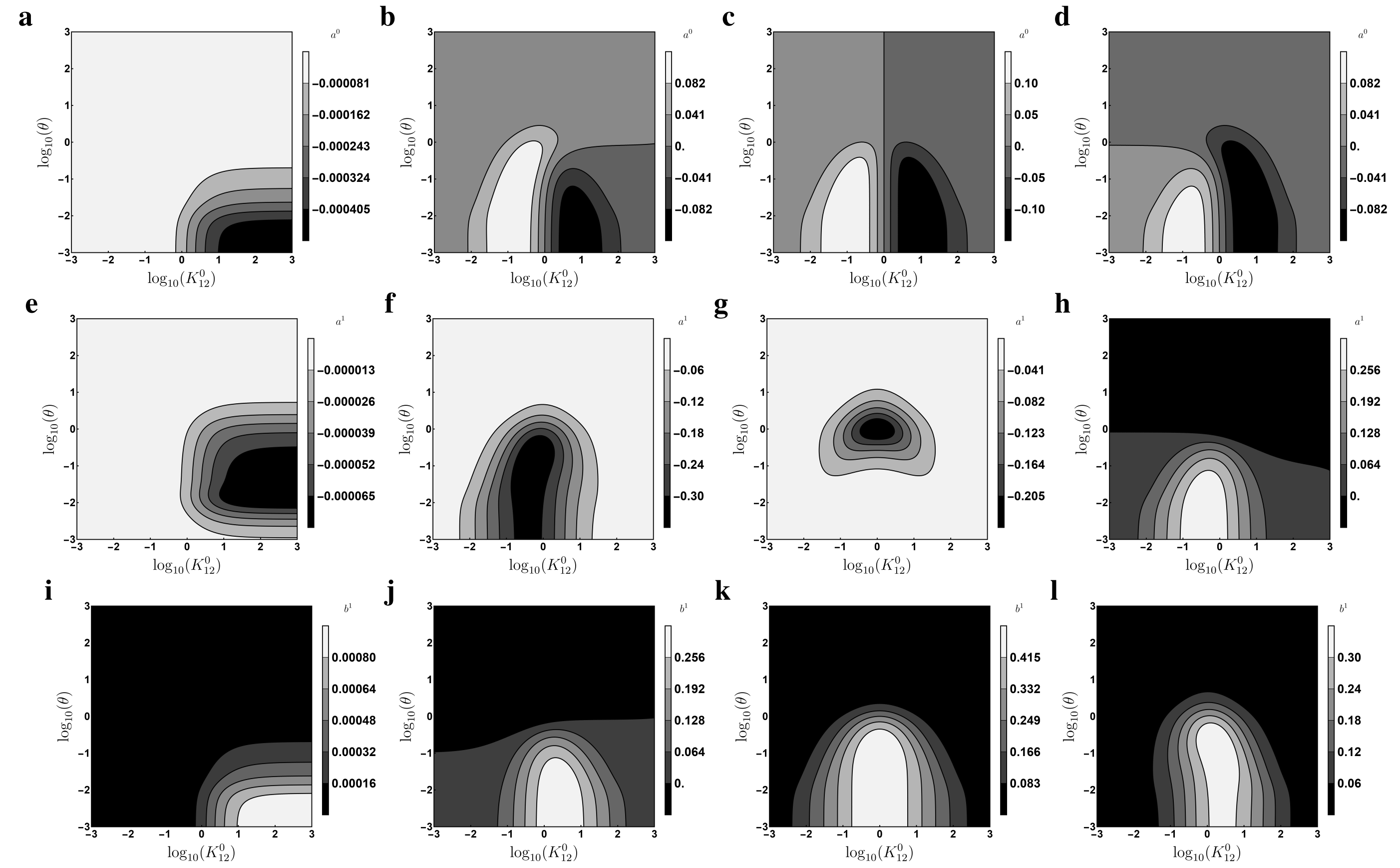

Figure S17: *Significance of the phase lag φ on the (θ,K^0_{12})-dependent terms a^0 (**a–d**), a^1 (**e–h**), and b^1 (**i–l**) deduced from the numerical solution of Eq.(21) truncated at the 7th order upon applying a sinusoidal modulation at a single angular frequency of modulation at two wavelengths with $\alpha = 1$ on a luminophore ($P_{\rm tot} = 1$) submitted to the reaction (1).* **a**,**e**,**i**: $\varphi = 0$; **b**,**f**,**j**: $\varphi = \frac{\pi}{2}$; **c**,**g**,**k**: $\varphi = \pi$; **d**,**h**,**l**: $\varphi = \frac{3\pi}{2}$.

We subsequently addressed how the phase lag φ affects the dependence of $\mathfrak{I}^{\rm o}_{\rm F}$ on the dimensionless angular frequency θ and constant K^0_{12}. The results are displayed in Figure S18. As for the concentrations, a vanishing phase lag does not make $\mathfrak{I}^{\rm o}_{\rm F}$ to depend on the dimensionless angular frequency θ at constant K^0_{12}. In contrast, such a dependence manifests itself with $\varphi = \frac{\pi}{2}$, π, and $\frac{3\pi}{2}$. Again, the most advantageous case is observed with $\varphi = \pi$ where the dependence is at the most pronounced.

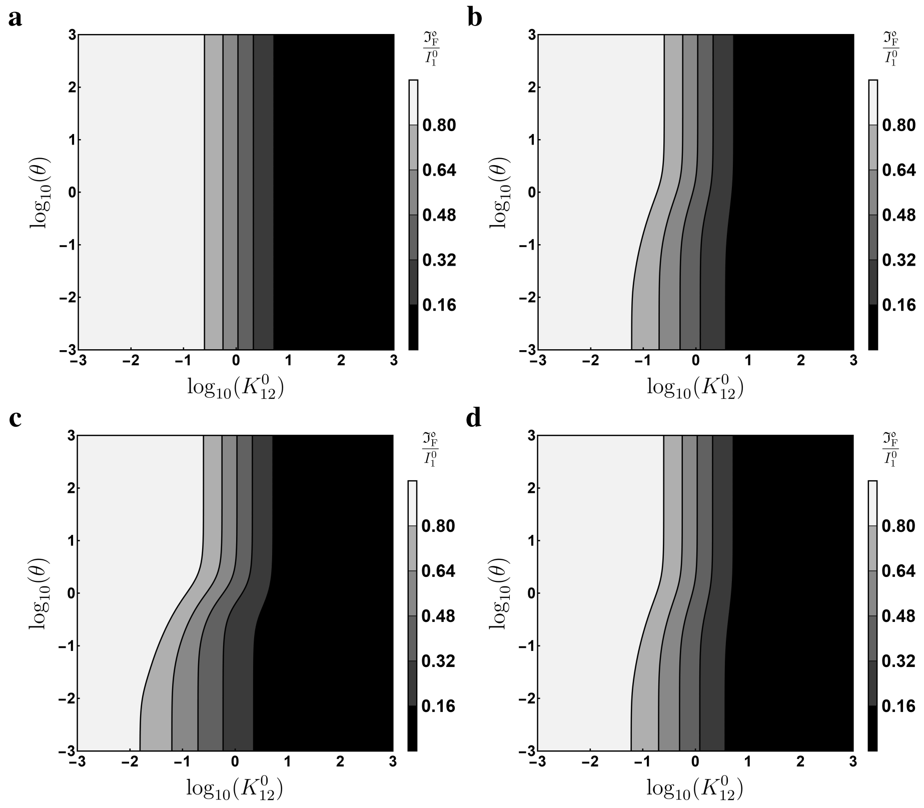

Figure S18: *Significance of the phase lag φ on the (θ, K^{0}_{12})-dependent fluorescence signal $\mathfrak{I}^{o}_{F}$ normalized by the light intensity I^{0}_{1} as computed with Eqs.(47–53) and the numerical solution of Eq.(21) truncated at the 7th order upon applying a sinusoidal modulation at a single angular frequency of modulation at two wavelengths with $\alpha = 1$ on a luminophore ($P_{\rm tot} = 1$ M, $Q_{1,1} = 1$, $Q_{1,2} = 0$, $Q_{2,1} = 0$, and $Q_{2,2} = 0$) submitted to the reaction (1).* **a**: $\varphi = 0$; **b**: $\varphi = \frac{\pi}{2}$; **c**: $\varphi = \pi$; **d**: $\varphi = \frac{3\pi}{2}$.

We are eventually interested in exploiting the dependence of $\mathfrak{I}^{o}_{F}$ on the angular frequency ω to selectively discriminate a targeted luminophore and retrieve the relaxation time associated with its reaction (1). Thus, we further analyzed the amplitude of the variation of $\mathfrak{I}^{o}_{F}$ over the investigated range of dimensionless angular frequency θ on the phase lag φ and on the constant K^{0}_{12}. As displayed in Figure S19, this amplitude exhibits a resonance with a maximum for $\varphi = \pi$ and $K^{0}_{12} = 10^{-0.66}$. The preceding result explains that we retained $\varphi = \pi$ in the following.

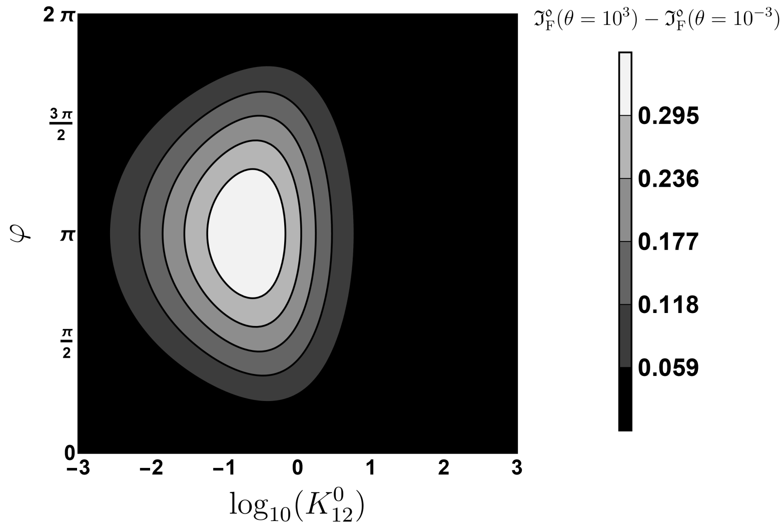

Figure S19: *Dependence of* $\mathfrak{I}_{\mathrm{F}}^{\circ}\left(\theta = 10^3\right) - \mathfrak{I}_{\mathrm{F}}^{\circ}\left(\theta = 10^{-3}\right)$ *on the phase lag* φ *and on the constant* K_{12}^0 *for a luminophore (*$P_{\mathrm{tot}} = 1$ M*,* $Q_{1,1} = 1$*,* $Q_{1,2} = 0$*,* $Q_{2,1} = 0$*, and* $Q_{2,2} = 0$*) submitted to the reaction (1) under sinusoidal modulation at a single angular frequency of modulation at two wavelengths with* $\alpha = 1$.

A.2.2 Generic maps

We first established the map of the deviation of the rectification of $\mathfrak{I}_{\mathrm{F}}^{\circ}$ with respect to the luminescence signal I_{F}^0 obtained under constant illumination given in Eq.(90)

$$I_{\mathrm{F}}^0 = \left(Q_{1,1}1^0 + Q_{2,1}2^0\right) I_1^0 + \left(Q_{1,2}1^0 + Q_{2,2}2^0\right) I_2^0. \quad (85)$$

The result is displayed in Figure S20. It makes clear the range of $\{K_{12}^0, \theta\}$ values for which a significant rectification of the luminescence signal of the probe P can be observed: K_{12}^0 should typically range between 10^{-2} and 10^0 whereas θ should be lower than 10^0.

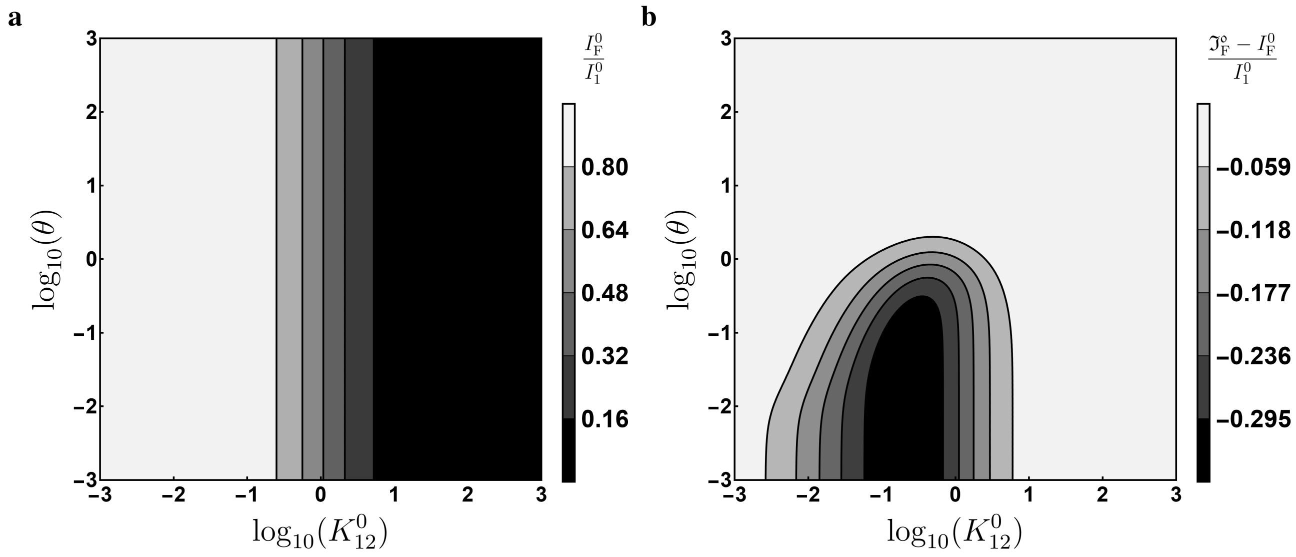

Figure S20: *Dependence of the normalized values of I_F^0 (**a**) and $\mathfrak{I}_F^{\circ} - I_F^0$ (**b**) on the dimensionless angular frequency θ and on the constant K_{12}^0 for a luminophore ($P_{\rm tot} = 1$ M, $Q_{1,1} = 1$, $Q_{1,2} = 0$, $Q_{2,1} = 0$, and $Q_{2,2} = 0$) submitted to the reaction (1) under sinusoidal modulation at a single angular frequency of modulation at two wavelengths with $\alpha = 1$ and $\varphi = \pi$.*

A.2.3 Dimensionalized maps

The effective implementation of the rectification of the luminescence signal requires to dimensionalize the maps displayed in Figure S20. We considered the case of reversibly photo-convertible fluorophores, which can be photoswitched at two wavelengths λ_1 and λ_2 governing the photoconversions from the state 1 to the state 2 and from the state 2 to the state 1 respectively. Figure S21a displays the dependence of the normalized luminescence $\mathfrak{I}_F^{\circ}$ on the angular frequency ω and on the light intensity I_2^0 for the reversibly photo-convertible fluorescent protein Dronpa-2, which is characterized by $\lambda_1 = 488$ nm and $\lambda_2 = 405$ nm, $\sigma_{12,1} = 198$ m^2.mol^{-1}, $\sigma_{21,1} = 0$ m^2.mol^{-1}, $\sigma_{12,2} = 0$ m^2.mol^{-1}, and $\sigma_{21,2} = 415$ m^2.mol^{-1}, and $k_{21}^{\Delta} = 1.4\ 10^{-2}$ s^{-1}.[5] Figure S21b further evaluates the dependence of the normalized difference $\mathfrak{I}_F^{\circ}\left(\omega = 10^{10}\right) - \mathfrak{I}_F^{\circ}\left(\omega = 10^{0}\right)$ on the light intensity I_2^0, which shows that it is possible to recover a significant rectification of the fluorescence signal of Dronpa-2 over a wide range of light intensity I_2^0. At the optimal I_2^0 value, $\mathfrak{I}_F^{\circ}$ increases by 34% when going from low to high frequency of light modulation.

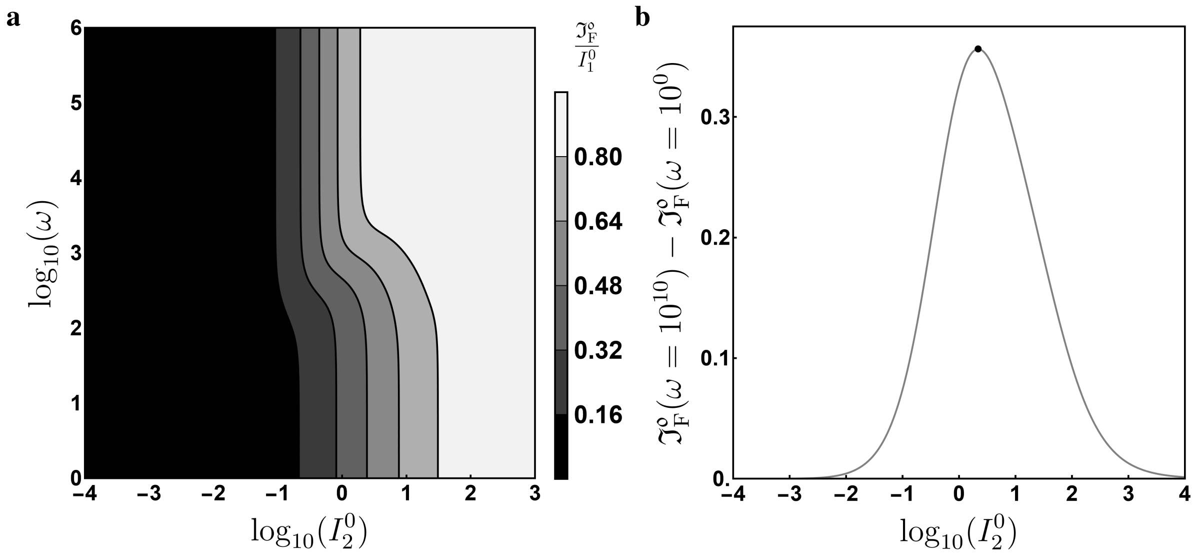

Figure S21: *Dependence of the normalized luminescence $\mathfrak{I}_F^o$ on the angular frequency ω and on the light intensity I_2^0 (**a**) and of the normalized difference $\mathfrak{I}_F^o\left(\omega = 10^{10}\right) - \mathfrak{I}_F^o\left(\omega = 10^0\right)$ on the light intensity I_2^0 (in Ein.m^{-2}.s^{-1}) (**b**) for Dronpa-2 (*$P_{\text{tot}} = 1$ M, $Q_{1,1} = 1$, $Q_{1,2} = 0$, $Q_{2,1} = 0$, $Q_{2,2} = 0$, $\sigma_{12,1} = 198$ m^2.mol^{-1}, $\sigma_{21,1} = 0$ m^2.mol^{-1}, $\sigma_{12,2} = 0$ m^2.mol^{-1}, $\sigma_{21,2} = 415$ m^2.mol^{-1}, *and* $k_{21}^{\Delta} = 2\ 10^{-2}$ s^{-1}*) submitted to the reaction (1) under sinusoidal modulation at a single angular frequency of modulation at two wavelengths with* $\alpha = 1$, $\varphi = \pi$, *and* $I_1^0 = 1$ Ein.m^{-2}.s^{-1}.

In order to retrieve the relaxation time τ_{12}^0, one has to process the dependence of $\mathfrak{I}_F^o$ on the angular frequency ω. To identify a relevant fitting function, we introduced the time dependence of the applied modulated illumination given in Eqs.(79–81) into the master differential equation given in Eq.(21) and retrieved analytic expressions of the time varying terms contained in Eqs.(49,53) upon truncating the Fourier expansion (30) at the 2nd order. Then we analyzed the mathematical structure of the resulting function and we decided to adopt the fitting function given in Eq.(86)

$$\mathcal{L}(\omega, p_1, p_2, p_3) = \frac{p1(1 + 4(\omega\tau_{12}^0)^2)}{p_2 + 5(\omega\tau_{12}^0)^2 + 4(\omega\tau_{12}^0)^4} + p_3 \tag{86}$$

in order to extract the relaxation time $\tau_{12}^{0,\text{fit}}$ from the computed dependence of the normalized luminescence $\mathfrak{I}_F^o$ of Dronpa-2 on the angular frequency ω for various values of the light intensity I_2^0. Figure S22a displays the results. Figure S22b displays the dependence of the fitted and expected relaxation times $\tau_{12}^{0,\text{fit}}$ and $\tau_{12}^{0,\text{th}}$ on the light intensity I_2^0. It shows that there is a satisfactory agreement over the $[10^{-1.7}; 10^1]$ range of light intensity I_2^0 expressed in Ein.m^{-2}.s^{-1}.

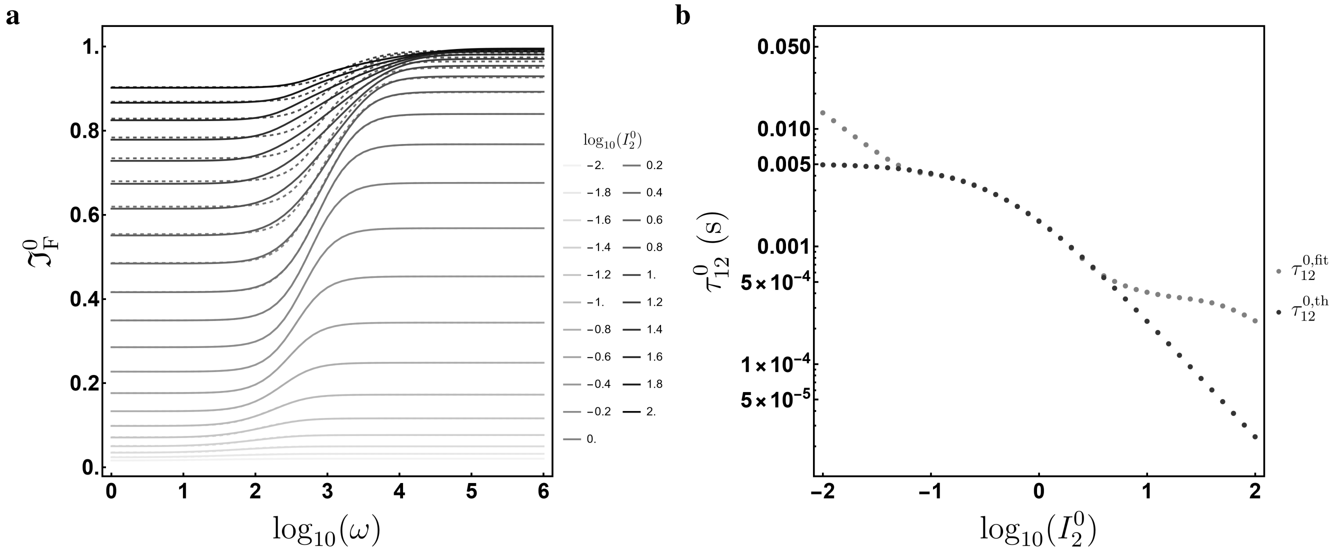

Figure S22: *Retrieval of the relaxation time τ_{12^0} from processing the dependence of $\mathfrak{I}_{\rm F}^{\rm o}$ on the angular frequency ω.* **a**: Dependence of the normalized luminescence $\mathfrak{I}_{\rm F}^{\rm o}$ on the angular frequency ω for various values of the light intensity I_2^0. Solid line: Numerical computation; Dashed line: Fit with Eq.86; **b**: Dependence of the fitted and expected relaxation times $\tau_{12}^{0,{\rm fit}}$ and $\tau_{12}^{0,{\rm th}}$ on the light intensity I_2^0 (in Ein.m^{-2}.s^{-1}) (**b**). Luminophore: Dronpa-2 ($P_{\rm tot} = 1$ M, $Q_{1,1} = 1$, $Q_{1,2} = 0$, $Q_{2,1} = 0$, $Q_{2,2} = 0$, $\sigma_{12,1} = 198$ m^2.mol^{-1}, $\sigma_{21,1} = 0$ m^2.mol^{-1}, $\sigma_{12,2} = 0$ m^2.mol^{-1}, $\sigma_{21,2} = 415$ m^2.mol^{-1}, and $k_{21}^{\Delta} = 1.4\,10^{-2}$ s^{-1}) submitted to the reaction (1) under sinusoidal modulation at a single angular frequency of modulation at two wavelengths with $\alpha = 1$, $\varphi = \pi$, and $I_1^0 = 1$ Ein.m^{-2}.s^{-1}.

B Square-wave modulation at a single angular frequency

In relation to the RIOM protocol, we now consider for $I(t)$ a square-wave illumination modulated at the fundamental angular frequency of modulation ω and involving the superposition of two lights at wavelengths λ_1 (around the averaged value I_1^0) and λ_2 (around the averaged value I_2^0). We adopted the general expression, which is given in Eq.(87):

$$I(t) = I_1^0\left[1 + \alpha h_1(t)\right] + I_2^0\left[1 + \alpha\delta h_2(t)\right] \quad (87)$$

$$h_1(t) = \frac{4}{\pi}\sum_{p=0}^{3}\frac{1}{2\times p + 1}\sin[(2\times p + 1)\theta x] \quad (88)$$

$$h_2(t) = -h_1(t) \quad (89)$$

It is applied on a luminophore for which the components of illumination $I_1(t)$ and $I_2(t)$ preferentially promote the photoconversion of the state **1** to the state **2**, and the one of the state **2** to the state **1** respectively.

B.1 Light modulation at a single wavelength

As in the case of the sinusoidal modulation, we first examined the case of light modulation at a single wavelength λ_1 (around the averaged value I_1^0) ($\delta = 0$ in Eq.(87)) (Figure S23).

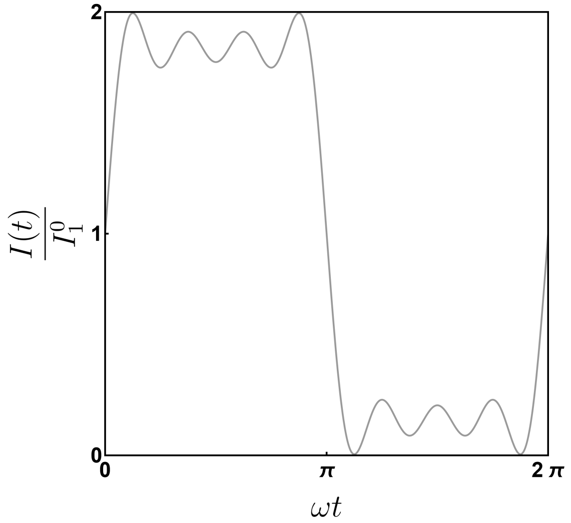

Figure S23: *Square-wave illumination involving light at wavelength λ_1 modulated around the averaged value I_1^0 at the fundamental angular frequency of modulation ω according to Eq. (87) with $\alpha = 0.84$ and $\delta = 0$.*

B.1.1 Generic maps

Following the general derivation given in subsection 1.2.1, we analytically retrieved the $2n+1$ unknown terms (a^0,...,a^n,b^n) upon truncating the Fourier expansion (30) at the 7th order. We then established the map of the deviation of the rectification of

- $\mathfrak{2}^{\mathrm{o}}$ with respect to the luminescence signal 2^0 obtained under constant illumination to address the case of the behavior of the phosphorescent probes in RIOM;

- $\mathfrak{I}_{\mathrm{F}}^{\mathrm{o}}$ with respect to the luminescence signal I_{F}^0 obtained under constant illumination given in Eq.(90)

$$I_{\mathrm{F}}^0 = \left(Q_{1,1}1^0 + Q_{2,1}2^0\right) I_1^0 + \left(Q_{1,2}1^0 + Q_{2,2}2^0\right) I_2^0 \tag{90}$$

  to address the case of the behavior of the other presently considered fluorescent probes in RIOM.

The result is displayed in Figure S24. It makes clear the range of $\{K_{12}^0, \theta\}$ values for which a significant rectification of the luminescence signal of the probe P can be observed: K_{12}^0 should typically range between 10^{-1} and 10^2 whereas θ should be lower than 10^0 for the phosphorescent probes and K_{12}^0 should typically range between 10^{-2} and 10^1 whereas θ should be lower than 10^0 for the fluorescent probes.

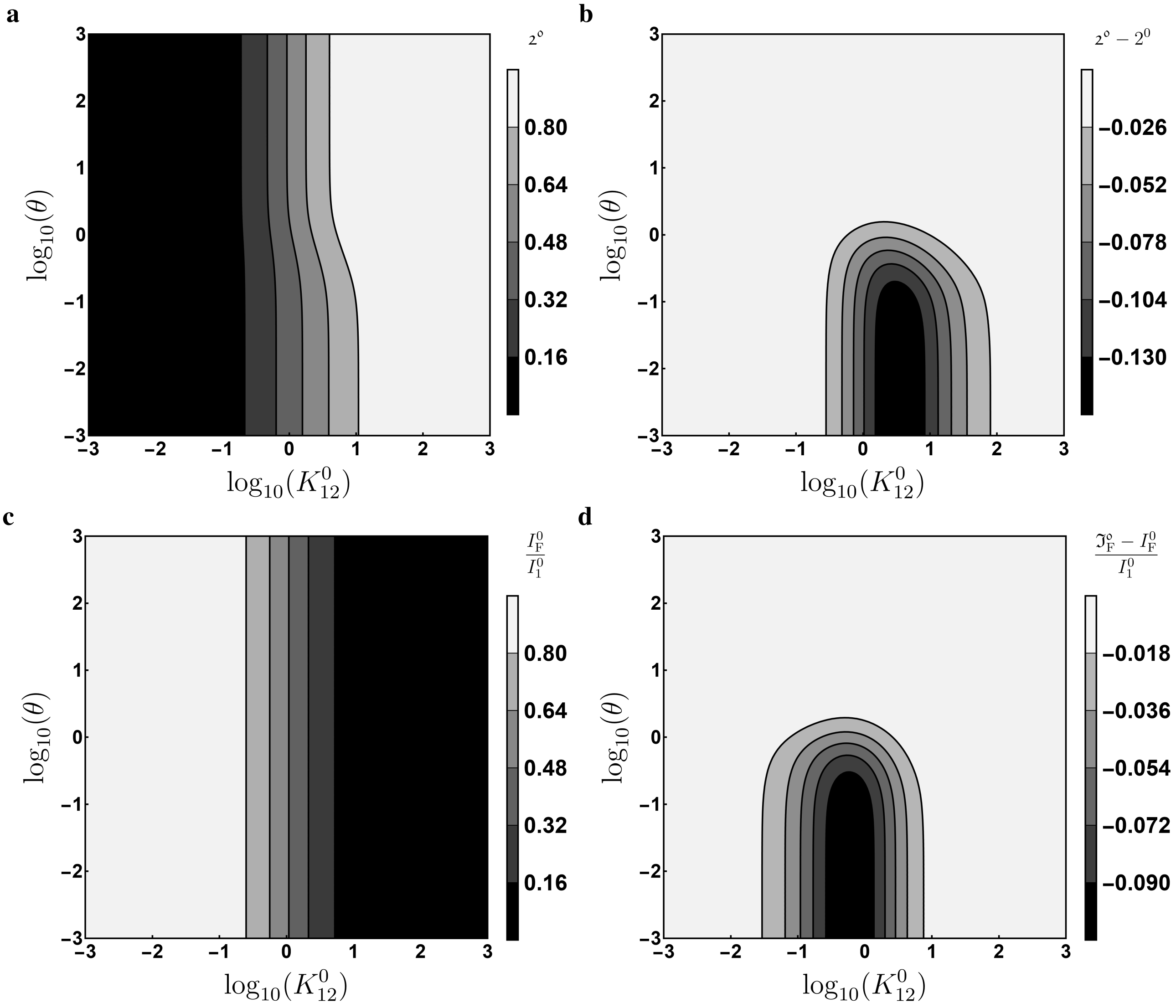

Figure S24: *Dependence of the normalized values of $\mathfrak{z}^{\circ}$ (**a**) and $\mathfrak{z}^{\circ} - 2^0$ (**b**), and I_{F}^0 (**c**) and $\mathfrak{I}_{\mathrm{F}}^{\circ} - I_{\mathrm{F}}^0$ (**d**) on the dimensionless angular frequency θ and on the constant K_{12}^0 for a luminophore ($P_{\mathrm{tot}} = 1$ M, and $Q_{1,1} = 1$, $Q_{1,2} = 0$, $Q_{2,1} = 0$, and $Q_{2,2} = 0$ in **c** and **d**) submitted to the reaction (1) under square-wave modulation at a single angular frequency of modulation at the wavelength λ_1 with $\alpha = 0.84$.*

B.1.2 Dimensionalized maps

The effective implementation of the rectification of the luminescence signal requires to dimensionalize the maps displayed in Figure S20.

B.1.2.1 Phosphorescent probes We first considered the case of phosphorescent probes, which can be photoactivated at the wavelength λ_1 governing the photoconversions from the state 1 to the state 2. Figure S25a displays the dependence of the normalized phosphorescence $\mathfrak{z}^{\circ}$ on the angular frequency ω and on the light intensity I_1^0 for a phosphorescent probe, which is characterized by $\lambda_1 = 405$ nm, $\sigma_{12,1} = 50$ m^2.mol^{-1}, $\sigma_{21,1} = 0$ m^2.mol^{-1}, and $k_{21}^{\Delta} = 2\ 10^5$ s^{-1}. Figure

S25b further evaluates the dependence of the normalized difference $\mathfrak{z}^{0}\left(\omega=10^{10}\right)-\mathfrak{z}^{0}\left(\omega=10^{0}\right)$ on the light intensity I_1^0, which shows that it is possible to recover a significant rectification of the phosphorescence signal of the probe over a wide range of light intensity I_1^0. At the optimal I_1^0 value, $\mathfrak{z}^{0}$ increases by 16% when going from low to high frequency of light modulation.

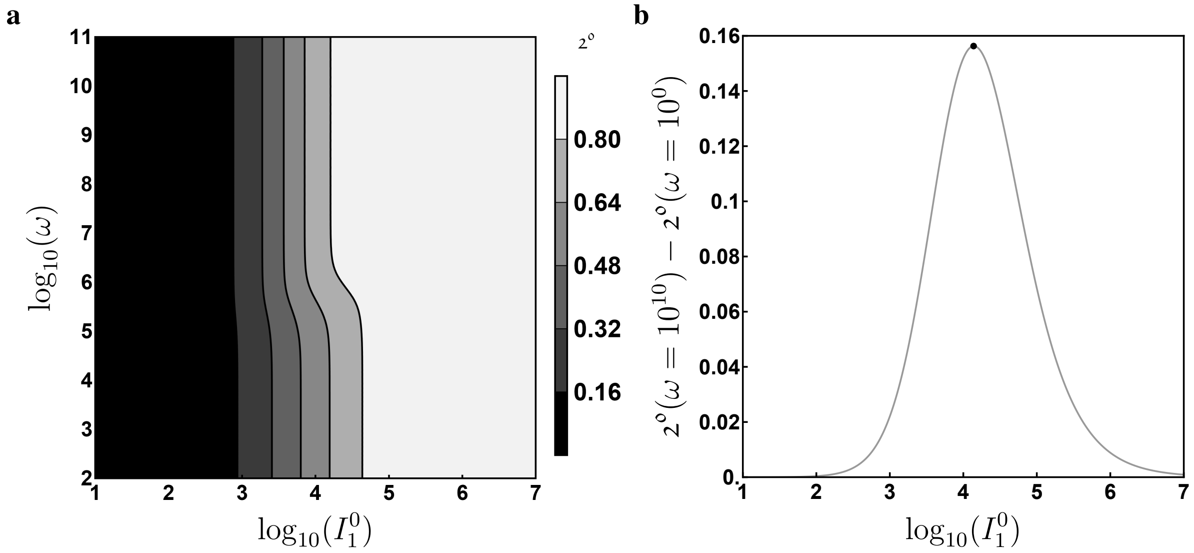

Figure S25: *Dependence of the normalized phosphorescence $\mathfrak{z}^{0}$ on the angular frequency ω and on the light intensity I_1^0 (**a**) and of the normalized difference $\mathfrak{z}^{0}\left(\omega=10^{10}\right)-\mathfrak{z}^{0}\left(\omega=10^{0}\right)$ on the light intensity I_1^0 (in* Ein.m^{-2}.s^{-1}*) (**b**) for a phosphorescent probe ($P_{\rm tot}=1$ M, $\sigma_{12,1}=50$ m^2.mol^{-1}, $\sigma_{21,1}=0$ m^2.mol^{-1}, and $k_{21}^{\Delta}=2\ 10^5$ s^{-1} submitted to the reaction (1) under square-wave modulation at a single angular frequency of modulation at the wavelength λ_1 with* $\alpha=0.84$.

In order to retrieve the relaxation time τ_{12}^0, one has to process the dependence of $\mathfrak{z}^{0}$ on the angular frequency ω. To identify a relevant fitting function, we introduced the time dependence of the applied modulated illumination given in Eqs.(79–81) into the master differential equation given in Eq.(21) and retrieved analytic expressions of the time varying terms contained in Eq.(37) upon truncating the Fourier expansion (30) at the 2$^{\rm nd}$ order. Then we analyzed the mathematical structure of the resulting function and we decided to adopt the fitting function given in Eq.(84)

$$\mathcal{L}(\omega,p_1,p_2,p_3)=\frac{p1(1+4(\omega\tau_{12}^0)^2)}{p_2+5(\omega\tau_{12}^0)^2+4(\omega\tau_{12}^0)^4}+p_3 \tag{91}$$

in order to extract the relaxation time $\tau_{12}^{0,\rm fit}$ from the computed dependence of the normalized luminescence $\mathfrak{z}^{0}$ of the phosphorescent probe on the angular frequency ω for various values of the light intensity I_1^0. Figure S26a displays the results. Figure S26b displays the dependence of the fitted and expected relaxation times $\tau_{12}^{0,\rm fit}$ and $\tau_{12}^{0,\rm th}$ on the light intensity I_1^0. It shows that there is a satisfactory agreement over the $[10^{2.5};10^{6.5}]$ range of light intensity I_1^0 expressed in Ein.m^{-2}.s^{-1}.

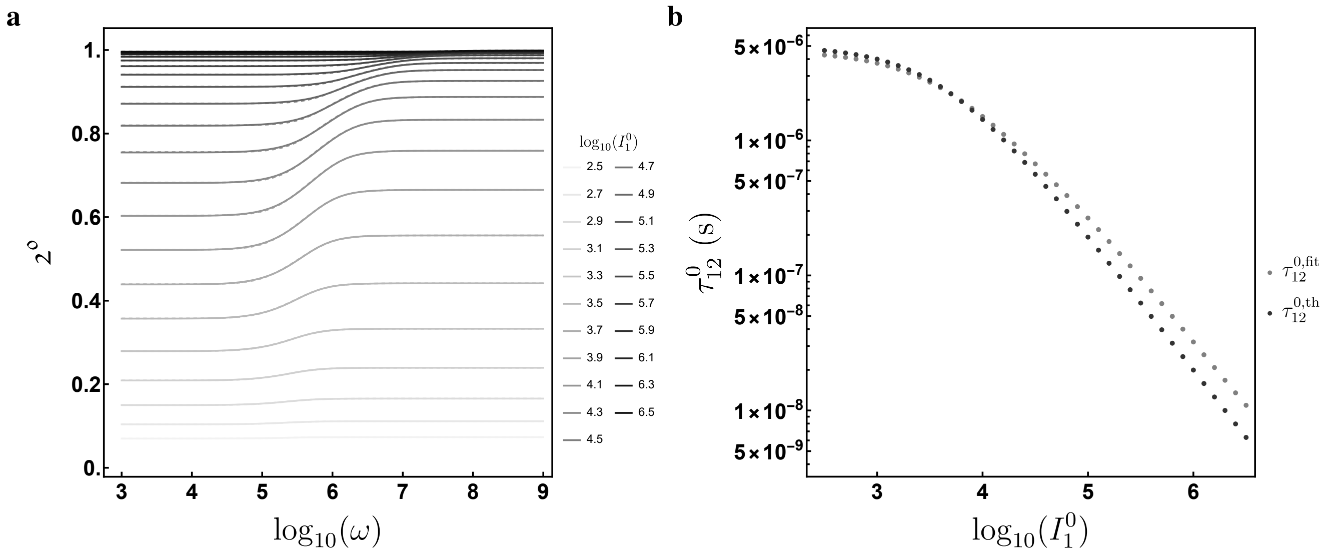

Figure S26: *Retrieval of the relaxation time τ_{12}^{0} from processing the dependence of $\mathfrak{I}^{o}$ on the angular frequency ω.* **a**: Dependence of the normalized luminescence $\mathfrak{I}^{o}$ on the angular frequency ω for various values of the light intensity I_1^0. Solid line: Numerical computation; Dashed line: Fit with Eq.(83); **b**: Dependence of the fitted and expected relaxation times $\tau_{12}^{0,\mathrm{fit}}$ and $\tau_{12}^{0,\mathrm{th}}$ on the light intensity I_1^0 (in Ein.m^{-2}.s^{-1}). Luminophore: Phosphorescent probe ($P_{\mathrm{tot}} = 1$, $Q_{1,1} = 1$, $Q_{1,2} = 0$, $\sigma_{12,1} = 50$ m^2.mol^{-1}, $\sigma_{21,1} = 0$ m^2.mol^{-1}, and $k_{21}^{\Delta} = 2\ 10^5$ s^{-1}) submitted to the reaction (1) under square-wave modulation at a single angular frequency of modulation at the wavelength λ_1 with $\alpha = 0.84$.

B.1.2.2 Reversibly photo-convertible fluorophores We then considered the case of reversibly photo-convertible fluorophores, which can be photoswitched at two wavelengths λ_1 and λ_2 governing the photoconversions from the state 1 to the state 2 and from the state 2 to the state 1 respectively. Figure S27a displays the dependence of the normalized luminescence $\mathfrak{I}_{\mathrm{F}}^{o}$ on the angular frequency ω and on the light intensity I_2^0 for the reversibly photo-convertible fluorescent protein Dronpa-2, which is characterized by $\lambda_1 = 488$ nm and $\lambda_2 = 405$ nm, $\sigma_{12,1} = 198$ m^2.mol^{-1}, $\sigma_{21,1} = 0$ m^2.mol^{-1}, $\sigma_{12,2} = 0$ m^2.mol^{-1}, and $\sigma_{21,2} = 415$ m^2.mol^{-1}, and $k_{21}^{\Delta} = 1.4\ 10^{-2}$ s^{-1}.[5] Figure S27b further evaluates the dependence of the normalized difference $\mathfrak{I}_{\mathrm{F}}^{o}\left(\omega = 10^{10}\right) - \mathfrak{I}_{\mathrm{F}}^{o}\left(\omega = 10^{0}\right)$ on the light intensity I_2^0, which shows that it is possible to recover a significant rectification of the fluorescence signal of Dronpa-2 over a wide range of light intensity I_2^0. At the optimal I_2^0 value, $\mathfrak{I}_{\mathrm{F}}^{o}$ increases by 11% when going from low to high frequency of light modulation.

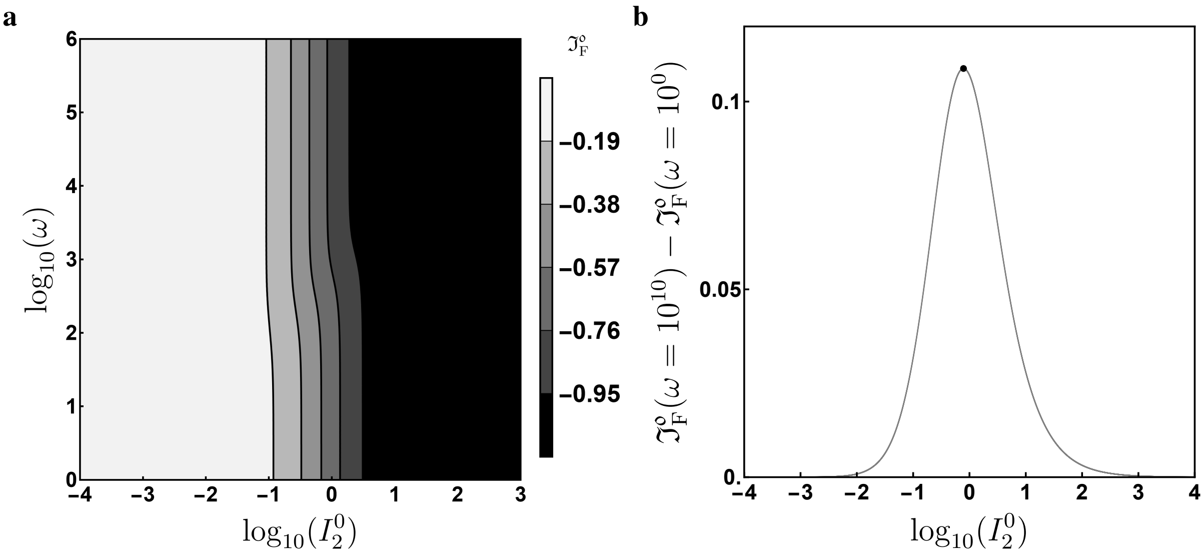

Figure S27: *Dependence of the normalized luminescence* $\mathfrak{I}_{\rm F}^{\rm o}$ *on the angular frequency* ω *and on the light intensity* I_2^0 *(**a**) and of the normalized difference* $\mathfrak{I}_{\rm F}^{\rm o}\left(\omega = 10^{10}\right) - \mathfrak{I}_{\rm F}^{\rm o}\left(\omega = 10^{0}\right)$ *on the light intensity* I_2^0 *(in Ein.m*$^{-2}$*.s*$^{-1}$*) (**b**) for Dronpa-2 (*$P_{\rm tot} = 1$ M, $Q_{1,1} = 1$, $Q_{1,2} = 0$, $Q_{2,1} = 0$, $Q_{2,2} = 0$, $\sigma_{12,1} = 198$ m^2.mol^{-1}, $\sigma_{21,1} = 0$ m^2.mol^{-1}, $\sigma_{12,2} = 0$ m^2.mol^{-1}, $\sigma_{21,2} = 415$ m^2.mol^{-1}, *and* $k_{21}^{\Delta} = 1.4\ 10^{-2}$ s^{-1}*) submitted to the reaction (1) under square-wave modulation at a single angular frequency of modulation at the wavelength* λ_1 *with* $\alpha = 0.84$ *and* $I_1^0 = 1$ *Ein.m*$^{-2}$*.s*$^{-1}$*.*

In order to retrieve the relaxation time τ_{12}^0, one has to process the dependence of $\mathfrak{I}_{\rm F}^{\rm o}$ on the angular frequency ω. To identify a relevant fitting function, we introduced the time dependence of the applied modulated illumination given in Eqs.(79–81) into the master differential equation given in Eq.(21) and retrieved analytic expressions of the time varying terms contained in Eqs.(49,53) upon truncating the Fourier expansion (30) at the 2$^{\rm nd}$ order. Then we analyzed the mathematical structure of the resulting function and we decided to adopt the fitting function given in Eq.(92)

$$\mathcal{L}(\omega, p_1, p_2, p_3) = \frac{p1(1 + 4(\omega\tau_{12}^0)^2)}{p_2 + 5(\omega\tau_{12}^0)^2 + 4(\omega\tau_{12}^0)^4} + p_3 \tag{92}$$

in order to extract the relaxation time $\tau_{12}^{0,\rm fit}$ from the computed dependence of the normalized luminescence $\mathfrak{I}_{\rm F}^{\rm o}$ of Dronpa-2 on the angular frequency ω for various values of the light intensity I_2^0. Figure S28a displays the results. Figure S28b displays the dependence of the fitted and expected relaxation times $\tau_{12}^{0,\rm fit}$ and $\tau_{12}^{0,\rm th}$ on the light intensity I_2^0. It shows that there is a satisfactory agreement over the $[10^{-2}; 10^{2}]$ range of light intensity I_2^0 expressed in Ein.m^{-2}.s^{-1}.

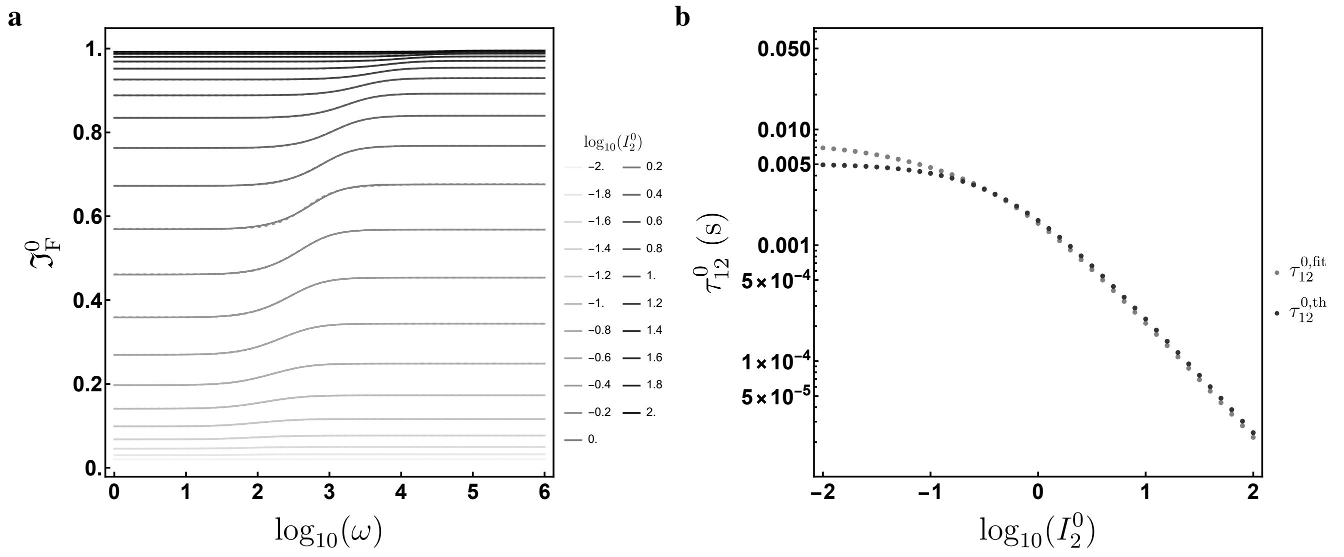

Figure S28: *Retrieval of the relaxation time* τ_{12^0} *from processing the dependence of* $\mathfrak{I}_F^o$ *on the angular frequency* ω. **a**: Dependence of the normalized luminescence $\mathfrak{I}_F^o$ on the angular frequency ω for various values of the light intensity I_2^0. Solid line: Numerical computation; Dashed line: Fit with Eq.(92); **b**: Dependence of the fitted and expected relaxation times $\tau_{12}^{0,\mathrm{fit}}$ and $\tau_{12}^{0,\mathrm{th}}$ on the light intensity I_2^0 (in Ein.m^{-2}.s^{-1}). Luminophore: Dronpa-2 ($P_{\mathrm{tot}} = 1$ M, $Q_{1,1} = 1$, $Q_{1,2} = 0$, $Q_{2,1} = 0$, $Q_{2,2} = 0$, $\sigma_{21,1} = 0$ m^2.mol^{-1}, $\sigma_{12,2} = 0$ m^2.mol^{-1}, $\sigma_{21,2} = 415$ m^2.mol^{-1}, and $k_{21}^{\Delta} = 1.4\ 10^{-2}$ s^{-1}) submitted to the reaction (1) under square-wave modulation at a single angular frequency of modulation at the wavelength λ_1 with $\alpha = 0.84$ and $I_1^0 = 1$ Ein.m^{-2}.s^{-1}.

B.2 Light modulation at two wavelengths

In relation to the RIOM protocol on fluorophores reversibly photo-convertible at two distinct wavelength (e.g. Dronpa-2), we then examined for $I(t)$ the superposition of two antiphase-related square-wave light modulations of large amplitude at angular frequency ω at wavelengths λ_1 (around the averaged value I_1^0) and at λ_2 (around the averaged value I_2^0) (Figure S29).

B Square-wave modulation at a single angular frequency

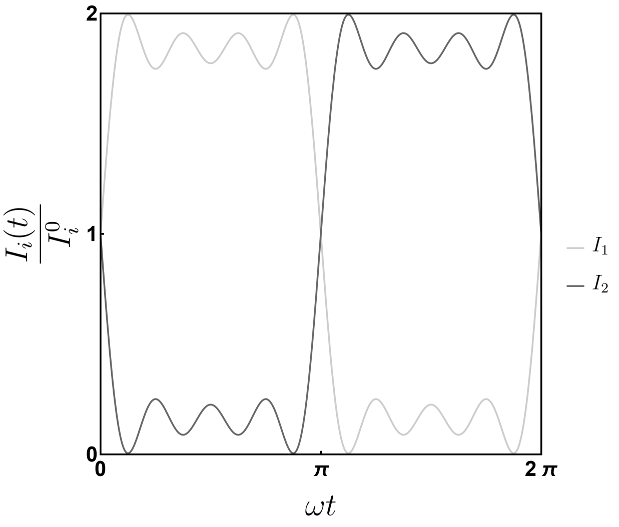

Figure S29: *Antiphase-related square-wave light modulation of large amplitude at angular frequency ω at wavelengths λ_1 (around the averaged value I_1^0) and λ_2 (around the averaged value I_2^0) with $\alpha = 0.84$ and $\delta = 1$.*

B.2.1 Generic maps

We first established the map of the deviation of the rectification of $\mathfrak{I}_{\mathrm{F}}^{\circ}$ with respect to the luminescence signal I_{F}^0 obtained under constant illumination given in Eq.(90). The result is displayed in Figure S30. It makes clear the range of $\{K_{12}^0, \theta\}$ values for which a significant rectification of the luminescence signal of the probe P can be observed: K_{12}^0 should typically range between 10^{-2} and 10^1 whereas θ should be lower than 10^0.

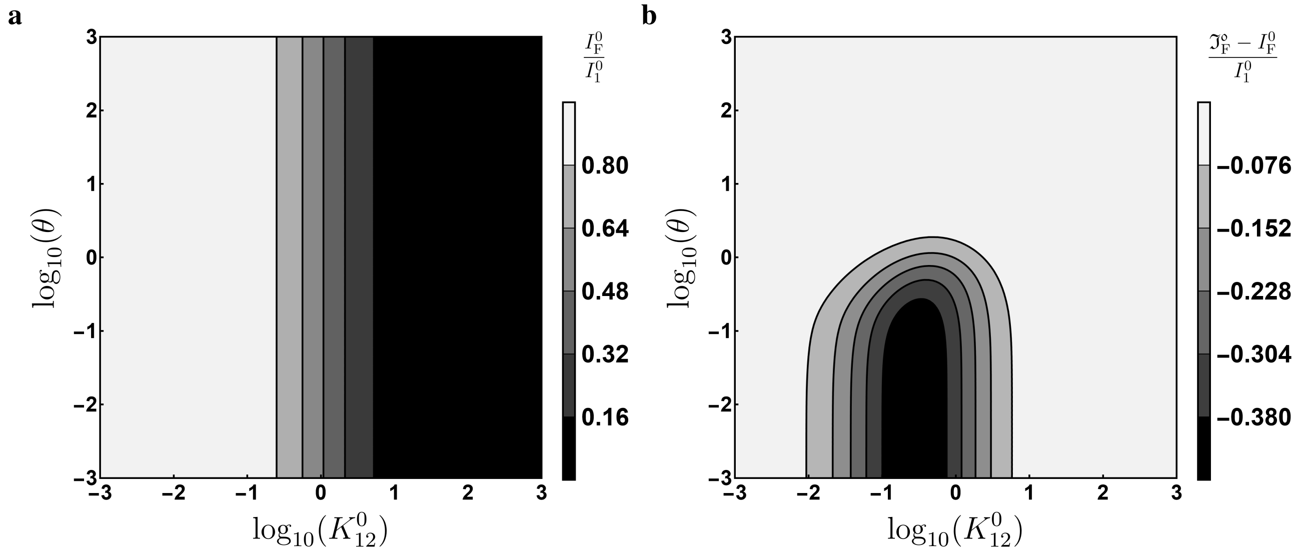

Figure S30: *Dependence of the normalized values of I_{F}^0 (**a**) and $\mathfrak{I}_{\mathrm{F}}^{\circ} - I_{\mathrm{F}}^0$ (**b**) on the dimensionless angular frequency θ and on the constant K_{12}^0 for a luminophore ($P_{\mathrm{tot}} = 1$ M, $Q_{1,1} = 1$, $Q_{1,2} = 0$, $Q_{2,1} = 0$, and $Q_{2,2} = 0$) submitted to the reaction (1) under antiphase-related square-wave light modulation at wavelengths λ_1 and λ_2 with $\alpha = 0.84$.*

B.2.2 Dimensionalized maps

The effective implementation of the rectification of the luminescence signal requires to dimensionalize the maps displayed in Figure S20. We considered the case of reversibly photo-convertible fluorophores, which can be photoswitched at two wavelengths λ_1 and λ_2 governing the photoconversions from the state 1 to the state 2 and from the state 2 to the state 1 respectively. Figure S31a displays the dependence of the normalized luminescence $\mathfrak{I}_{\mathrm{F}}^{\mathrm{o}}$ on the angular frequency ω and on the light intensity I_2^0 for the reversibly photo-convertible fluorescent protein Dronpa-2, which is characterized by $\lambda_1 = 488$ nm and $\lambda_2 = 405$ nm, $\sigma_{12,1} = 198$ m^2.mol^{-1}, $\sigma_{21,1} = 0$ m^2.mol^{-1}, $\sigma_{12,2} = 0$ m^2.mol^{-1}, and $\sigma_{21,2} = 415$ m^2.mol^{-1}, and $k_{21}^{\Delta} = 1.4\ 10^{-2}$ s^{-1}.[5] Figure S31b further evaluates the dependence of the normalized difference $\mathfrak{I}_{\mathrm{F}}^{\mathrm{o}}\left(\omega = 10^{10}\right) - \mathfrak{I}_{\mathrm{F}}^{\mathrm{o}}\left(\omega = 10^{0}\right)$ on the light intensity I_2^0, which shows that it is possible to recover a significant rectification of the fluorescence signal of Dronpa-2 over a wide range of light intensity I_2^0. At the optimal I_2^0 value, $\mathfrak{I}_{\mathrm{F}}^{\mathrm{o}}$ increases by 46% when going from low to high frequency of light modulation.

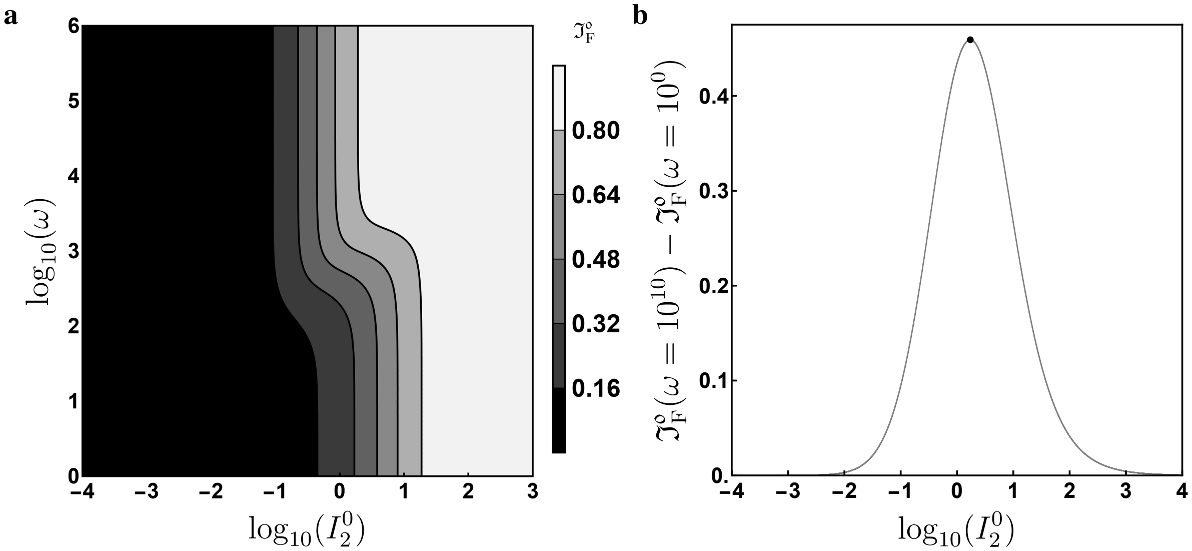

Figure S31: *Dependence of the normalized luminescence* $\mathfrak{I}_{\mathrm{F}}^{\mathrm{o}}$ *on the angular frequency* ω *and on the light intensity* I_2^0 (***a***) *and of the normalized difference* $\mathfrak{I}_{\mathrm{F}}^{\mathrm{o}}\left(\omega = 10^{10}\right) - \mathfrak{I}_{\mathrm{F}}^{\mathrm{o}}\left(\omega = 10^{0}\right)$ *on the light intensity* I_2^0 *(in Ein.m*$^{-2}$*.s*$^{-1}$*)* (***b***) *for Dronpa-2 (*$P_{\mathrm{tot}} = 1$ M, $Q_{1,1} = 1$, $Q_{1,2} = 0$, $Q_{2,1} = 0$, $Q_{2,2} = 0$, $\sigma_{12,1} = 198$ m^2.mol^{-1}, $\sigma_{21,1} = 0$ m^2.mol^{-1}, $\sigma_{12,2} = 0$ m^2.mol^{-1}, $\sigma_{21,2} = 415$ m^2.mol^{-1}, *and* $k_{21}^{\Delta} = 1.4\ 10^{-2}$ s^{-1}*) submitted to the reaction (1) under antiphase-related square-wave light modulation at wavelengths* λ_1 *and* λ_2 *with* $\alpha = 0.84$ *and* $I_1^0 = 1$ *Ein.m*$^{-2}$*.s*$^{-1}$.

In order to retrieve the relaxation time τ_{12}^0, one has to process the dependence of $\mathfrak{I}_{\mathrm{F}}^{\mathrm{o}}$ on the angular frequency ω. To identify a relevant fitting function, we introduced the time dependence of the applied modulated illumination given in Eqs.(79–81) into the master differential equation given in Eq.(21) and retrieved analytic expressions of the time varying terms contained in Eqs.(49,53) upon truncating the Fourier expansion (30) at the 2nd order. Then we analyzed the mathematical structure of the resulting function and we decided to adopt the fitting function given in Eq.(93)

$$\mathcal{L}(\omega, p_1, p_2, p_3) = \frac{p1(1 + 4(\omega\tau_{12}^0)^2)}{p_2 + 5(\omega\tau_{12}^0)^2 + 4(\omega\tau_{12}^0)^4} + p_3 \tag{93}$$

in order to extract the relaxation time $\tau_{12}^{0,\mathrm{fit}}$ from the computed dependence of the normalized luminescence $\mathfrak{I}_\mathrm{F}^o$ of Dronpa-2 on the angular frequency ω for various values of the light intensity I_2^0. Figure S32a displays the results. Figure S32b displays the dependence of the fitted and expected relaxation times $\tau_{12}^{0,\mathrm{fit}}$ and $\tau_{12}^{0,\mathrm{th}}$ on the light intensity I_2^0. It shows that there is a satisfactory agreement over the $[10^{-2}; 10^2]$ range of light intensity I_2^0 expressed in Ein.m^{-2}.s^{-1}.

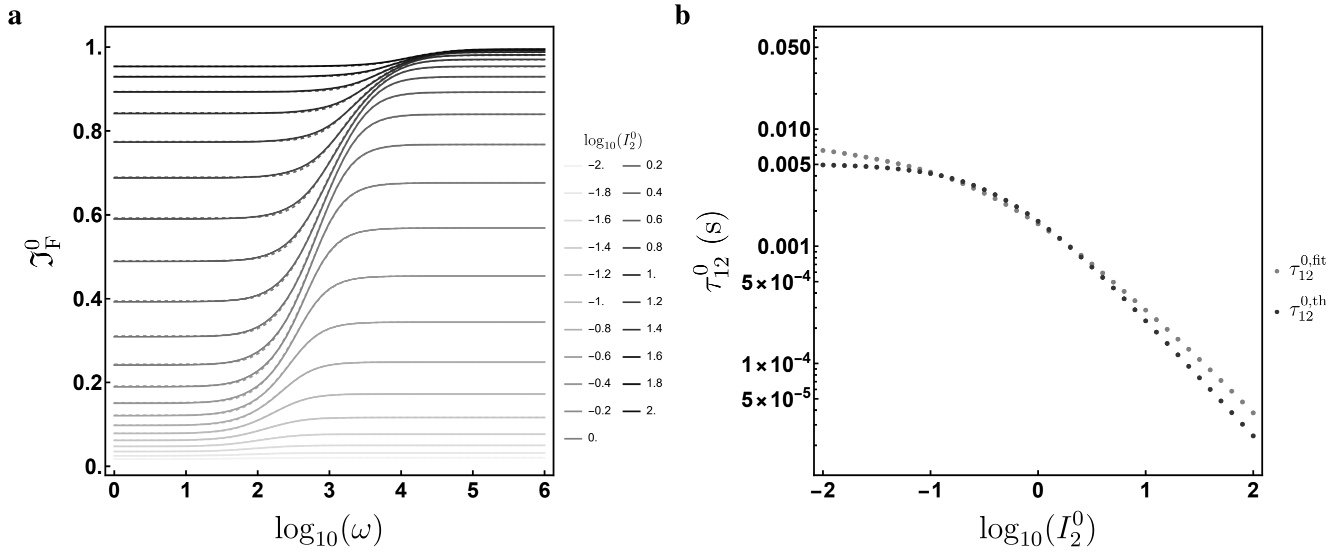

Figure S32: *Retrieval of the relaxation time τ_{12^0} from processing the dependence of $\mathfrak{I}_\mathrm{F}^o$ on the angular frequency ω.* **a**: Dependence of the normalized luminescence $\mathfrak{I}_\mathrm{F}^o$ on the angular frequency ω for various values of the light intensity I_2^0. Solid line: Numerical computation; Dashed line: Fit with Eq.(93); **b**: Dependence of the fitted and expected relaxation times $\tau_{12}^{0,\mathrm{fit}}$ and $\tau_{12}^{0,\mathrm{th}}$ on the light intensity I_2^0 (in Ein.m^{-2}.s^{-1}). Luminophore: Dronpa-2 ($P_\mathrm{tot} = 1$ m, $Q_{1,1} = 1$, $Q_{1,2} = 0$, $Q_{2,1} = 0$, $Q_{2,2} = 0$, $\sigma_{12,1} = 198$ m^2.mol^{-1}, $\sigma_{21,1} = 0$ m^2.mol^{-1}, $\sigma_{12,2} = 0$ m^2.mol^{-1}, $\sigma_{21,2} = 415$ m^2.mol^{-1}, and $k_{21}^\Delta = 1.4\ 10^{-2}$ s^{-1}) submitted to the reaction (1) under antiphase-related square-wave light modulation at wavelengths λ_1 and λ_2 with $\alpha = 0.84$ and $I_1^0 = 1$ Ein.m^{-2}.s^{-1}.

C Sinusoidal modulation at two angular frequencies

In relation to the HIOM protocol, we first considered for $I(t)$ an illumination involving the superposition of two lights at wavelengths λ_1 (sinusoidally modulated at angular frequencies ω_1 around the averaged value I_1^0) and λ_2 (sinusoidally modulated at angular frequencies ω_2 around the averaged value I_2^0). We adopted the expression, which is given in Eq.(94):

$$I(t) = I_1^0\,[1+\alpha h_1(t)] + I_2^0\,[1+\alpha h_2(t)] \tag{94}$$

$$h_1(t) = \sin(\omega_1 t) \tag{95}$$

$$h_2(t) = \sin(\omega_2 t + \varphi) \tag{96}$$

C.1 Significance of the phase lag φ

We here addressed how the phase lag φ affects the dependence of:

- $\mathfrak{z}^{1,-1,\mathfrak{sin}}$ and $\mathfrak{z}^{1,-1,\mathfrak{cos}}$ on the dimensionless angular frequency θ_1 (at constant $\Delta\theta = \theta_2 - \theta_1 = \pi\tau_{12}^0$) and constant K_{12}^0 when both components of illumination $I_1(t)$ and $I_2(t)$ promote the photoconversion of the state **1** to the state **2** (case of the phosphorescent probes). The results are displayed in Figure S33. $\mathfrak{z}^{1,-1}$ strongly depends on the dimensionless angular frequency θ_1 at constant K_{12}^0. The most favorable terms are $\mathfrak{z}^{1,-1,\mathfrak{sin}}$ when $\varphi = \frac{\pi}{2}$ and $\frac{3\pi}{2}$, and $\mathfrak{z}^{1,-1,\mathfrak{cos}}$ when $\varphi = 0$ and π. The behavior of both terms is similar and we retained $\mathfrak{z}^{1,-1,\mathfrak{cos}}$ with $\varphi = \pi$ in the following;

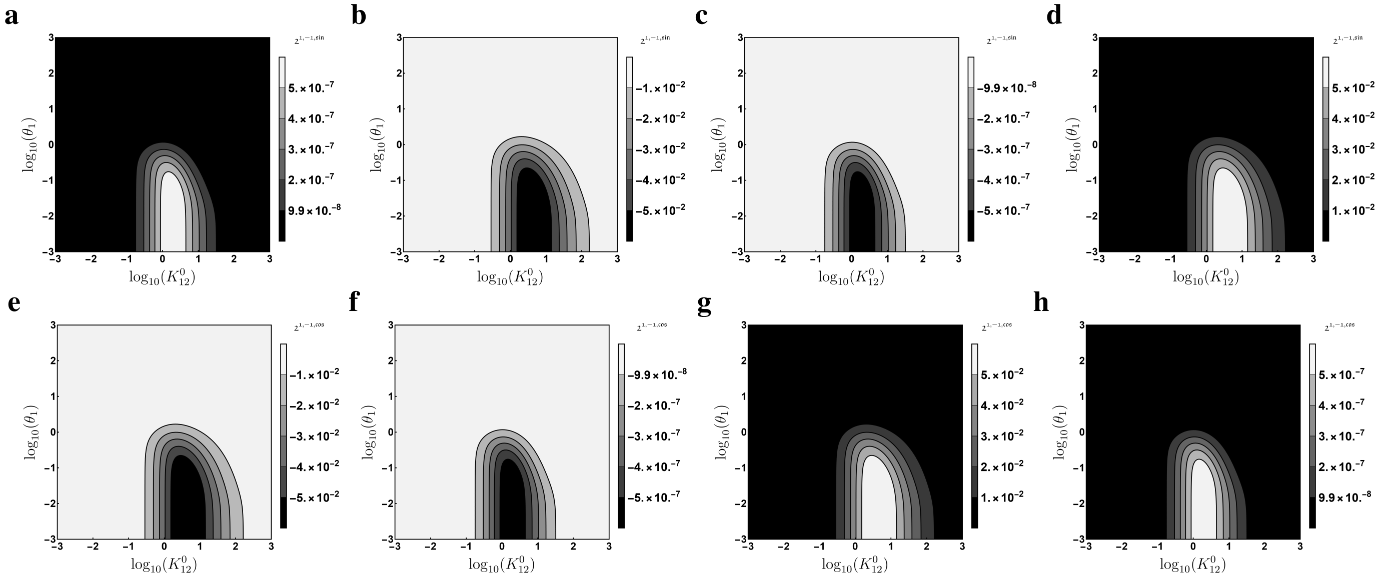

Figure S33: *Significance of the phase lag φ on the (θ_1,K_{12}^0)-dependent fluorescence signals $\mathfrak{z}^{1,-1,\mathfrak{sin}}$ (**a–d**) and $\mathfrak{z}^{1,-1,\mathfrak{cos}}$ (**e–h**) as computed with Eqs.(45,46) and the numerical solution of Eq.(21) truncated at the 5th order upon applying an illumination involving the superposition of two lights at wavelengths $\boldsymbol{\lambda}_1$ (sinusoidally modulated at angular frequencies ω_1 around the averaged value I_1^0) and $\boldsymbol{\lambda}_2$ (sinusoidally modulated at angular frequencies ω_2 around the averaged value I_2^0) with $\alpha = 1$ and $\Delta\theta = \theta_2 - \theta_1 = \pi\tau_{12}^0$ on a luminophore ($P_{\rm tot} = 1$ M) submitted to the reaction (1).* **a**,**e**: $\varphi = 0$; **b**,**f**: $\varphi = \frac{\pi}{2}$; **c**,**g**: $\varphi = \pi$; **d**,**h**: $\varphi = \frac{3\pi}{2}$.

- $\mathfrak{I}_{\rm F}^{1,-1,\mathfrak{sin}}$ and $\mathfrak{I}_{\rm F}^{1,-1,\mathfrak{cos}}$ on the dimensionless angular frequency θ_1 (at constant $\Delta\theta = \theta_2 - \theta_1 = \pi\tau_{12}^0$) and constant K_{12}^0 when the components of illumination $I_1(t)$ and $I_2(t)$ preferentially promote the photoconversion of the state **1** to the state **2**, and the one of the state **2** to the state **1** respectively (case of the reversibly photo-convertible fluorophores). The results are displayed in Figure S34. Both $\mathfrak{I}_{\rm F}^{1,-1,\mathfrak{sin}}$ and $\mathfrak{I}_{\rm F}^{1,-1,\mathfrak{cos}}$ strongly depend on the dimensionless angular frequency θ_1 at constant K_{12}^0. The most favorable terms are $\mathfrak{I}_{\rm F}^{1,-1,\mathfrak{sin}}$ when $\varphi = \frac{\pi}{2}$ and $\frac{3\pi}{2}$, and $\mathfrak{I}_{\rm F}^{1,-1,\mathfrak{cos}}$ when $\varphi = 0$ and π. The behavior of both terms is similar and we retained $\mathfrak{I}_{\rm F}^{1,-1,\mathfrak{cos}}$ with $\varphi = \pi$ in the following.

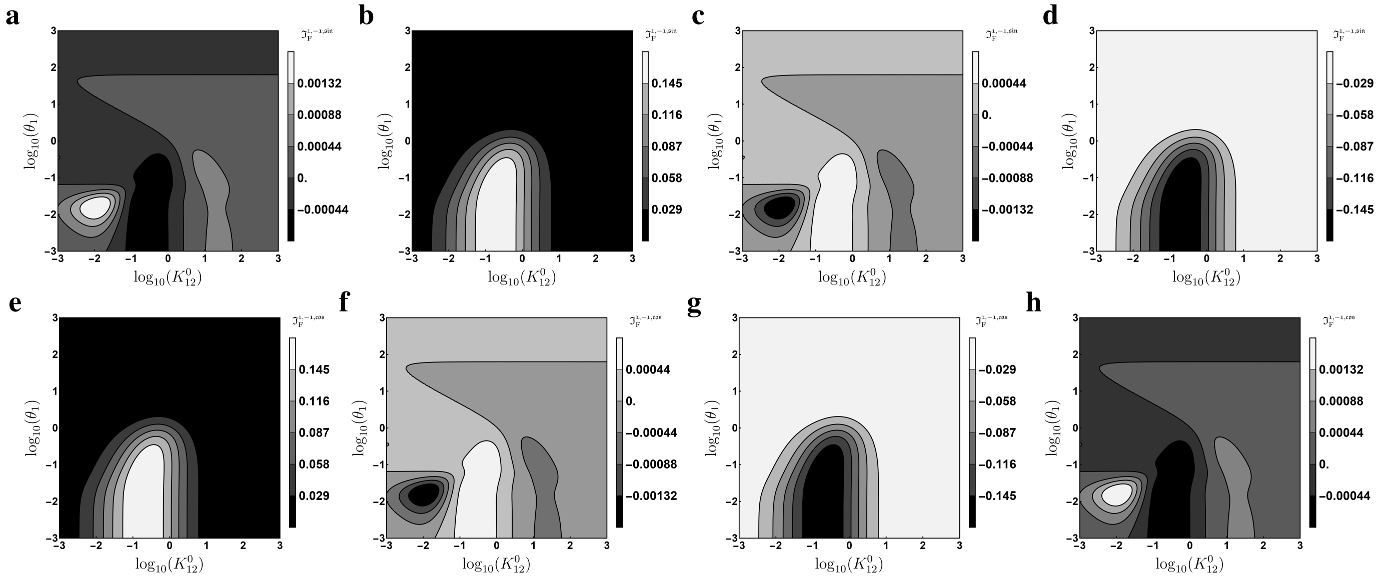

Figure S34: *Significance of the phase lag φ on the (θ_1,K_{12}^0)-dependent fluorescence signals $\mathfrak{I}_{\mathrm{F}}^{1,-1,\sin}$ (**a–d**) and $\mathfrak{I}_{\mathrm{F}}^{1,-1,\cos}$ (**e–h**) as computed with Eqs.(47–53) and the numerical solution of Eq.(21) truncated at the 5th order upon applying an illumination involving the superposition of two lights at wavelengths λ_1 (sinusoidally modulated at angular frequencies ω_1 around the averaged value I_1^0) and λ_2 (sinusoidally modulated at angular frequencies ω_2 around the averaged value I_2^0) with $\alpha = 1$ and $\Delta\theta = \theta_2 - \theta_1 = \pi\tau_{12}^0$ on a luminophore ($P_{\mathrm{tot}} = 1$ M, $Q_{1,1} = 1$, $Q_{1,2} = 0$, $Q_{2,1} = 0$, and $Q_{2,2} = 0$) submitted to the reaction (1).* **a**,**e**: $\varphi = 0$; **b**,**f**: $\varphi = \frac{\pi}{2}$; **c**,**g**: $\varphi = \pi$; **d**,**h**: $\varphi = \frac{3\pi}{2}$.

C.2 Generic maps

Following the general derivation given in subsection 1.2.1, we analytically retrieved the $2n+1$ unknown terms $(a^0,\ldots,a^n,b^n)$ upon truncating the Fourier expansion (30) at the 5th order. We then established the dependence on angular frequency ω_1 of

- $\mathfrak{z}^{1,-1,\cos}$ with $\varphi = \pi$ (to address the case of the behavior of the phosphorescent probes in HIOM);
- $\mathfrak{I}_{\mathrm{F}}^{1,-1,\cos}$ with $\varphi = \pi$ (to address the case of the behavior of the other presently considered fluorescent probes in HIOM)

to selectively discriminate a targeted luminophore and retrieve the relaxation time associated with its reaction (1). Thus, we further analyzed the amplitude of the variation of $\mathfrak{z}^{1,-1,\cos}$ and $\mathfrak{I}_{\mathrm{F}}^{1,-1,\cos}$ over the investigated range of dimensionless angular frequency θ_1 on the constant K_{12}^0 at constant $\Delta\theta = \theta_2 - \theta_1 = \pi\tau_{12}^0$. The result is displayed in Figure S35a,b. It makes clear the range of K_{12}^0 values for which a significant amplitude of the luminescence signal of the probe P can be observed: K_{12}^0 should typically range between 10^{-1} and $10^{2.5}$ whereas θ should be lower than 10^0 for the phosphorescent probes and K_{12}^0 should typically range between 10^{-3} and 10^1 whereas θ should be lower than 10^0 for the fluorescent probes.

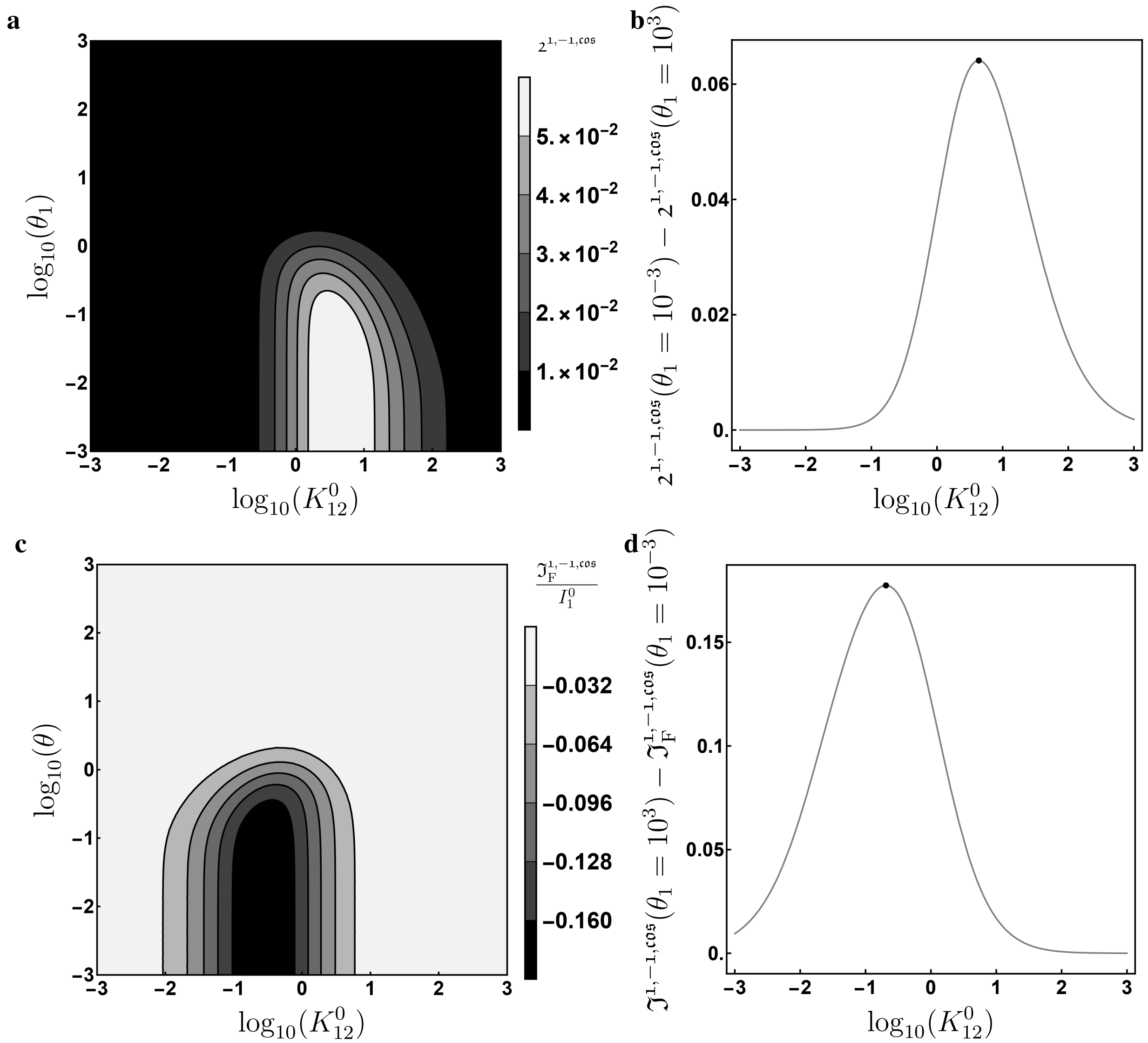

Figure S35: *Dependence of the normalized values of* $\mathfrak{z}^{1,-1,\mathfrak{cos}}$ ***(a)*** *and* $I_F^{1,-1,cos}$ ***(c)*** *on the dimensionless angular frequency* θ *and on the constant* K_{12}^0*, and dependence of* $\mathfrak{z}^{1,-1,\mathfrak{cos}}\left(\theta_1 = 10^{-3}\right) - \mathfrak{z}^{1,-1,\mathfrak{cos}}\left(\theta_1 = 10^{3}\right)$ ***(b)*** *and* $\mathfrak{I}_F^{1,-1,\mathfrak{cos}}\left(\theta_1 = 10^{3}\right) - \mathfrak{I}_F^{1,-1,\mathfrak{cos}}\left(\theta_1 = 10^{-3}\right)$ *on the constant* K_{12}^0 *for a luminophore (*$P_{\text{tot}} = 1\,\text{M}$*, and* $Q_{1,1} = 1$*,* $Q_{1,2} = 0$*,* $Q_{2,1} = 0$*, and* $Q_{2,2} = 0$*) submitted to the reaction (1) upon applying an illumination involving the superposition of two lights at wavelengths* $\boldsymbol{\lambda}_1$ *(sine-wave modulated at angular frequencies* ω_1 *around the averaged value* I_1^0*) and* $\boldsymbol{\lambda}_2$ *(sine-wave modulated at angular frequencies* ω_2 *around the averaged value* I_2^0*) with* $\alpha = 1$ *and* $\delta = 1$*,* $\varphi = \pi$*, and* $\Delta\theta = \theta_2 - \theta_1 = \pi\tau_{12}^0$*.*

C.3 Dimensionalized maps

The effective implementation of HIOM requires to dimensionalize the maps displayed in Figure S35.

C.3.1 Phosphorescent probes

We first considered the case of phosphorescent probes, which can be photoactivated at the wavelength $\boldsymbol{\lambda}_1$ governing the photoconversions from the state 1 to the state 2. Figure S36 displays the dependence of the difference $\mathfrak{z}^{1,-1,\mathfrak{cos}}\left(\omega_1 = 10^2\right) -$

$\mathfrak{z}^{1,-1,\cos}\left(\omega_1 = 10^{10}\right)$ on the light intensity I_1^0 for a phosphorescent probe, which is characterized by $\lambda_1 = 405$ nm, $\sigma_{12,1} = 50$ m^2.mol^{-1}, $\sigma_{21,1} = 0$ m^2.mol^{-1}, and $k_{21}^{\Delta} = 2\ 10^5$ s^{-1}. Figure S36 shows that it is possible to recover a significant amplitude of the phosphorescence signal of the probe over a wide range of light intensity I_1^0. At the optimal I_1^0 value, $\mathfrak{z}^{1,-1,\cos}$ increases by 6% when going from low to high frequency of light modulation.

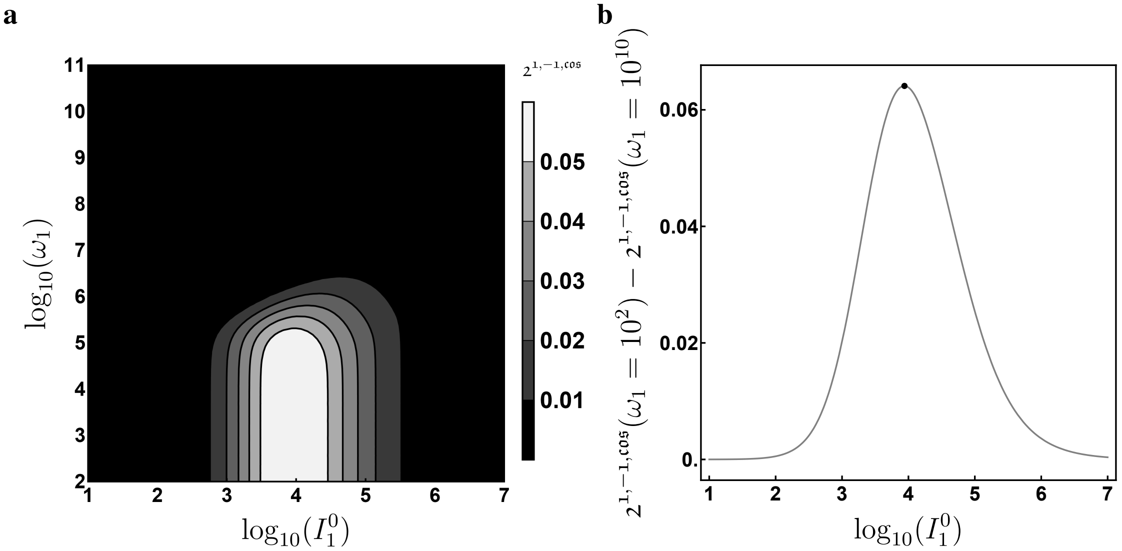

Figure S36: *Dependence of the normalized value of $\mathfrak{z}^{1,-1,\cos}$ on the angular frequency ω_1 and on the light intensity I_1^0 (**a**) and of the normalized difference $\mathfrak{z}^{1,-1,\cos}\left(\omega_1 = 10^2\right) - \mathfrak{z}^{1,-1,\cos}\left(\omega_1 = 10^{10}\right)$ on the light intensity I_1^0 (in Ein.m^{-2}.s^{-1}) (**b**) for a phosphorescent probe ($P_{\rm tot} = 1$ M, $\lambda_1 = 405$ nm, $\sigma_{12,1} = 50$ m^2.mol^{-1}, $\sigma_{21,1} = 0$ m^2.mol^{-1}, and $k_{21}^{\Delta} = 2\ 10^5$ s^{-1}) submitted to the reaction (1) upon applying an illumination involving the superposition of two lights at wavelength λ_1 sinusoidally modulated at angular frequencies ω_1 around the averaged value I_1^0 and ω_2 around the averaged value I_2^0 respectively with $\alpha = 1$, $\varphi = \pi$, $\Delta\omega = \omega_2 - \omega_1 = \pi$.*

In order to retrieve the relaxation time τ_{12}^0, one has to process the dependence of $\mathfrak{z}^{1,-1,\cos}$ on the angular frequency ω_1. To identify a relevant fitting function, we introduced the time dependence of the applied modulated illumination given in Eqs.(79–81) into the master differential equation given in Eq.(21) and retrieved analytic expressions of the time varying terms contained in Eq.(37) upon truncating the Fourier expansion (30) at the 1$^{\rm st}$ order. Then we analyzed the mathematical structure of the resulting function and we decided to adopt the fitting function given in Eq.(98)

$$\mathcal{L}(\omega, p_1, p_2, p_3) = \frac{p1(2 + 3(\omega\tau_{12}^0) + 3(\omega\tau_{12}^0)^2)}{p_2 + 8(\omega\tau_{12}^0) + 12(\omega\tau_{12}^0)^2 + 8(\omega\tau_{12}^0)^3 + 4(\omega\tau_{12}^0)^4} + p_3 \tag{97}$$

in order to extract the relaxation time $\tau_{12}^{0,\rm fit}$ from the computed dependence of the normalized luminescence $\mathfrak{z}^{1,-1,\cos}$ of the phosphorescent probe on the angular frequency ω_1 for various values of the light intensity I_1^0. Figure S37a displays the results. Figure S37b displays the dependence of the fitted and expected relaxation times $\tau_{12}^{0,\rm fit}$ and $\tau_{12}^{0,\rm th}$ on the light intensity I_1^0. It shows that there is a satisfactory agreement over the $[10^{2.5}; 10^{6.5}]$ range of light intensity I_1^0 expressed in Ein.m^{-2}.s^{-1}.

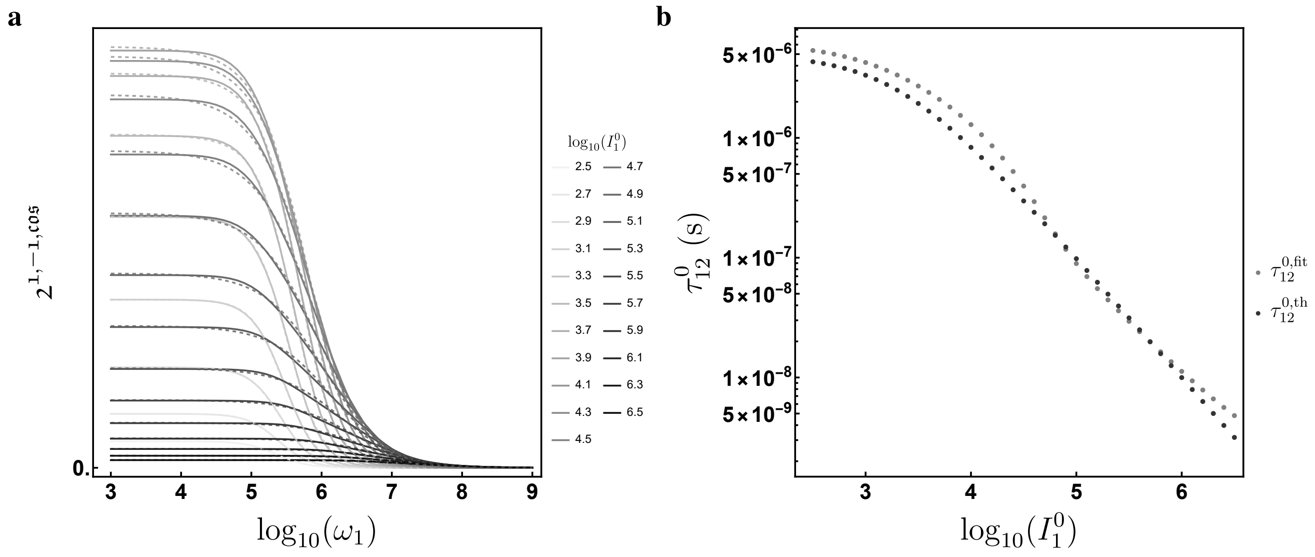

Figure S37: *Retrieval of the relaxation time τ_{12}^0 from processing the dependence of $\mathfrak{I}^{1,-1,\cos}$ on the angular frequency* ω_1. **a**: Dependence of the normalized luminescence $\mathfrak{I}^{1,-1,\cos}$ on the angular frequency ω_1 for various values of the light intensity I_1^0. Solid line: Numerical computation; Dashed line: Fit with Eq.(97); **b**: Dependence of the fitted and expected relaxation times $\tau_{12}^{0,\text{fit}}$ and $\tau_{12}^{0,\text{th}}$ on the light intensity I_1^0 (in Ein.m^{-2}.s^{-1}). Luminophore: Phosphorescent probe ($P_{\text{tot}} = 1$, $Q_{1,1} = 1$, $Q_{1,2} = 0$, $\sigma_{12,1} = 50$ m^2.mol^{-1}, $\sigma_{21,1} = 0$ m^2.mol^{-1}, and $k_{21}^{\Delta} = 2\ 10^5$ s^{-1}) submitted to the reaction (1) upon applying an illumination involving the superposition of two lights at wavelength λ_1 sinusoidally modulated at angular frequencies ω_1 around the averaged value I_1^0 and ω_2 around the averaged value $I_2^0 = I_1^0$ respectively with $\alpha = 1$, $\varphi = \pi$, $\Delta\omega = \omega_2 - \omega_1 = \pi$.

C.3.2 Reversibly photo-convertible fluorophores

We then considered the case of reversibly photo-convertible fluorophores, which can be photoswitched at two wavelengths λ_1 and λ_2 governing the photoconversions from the state 1 to the state 2 and from the state 2 to the state 1 respectively. Figure S38 displays the dependence of the difference $\mathfrak{I}_{\text{F}}^{1,-1,\cos}(\theta_1 = 10^6) - \mathfrak{I}_{\text{F}}^{1,-1,\cos}(\theta_1 = 10^0)$ on the light intensity I_2^0 for the reversibly photo-convertible fluorescent protein Dronpa-2, which is characterized by $\lambda_1 = 488$ nm and $\lambda_2 = 405$ nm, $\sigma_{12,1} = 198$ m^2.mol^{-1}, $\sigma_{21,1} = 0$ m^2.mol^{-1}, $\sigma_{12,2} = 0$ m^2.mol^{-1}, and $\sigma_{21,2} = 415$ m^2.mol^{-1}, and $k_{21}^{\Delta} = 1.4\ 10^{-2}$ s^{-1}.[5] Figure S38 shows that it is possible to recover a significant amplitude of the fluorescence signal of Dronpa-2 over a wide range of light intensity I_2^0. At the optimal I_2^0 value, $\mathfrak{I}_{\text{F}}^{1,-1,\cos}$ increases by 18% when going from low to high frequency of light modulation.

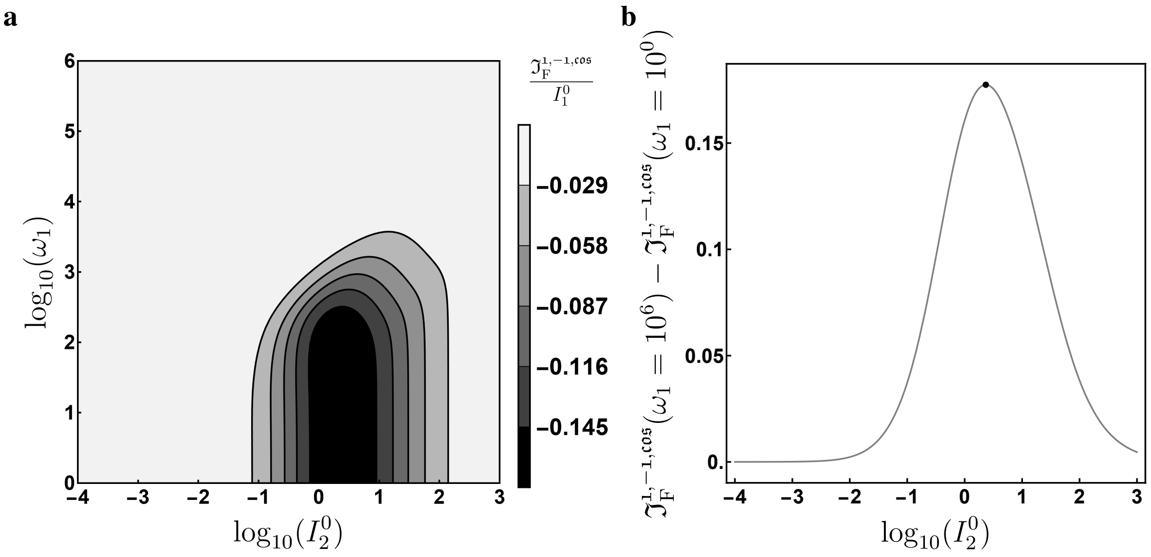

Figure S38: *Dependence of the normalized luminescence $\mathfrak{I}_F^{1,-1,\cos}$ on the angular frequency ω_1 for various values of the light intensity I_2^0 (**a**) and of the normalized difference $\mathfrak{I}_F^{1,-1,\cos}(\theta_1 = 10^6) - \mathfrak{I}_F^{1,-1,\cos}(\theta_1 = 10^0)$ on the light intensity I_2^0 (in Ein.m^{-2}.s^{-1}) (**b**) for Dronpa-2 ($P_{\text{tot}} = 1$ M, $Q_{1,1} = 1$, $Q_{1,2} = 0$, $Q_{2,1} = 0$, $Q_{2,2} = 0$, $\sigma_{12,1} = 198$ m^2.mol^{-1}, $\sigma_{21,1} = 0$ m^2.mol^{-1}, $\sigma_{12,2} = 0$ m^2.mol^{-1}, $\sigma_{21,2} = 415$ m^2.mol^{-1}, and $k_{21}^{\Delta} = 1.4\ 10^{-2}$ s^{-1}) submitted to the reaction (1) upon applying an illumination involving the superposition of two lights at wavelengths λ_1 (sinusoidally modulated at angular frequencies ω_1 around the averaged value I_1^0) and λ_2 (sinusoidally modulated at angular frequencies ω_2 around the averaged value I_2^0) with $\alpha = 1$, $\varphi = \pi$, $\Delta\omega = \omega_2 - \omega_1 = \pi$, and $I_1^0 = 1$ Ein.m^{-2}.s^{-1}.*

In order to retrieve the relaxation time τ_{12}^0, one has to process the dependence of the dependence of $\mathfrak{I}_F^{1,-1,\cos}$ with $\varphi = \pi$ on the angular frequency ω_1. To identify a relevant fitting function, we introduced the time dependence of the applied modulated illumination given in Eqs.(79–81) into the master differential equation given in Eq.(21) and retrieved analytic expressions of the time varying terms contained in Eqs.(49,53) upon truncating the Fourier expansion (30) at the 1st order. Then we analyzed the mathematical structure of the resulting function and we generated the fitting function given in Eq.(98)

$$\mathcal{L}(\omega, p_1, p_2, p_3) = \frac{p1(2 + 3(\omega\tau_{12}^0) + 3(\omega\tau_{12}^0)^2)}{p_2 + 8(\omega\tau_{12}^0) + 12(\omega\tau_{12}^0)^2 + 8(\omega\tau_{12}^0)^3 + 4(\omega\tau_{12}^0)^4} + p_3 \tag{98}$$

in order to extract the relaxation time $\tau_{12}^{0,\text{fit}}$ from the computed dependence of the normalized luminescence $\mathfrak{I}_F^{1,-1,\cos}$ with $\varphi = \pi$ of Dronpa-2 on the angular frequency ω_1 for various values of the light intensity I_2^0. Figure S45a displays the results. Figure S45b displays the dependence of the fitted and expected relaxation times $\tau_{12}^{0,\text{fit}}$ and $\tau_{12}^{0,\text{th}}$ on the light intensity I_2^0. It shows that there is a satisfactory agreement over the $[10^{-2}; 10^2]$ range of light intensity I_2^0 expressed in Ein.m^{-2}.s^{-1}.

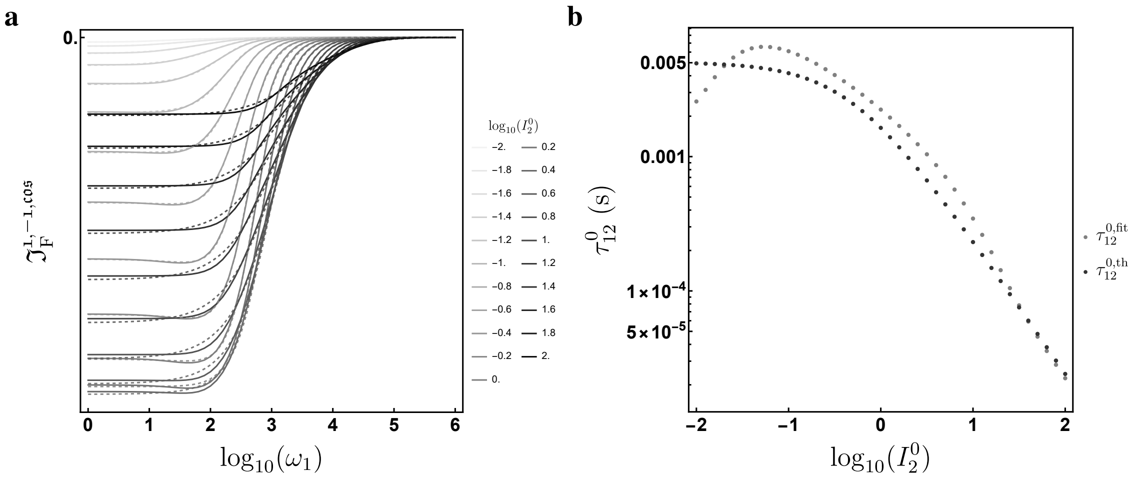

Figure S39: *Retrieval of the relaxation time* τ_{12^0} *from processing the dependence of* $\mathfrak{I}_F^{1,-1,\cos}$ *on the angular frequency* ω_1. **a**: Dependence of $\mathfrak{I}_F^{1,-1,\cos}$ on the angular frequency ω_1 at constant $\Delta\omega = \omega_2 - \omega_1 = \pi$ rad.s^{-1} for various values of the light intensity I_2^0. Solid line: Numerical computation; Dashed line: Fit with Eq.(98); **b**: Dependence of the fitted and expected relaxation times $\tau_{12}^{0,\mathrm{fit}}$ and $\tau_{12}^{0,\mathrm{th}}$ on the light intensity I_2^0 (in Ein.m^{-2}.s^{-1}). Luminophore: Dronpa-2 ($P_{\mathrm{tot}} = 1$ M, $Q_{1,1} = 1$, $Q_{1,2} = 0$, $Q_{2,1} = 0$, $Q_{2,2} = 0$, $\sigma_{12,1} = 198$ m^2.mol^{-1}, $\sigma_{21,1} = 0$ m^2.mol^{-1}, $\sigma_{12,2} = 0$ m^2.mol^{-1}, $\sigma_{21,2} = 415$ m^2.mol^{-1}, and $k_{21}^{\Delta} = 1.4\ 10^{-2}$ s^{-1}) submitted to the reaction (1) upon applying an illumination involving the superposition of two lights at wavelengths λ_1 (sinusoidally modulated at angular frequencies ω_1 around the averaged value I_1^0) and λ_2 (sinusoidally modulated at angular frequencies ω_2 around the averaged value I_2^0) with $\alpha = 1$, $\varphi = \pi$, $\Delta\omega = \omega_2 - \omega_1 = \pi$, and $I_1^0 = 1$ Ein.m^{-2}.s^{-1}.

D Square-wave modulation at two angular frequencies

In relation to the HIOM protocol, we next considered for $I(t)$ an illumination involving the superposition of two lights at wavelengths λ_1 (square-wave modulated at angular frequencies ω_1 around the averaged value I_1^0) and λ_2 (square-wave modulated at angular frequencies ω_2 around the averaged value I_2^0). We adopted the expression, which is given in Eq.(99):

$$I(t) = I_1^0\left[1 + \alpha h_1(t)\right] + I_2^0\left[1 + \alpha\delta h_2(t)\right] \tag{99}$$

$$h_1(t) = \frac{4}{\pi}\sum_{p=0}^{2}\frac{1}{2\times p+1}\sin[(2\times p+1)\theta_1 x] \tag{100}$$

$$h_2(t) = -\frac{4}{\pi}\sum_{p=0}^{2}\frac{1}{2\times p+1}\sin[(2\times p+1)\theta_2 x] \tag{101}$$

D Square-wave modulation at two angular frequencies

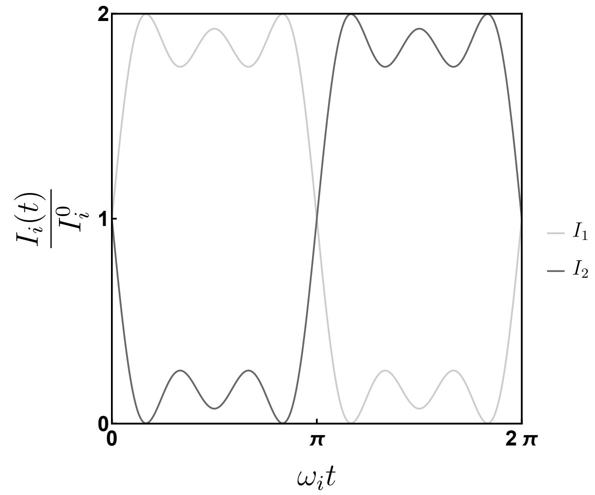

Figure S40: *Antiphase-related square-wave light modulation of large amplitude at angular frequency ω at wavelengths λ_1 (around the averaged value I_1^0) and λ_2 (around the averaged value I_2^0) with $\alpha = 0.84$ and $\delta = 1$.*

D.1 Generic maps

Following the general derivation given in subsection 1.2.1, we analytically retrieved the $2n+1$ unknown terms (a^0,...,a^n,b^n) upon truncating the Fourier expansion (30) at the 5th order. We then established the dependence on angular frequency ω_1 of

- $\mathfrak{z}^{1,-1,\mathfrak{cos}}$ with $\varphi = \pi$ (to address the case of the behavior of the phosphorescent probes in HIOM);
- $\mathfrak{I}_{\mathrm{F}}^{1,-1,\mathfrak{cos}}$ with $\varphi = \pi$ (to address the case of the behavior of the other presently considered fluorescent probes in HIOM)

to selectively discriminate a targeted luminophore and retrieve the relaxation time associated with its reaction (1). Thus, we further analyzed the amplitude of the variation of $\mathfrak{z}^{1,-1,\mathfrak{cos}}$ and $\mathfrak{I}_{\mathrm{F}}^{1,-1,\mathfrak{cos}}$ over the investigated range of dimensionless angular frequency θ_1 on the constant K_{12}^0 at constant $\Delta\theta = \theta_2 - \theta_1 = \pi\tau_{12}^0$. The result is displayed in Figure S41a,b. It makes clear the range of K_{12}^0 values for which a significant amplitude of the luminescence signal of the probe P can be observed: K_{12}^0 should typically range between 10^{-1} and 10^2 whereas θ should be lower than 10^0 for the phosphorescent probes and K_{12}^0 should typically range between 10^{-2} and 10^1 whereas θ should be lower than 10^0 for the fluorescent probes.

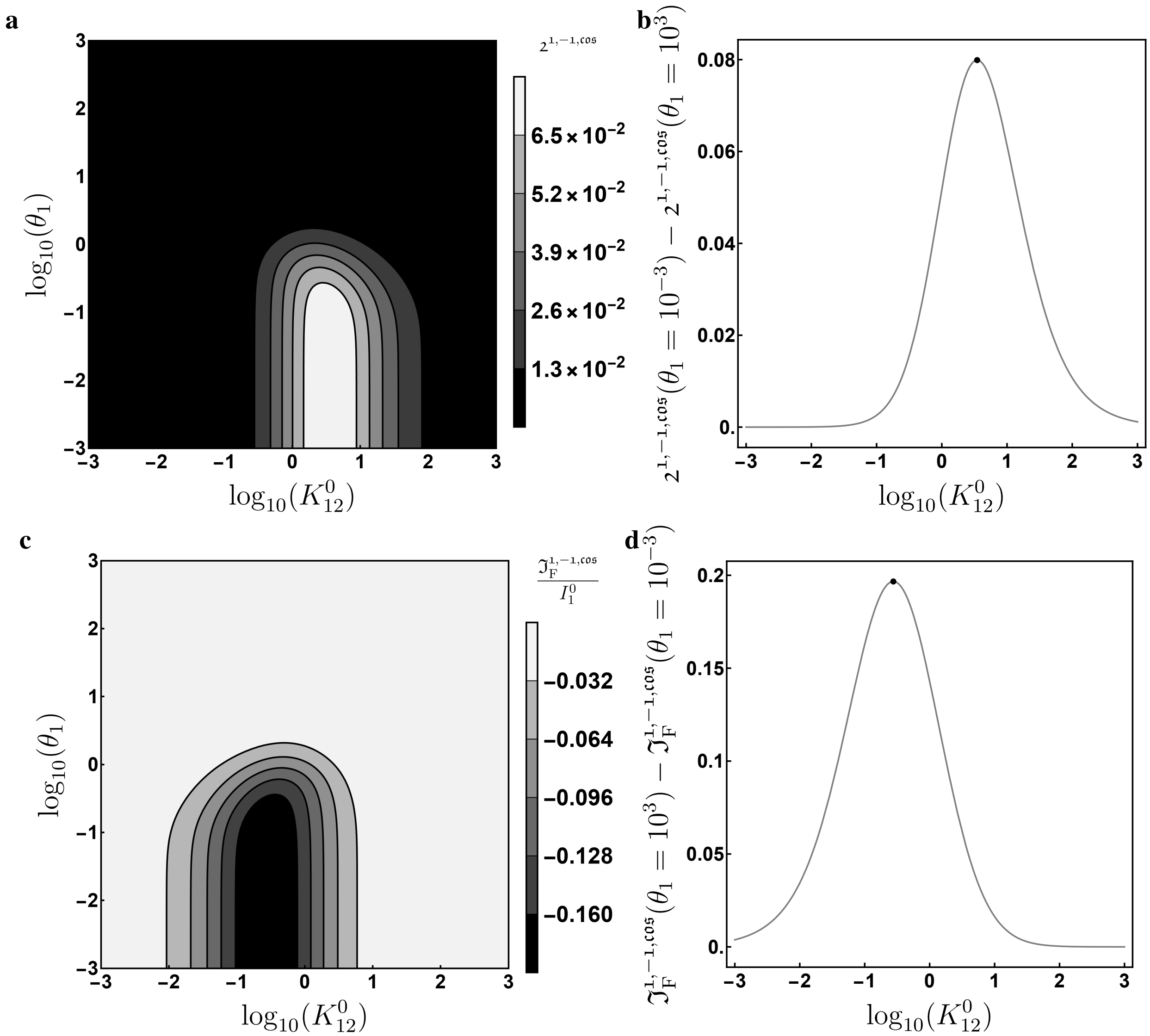

Figure S41: *Dependence of the normalized values of* $\mathfrak{z}^{1,-1,\cos}$ ***(a)*** *and* $I_{\mathrm{F}}^{1,-1,cos}$ ***(c)*** *on the dimensionless angular frequency* θ *and on the constant* K_{12}^0*, and dependence of* $\mathfrak{z}^{1,-1,\cos}\left(\theta_1 = 10^{-3}\right) - \mathfrak{z}^{1,-1,\cos}\left(\theta_1 = 10^{3}\right)$ ***(b)*** *and* $\mathfrak{I}_{\mathrm{F}}^{1,-1,\cos}\left(\theta_1 = 10^{3}\right) - \mathfrak{I}_{\mathrm{F}}^{1,-1,\cos}\left(\theta_1 = 10^{-3}\right)$ *on the constant* K_{12}^0 *for a luminophore (*$P_{\mathrm{tot}} = 1$ M*, and* $Q_{1,1} = 1$*,* $Q_{1,2} = 0$*,* $Q_{2,1} = 0$*, and* $Q_{2,2} = 0$*) submitted to the reaction (1) upon applying an illumination involving the superposition of two lights at wavelengths* λ_1 *(square-wave modulated at angular frequencies* ω_1 *around the averaged value* I_1^0*) and* λ_2 *(square-wave modulated at angular frequencies* ω_2 *around the averaged value* I_2^0*) with* $\alpha = 0.84$ *and* $\delta = 1$*,* $\varphi = \pi$*, and* $\Delta\theta = \theta_2 - \theta_1 = \pi\tau_{12}^0$.

D.2 Dimensionalized maps

D.2.1 Phosphorescent probes

We first considered the case of phosphorescent probes, which can be photoactivated at the wavelength λ_1 governing the photoconversions from the state 1 to the state 2. Figure S42 displays the dependence of the difference $\mathfrak{z}^{1,-1,\cos}\left(\omega_1 = 10^{2}\right) - \mathfrak{z}^{1,-1,\cos}\left(\omega_1 = 10^{10}\right)$ on the light intensity I_1^0 for a phosphorescent probe, which is characterized by $\lambda_1 = 405$ nm,

$\sigma_{12,1} = 50$ m^2.mol^{-1}, $\sigma_{21,1} = 0$ m^2.mol^{-1}, and $k_{21}^{\Delta} = 2\ 10^5$ s^{-1}. Figure S42 shows that it is possible to recover a significant amplitude of the phosphorescence signal of the probe over a wide range of light intensity I_1^0. At the optimal I_1^0 value, $\mathfrak{z}^{1,-1,\mathfrak{cos}}$ increases by 8% when going from low to high frequency of light modulation.

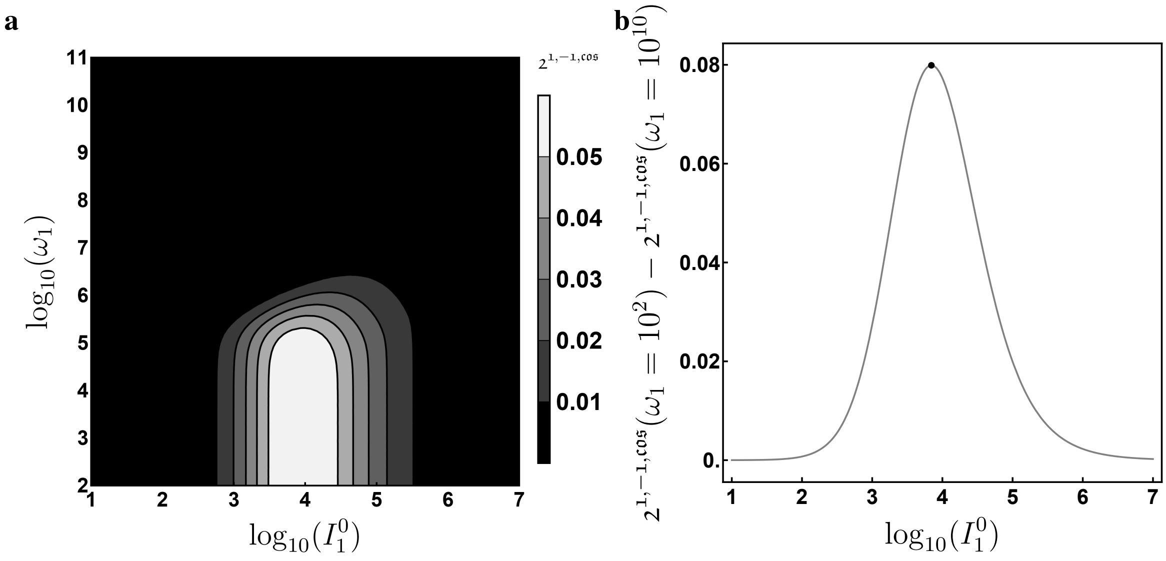

Figure S42: *Dependence of the normalized luminescence* $\mathfrak{z}^{1,-1,\mathfrak{cos}}$ *on the angular frequency* ω_1 *for various values of the light intensity* I_1^0 ***(a)*** *and of the normalized difference* $\mathfrak{z}^{1,-1,\mathfrak{cos}}\left(\omega_1 = 10^2\right) - \mathfrak{z}^{1,-1,\mathfrak{cos}}\left(\omega_1 = 10^{10}\right)$ *on the light intensity* I_1^0 *(in Ein.m*$^{-2}$*.s*$^{-1}$*)* ***(b)*** *for a phosphorescent probe (*$P_{\rm tot} = 1$ M*,* $\boldsymbol{\lambda}_1 = 405$ *nm,* $\sigma_{12,1} = 50$ *m*^{2}*.mol*$^{-1}$*,* $\sigma_{21,1} = 0$ *m*^{2}*.mol*$^{-1}$*, and* $k_{21}^{\Delta} = 2\ 10^5$ *s*$^{-1}$*) submitted to the reaction (1) upon applying an illumination involving the superposition of two lights at wavelengths* $\boldsymbol{\lambda}_1$ *(square-wave modulated at angular frequencies* ω_1 *around the averaged value* I_1^0*) and* $\boldsymbol{\lambda}_2$ *(square-wave modulated at angular frequencies* ω_2 *around the averaged value* I_2^0*) with* $\alpha = 0.84$ *and* $\delta = 1$*,* $\varphi = \pi$*, and* $\Delta\theta = \theta_2 - \theta_1 = \pi\tau_{12}^0$.

In order to retrieve the relaxation time τ_{12}^0, one has to process the dependence of $\mathfrak{z}^{1,-1,\mathfrak{cos}}$ on the angular frequency ω_1. To identify a relevant fitting function, we introduced the time dependence of the applied modulated illumination given in Eqs.(79–81) into the master differential equation given in Eq.(21) and retrieved analytic expressions of the time varying terms contained in Eq.(37) upon truncating the Fourier expansion (30) at the 1$^{\rm st}$ order. Then we analyzed the mathematical structure of the resulting function and we decided to adopt the fitting function given in Eq.(102)

$$\mathcal{L}(\omega, p_1, p_2, p_3) = \frac{p_1(2 + 3(\gamma\omega\tau_{12}^0) + 3(\gamma\omega\tau_{12}^0)^2)}{p_2 + 8(\gamma\omega\tau_{12}^0) + 12(\gamma\omega\tau_{12}^0)^2 + 8(\gamma\omega\tau_{12}^0)^3 + 4(\gamma\omega\tau_{12}^0)^4} + p_3 \tag{102}$$

with $\gamma = 1.85$ in order to extract the relaxation time $\tau_{12}^{0,\rm fit}$ from the computed dependence of the normalized luminescence $\mathfrak{z}^{1,-1,\mathfrak{cos}}$ of the phosphorescent probe on the angular frequency ω_1 for various values of the light intensity I_1^0. Figure S43a displays the results. Figure S43b displays the dependence of the fitted and expected relaxation times $\tau_{12}^{0,\rm fit}$ and $\tau_{12}^{0,\rm th}$ on the light intensity I_1^0. It shows that there is a satisfactory agreement over the $[10^{2.5}; 10^{6.5}]$ range of light intensity I_1^0 expressed in Ein.m^{-2}.s^{-1}.

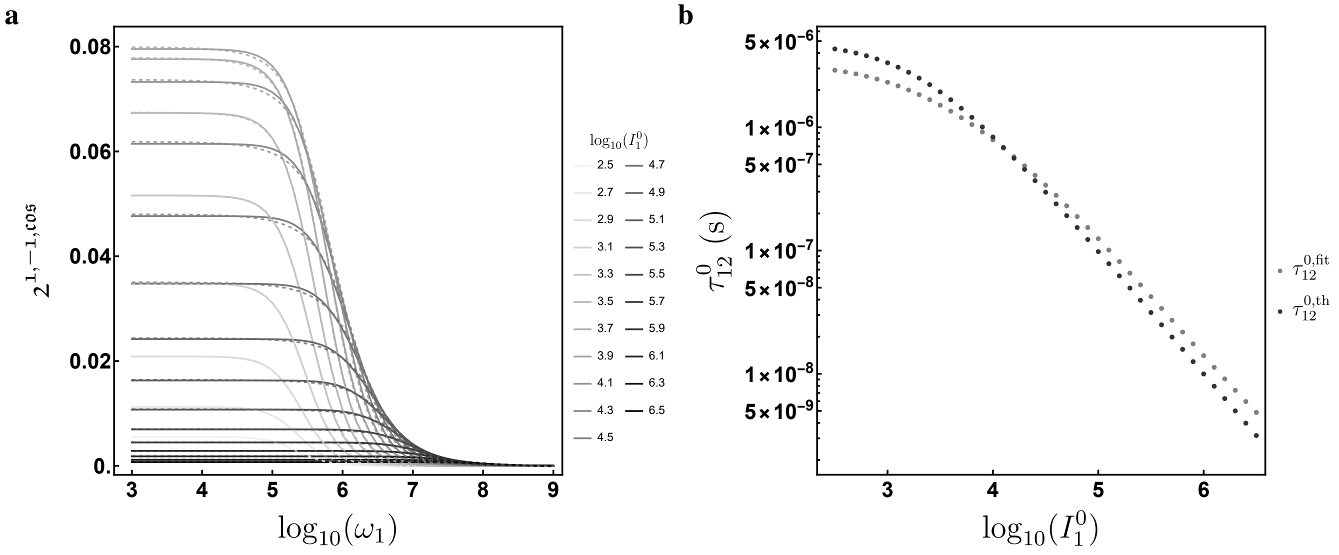

Figure S43: *Retrieval of the relaxation time τ_{12}^0 from processing the dependence of $\mathfrak{z}^{1,-1,\cos}$ on the angular frequency* ω_1. **a**: Dependence of the normalized luminescence $\mathfrak{z}^{1,-1,\cos}$ on the angular frequency ω_1 for various values of the light intensity I_1^0. Solid line: Numerical computation; Dashed line: Fit with Eq.(102); **b**: Dependence of the fitted and expected relaxation times $\tau_{12}^{0,\mathrm{fit}}$ and $\tau_{12}^{0,\mathrm{th}}$ on the light intensity I_1^0 (in Ein.m^{-2}.s^{-1}). Luminophore: Phosphorescent probe ($P_{\mathrm{tot}} = 1$, $Q_{1,1} = 1$, $Q_{1,2} = 0$, $\sigma_{12,1} = 50$ m^2.mol^{-1}, $\sigma_{21,1} = 0$ m^2.mol^{-1}, and $k_{21}^{\Delta} = 2\ 10^5$ s^{-1}) submitted to the reaction (1) upon applying an illumination involving the superposition of two lights at wavelengths λ_1 (square-wave modulated at angular frequencies ω_1 around the averaged value I_1^0) and λ_2 (square-wave modulated at angular frequencies ω_2 around the averaged value I_2^0) with $\alpha = 0.84$ and $\delta = 1$, $\varphi = \pi$, and $\Delta\theta = \theta_2 - \theta_1 = \pi\tau_{12}^0$.

D.2.2 Reversibly photo-convertible fluorophores

We then considered the case of reversibly photo-convertible fluorophores, which can be photoswitched at two wavelengths λ_1 and λ_2 governing the photoconversions from the state 1 to the state 2 and from the state 2 to the state 1 respectively. Figure S44 displays the dependence of the difference $\mathfrak{I}_{\mathrm{F}}^{1,-1,\cos}(\theta_1 = 10^6) - \mathfrak{I}_{\mathrm{F}}^{1,-1,\cos}(\theta_1 = 10^0)$ on the light intensity I_2^0 for the reversibly photo-convertible fluorescent protein Dronpa-2, which is characterized by $\lambda_1 = 488$ nm and $\lambda_2 = 405$ nm, $\sigma_{12,1} = 198$ m^2.mol^{-1}, $\sigma_{21,1} = 0$ m^2.mol^{-1}, $\sigma_{12,2} = 0$ m^2.mol^{-1}, and $\sigma_{21,2} = 415$ m^2.mol^{-1}, and $k_{21}^{\Delta} = 1.4\ 10^{-2}$ s^{-1}.[5] Figure S44 shows that it is possible to recover a significant amplitude of the fluorescence signal of Dronpa-2 over a wide range of light intensity I_2^0. At the optimal I_2^0 value, $\mathfrak{I}_{\mathrm{F}}^{1,-1,\cos}$ increases by 20% when going from low to high frequency of light modulation.

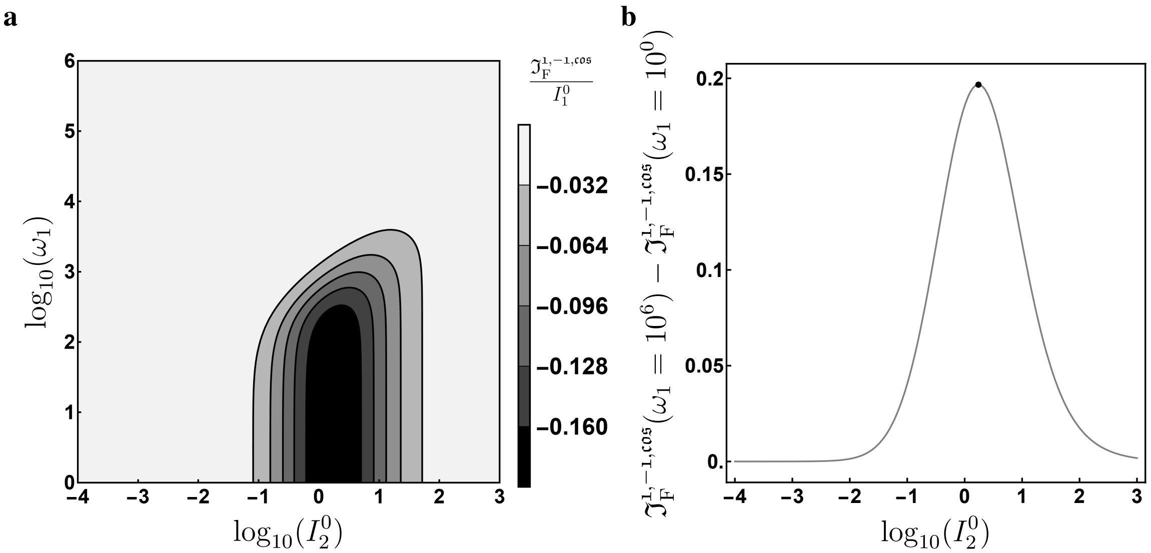

Figure S44: *Dependence of the normalized luminescence* $\mathfrak{I}_F^{1,-1,\cos}$ *on the angular frequency* ω_1 *for various values of the light intensity* I_2^0 ***(a)*** *and of of the normalized difference* $\mathfrak{I}_F^{1,-1,\cos}(\theta_1 = 10^6) - \mathfrak{I}_F^{1,-1,\cos}(\theta_1 = 10^0)$ *on the light intensity* I_2^0 *(in Ein.m*$^{-2}$*.s*$^{-1}$*)* ***(b)*** *for Dronpa-2 (*$P_{\rm tot} = 1$ M, $Q_{1,1} = 1$, $Q_{1,2} = 0$, $Q_{2,1} = 0$, $Q_{2,2} = 0$, $\sigma_{12,1} = 198$ m^2.mol^{-1}, $\sigma_{21,1} = 0$ m^2.mol^{-1}, $\sigma_{12,2} = 0$ m^2.mol^{-1}, $\sigma_{21,2} = 415$ m^2.mol^{-1}, *and* $k_{21}^{\Delta} = 1.4\ 10^{-2}$ s^{-1}*) submitted to the reaction (1) upon applying an illumination involving the superposition of two lights at wavelengths* λ_1 *(square-wave modulated at angular frequencies* ω_1 *around the averaged value* I_1^0*) and* λ_2 *(square-wave modulated at angular frequencies* ω_2 *around the averaged value* I_2^0*) with* $\alpha = 0.84$ *and* $\delta = 1$, $\varphi = \pi$, *and* $\Delta\theta = \theta_2 - \theta_1 = \pi\tau_{12}^0$, *and* $I_1^0 = 1$ *Ein.m*$^{-2}$*.s*$^{-1}$*.*

In order to retrieve the relaxation time τ_{12}^0, one has to process the dependence of the dependence of $\mathfrak{I}_F^{1,-1,\cos}$ with $\varphi = \pi$ on the angular frequency ω_1. To identify a relevant fitting function, we introduced the time dependence of the applied modulated illumination given in Eqs.(79–81) into the master differential equation given in Eq.(21) and retrieved analytic expressions of the time varying terms contained in Eqs.(49,53) upon truncating the Fourier expansion (30) at the 1$^{\rm st}$ order. Then we analyzed the mathematical structure of the resulting function and we generated the fitting function given in Eq.(**??**)

$$\mathcal{L}(\omega, p_1, p_2, p_3) = \frac{p_1(2 + 3(\gamma\omega\tau_{12}^0) + 3(\gamma\omega\tau_{12}^0)^2)}{p_2 + 8(\gamma\omega\tau_{12}^0) + 12(\gamma\omega\tau_{12}^0)^2 + 8(\gamma\omega\tau_{12}^0)^3 + 4(\gamma\omega\tau_{12}^0)^4} + p_3 \quad (103)$$

with $\gamma = 1.85$ in order to extract the relaxation time $\tau_{12}^{0,\rm fit}$ from the computed dependence of the normalized luminescence $\mathfrak{I}_F^{1,-1,\cos}$ with $\varphi = \pi$ of Dronpa-2 on the angular frequency ω_1 for various values of the light intensity I_2^0. Figure S45a displays the results. Figure S45b displays the dependence of the fitted and expected relaxation times $\tau_{12}^{0,\rm fit}$ and $\tau_{12}^{0,\rm th}$ on the light intensity I_2^0. It shows that there is a satisfactory agreement over the $[10^{-2}; 10^2]$ range of light intensity I_2^0 expressed in Ein.m^{-2}.s^{-1}.

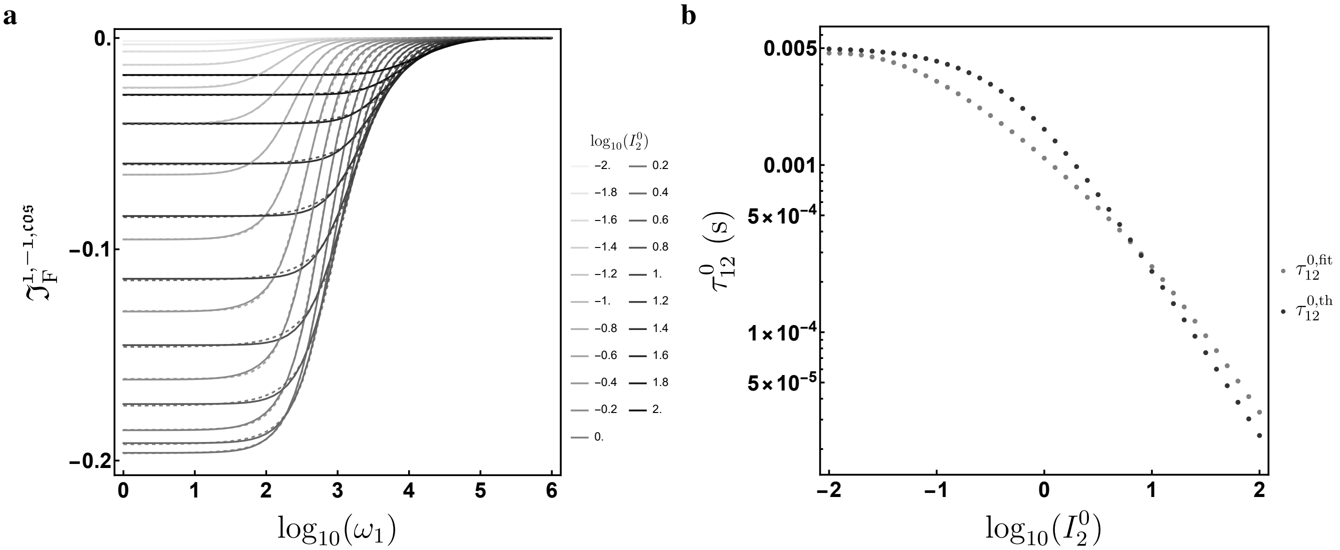

Figure S45: *Retrieval of the relaxation time τ_{12^0} from processing the dependence of $\mathfrak{I}_F^{1,-1,\cos}$ on the angular frequency* ω_1. **a**: Dependence of $\mathfrak{I}_F^{1,-1,\cos}$ on the angular frequency ω_1 at constant $\Delta\omega = \omega_2 - \omega_1 = \pi$ rad.s^{-1} for various values of the light intensity I_2^0. Solid line: Numerical computation; Dashed line: Fit with Eq.(103); **b**: Dependence of the fitted and expected relaxation times $\tau_{12}^{0,\mathrm{fit}}$ and $\tau_{12}^{0,\mathrm{th}}$ on the light intensity I_2^0 (in Ein.m^{-2}.s^{-1}). Luminophore: Dronpa-2 ($P_{\mathrm{tot}} = 1$ M, $Q_{1,1} = 1$, $Q_{1,2} = 0$, $Q_{2,1} = 0$, $Q_{2,2} = 0$, $\sigma_{12,1} = 198$ m^2.mol^{-1}, $\sigma_{21,1} = 0$ m^2.mol^{-1}, $\sigma_{12,2} = 0$ m^2.mol^{-1}, $\sigma_{21,2} = 415$ m^2.mol^{-1}, and $k_{21}^{\Delta} = 1.4\ 10^{-2}$ s^{-1}) submitted to the reaction (1) upon applying an illumination involving the superposition of two lights at wavelengths λ_1 (square-wave modulated at angular frequencies ω_1 around the averaged value I_1^0) and λ_2 (square-wave modulated at angular frequencies ω_2 around the averaged value I_2^0) with $\alpha = 0.84$ and $\delta = 1$, $\varphi = \pi$, and $\Delta\theta = \theta_1 - \theta_2 = \pi$, and $I_1^0 = 1$ Ein.m^{-2}.s^{-1}.

E Reduction of photo(physical)chemical mechanisms to a two-state exchange

In the following subsections, we show that various mechanisms that are relevant of reversibly photoactivatable luminophore can be reduced to the two-state model (1) at an appropriate time scale.

E.1 A two-state electronic model

As a first situation relevant of the fluorescent probes, we analyze the behavior of a chromophore, which yields its first excited singlet state $\mathbf{S}_1$ after light absorption by its ground singlet state $\mathbf{S}_0$. $\mathbf{S}_1$ can subsequently relax to the ground state $\mathbf{S}_0$ (for instance by fluorescence emission) . The overall scheme is displayed in Figure S46.

Relying on the mechanism displayed in Figure S46, we write Eqs.(104–105) to describe the concentration evolutions:

$$\frac{dS_0}{dt} = -k_{01}S_0 + k_{10}S_1 \tag{104}$$

$$\frac{dS_1}{dt} = k_{01}S_0 - k_{10}S_1. \tag{105}$$

Considering that, far from saturation, k_{01} is proportional to the photon flux I, Eq.(105) can be alternatively written

$$\frac{d2}{dt} = -\frac{d1}{dt} = k_{12}^{h\nu}1 - k_{21}^{\Delta}2 \tag{106}$$

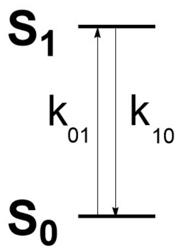

Figure S46: Kinetic model accounting for the behavior of a chromophore yielding its first singlet state upon illumination. The arrows and the associated rate constants refer to the exchange processes. See Text.

with

$$k_{12}^{h\nu} = k_{01} = \sigma I \tag{107}$$

$$k_{21}^{\Delta} = k_{10} \tag{108}$$

in which we identified $\mathbf{S}_0$ and $\mathbf{S}_1$ to 1 and 2 respectively, and introduced the notations $k_{12}^{h\nu}$ to express that the corresponding term is proportional to the incident photon flux with cross section for light absorption σ. Eq.(106) is dynamically identical to Eqs.(2–3) upon noting that $k_{21}^{h\nu} = 0$.

When dealing with a fluorescent probe, the flux of photons associated with fluorescence emission, I_F, is given in Eq.(109)

$$I_F = k_{10}\varphi_F 2 \tag{109}$$

where φ_F designates the quantum yield of fluorescence of the fluorescent probe. At steady state far from saturation, one has

$$2 = \frac{k_{01}}{k_{10}} 1 \simeq \frac{\sigma}{k_{10}} I P_{\mathrm{tot}} \tag{110}$$

since the rate constant k_{10} is typically larger than $10^9 \mathrm{s}^{-1}$[6] and k_{01} is in the 10 s^{-1} range under typical illumination conditions so as to assimilate the concentration 1 to the total concentration of the fluorescent probe P_{tot}.

E.2 A three-state electronic model

As a second situation relevant of the phosphorescent probes, we analyze the behavior of a chromophore, which yields its first excited singlet state $\mathbf{S}_1$ after light excitation of its ground singlet state $\mathbf{S}_0$. $\mathbf{S}_1$ can subsequently relax to the ground state $\mathbf{S}_0$ (for instance by fluorescence emission) or be converted to its first triplet state $\mathbf{T}_1$. $\mathbf{T}_1$ then deexcites to give back the ground state $\mathbf{S}_0$ with emission of phosphorescence. The overall scheme is displayed in Figure S47.

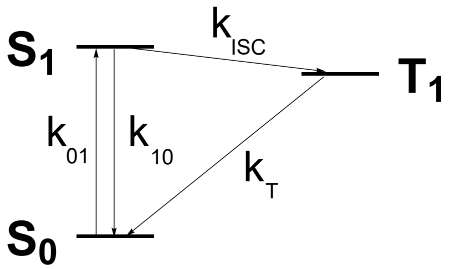

Figure S47: Kinetic model accounting for the behavior of a chromophore yielding its first singlet and triplet excited states upon illumination. The arrows and the associated rate constants refer to the exchange processes. See Text.

Relying on the mechanism displayed in Figure S47, we write Eqs.(111–113) to describe the concentration evolutions:

$$\frac{dS_0}{dt} = -k_{01}S_0 + k_{10}S_1 + k_T T_1 \quad (111)$$

$$\frac{dS_1}{dt} = k_{01}S_0 - (k_{10} + k_{ISC})\,S_1 \quad (112)$$

$$\frac{dT_1}{dt} = k_{ISC}S_1 - k_T T_1. \quad (113)$$

In Figure S47, the rate constant k_{10} is typically larger than $10^9\mathrm{s}^{-1}$.[6] Again $k_{01,i}$ is in the 10 s^{-1} range under typical illumination conditions. In addition k_{ISC} and k_T are usually in the $> 10^6$ and 10^3–10^6 s^{-1} range respectively.[6] Thus we can apply the steady-state approximation to $\mathbf{S}_1$ beyond the nanosecond time scale. Under such conditions, it is meaningful to consider that the states $\mathbf{S}_0$ and $\mathbf{S}_1$ are in a fast exchange so as to introduce an average species **S** with concentration equal to $S_0 + S_1$. Then Eqs.(111–113) reduce to Eq.(114) beyond the time scale at which the steady-state approximation is valid.

$$\frac{dT_1}{dt} = -\frac{dS}{dt} = \frac{k_{ISC}k_{01}}{k_{ISC} + k_{01} + k_{10}}S - k_T T_1. \quad (114)$$

Considering that, far from saturation, k_{01} is proportional to the photon flux I, Eq.(114) can be alternatively written

$$\frac{d2}{dt} = -\frac{d1}{dt} = k_{12}^{h\nu}1 - k_{21}^{\Delta}2 \quad (115)$$

with

$$\frac{k_{ISC}k_{01}}{k_{ISC} + k_{01} + k_{10}} = k_{12}^{h\nu} \quad (116)$$

$$k_{21}^{\Delta} = k_T \quad (117)$$

in which we identified **S** and **T** to 1 and 2 respectively, and introduced the notations $k_{12}^{h\nu}$ to express that the corresponding term is proportional to the incident photon flux. Eq.(115) is dynamically identical to Eqs.(2–3) upon noting that $k_{21}^{h\nu} = 0$.

The preceding mechanistic reduction is relevant to address two distinct situations:

- When dealing with a fluorescent probe in which **S** is the emissive state (e.g. Rhodamine 6G), the flux of photons associated with fluorescence emission, I_F, is given in Eq.(118)

$$I_F = k_{10}\varphi_F S \tag{118}$$

  where φ_F designates the quantum yield of fluorescence of the fluorescent probe;

- When dealing with a phosphorescent probe in which **T** is the emissive state (e.g. metal transition complexes), the flux of photons associated with phosphorescence emission, I_P, is given in Eq.(119)

$$I_P = k_T\varphi_P T \tag{119}$$

  where φ_P designates the quantum yield of phosphorescence of the phosphorescent probe.

E.3 A reversibly photo-convertible luminophore

In a third step, we examine the behavior of a reversibly photo-convertible luminophore, which is relevant of the one of the reversibly photo-convertible fluorescent protein Dronpa-2. Light absorption is supposed to drive the exchange between two ground singlet states denoted $\mathbf{S}_{0,i}$ (i=1 or 2). In the considered scheme, the state $\mathbf{S}_{0,1}$ is supposed to be thermodynamically more stable than the state $\mathbf{S}_{0,2}$. Mechanistically, this exchange involves light absorption leading each ground state $\mathbf{S}_{0,i}$ to its corresponding first singlet excited state $\mathbf{S}_{1,i}$. The latter is then assumed to relax either by leading back to the $\mathbf{S}_{0,i}$ state (for instance by emission of a photon in Dronpa-2) or by photoisomerization (yielding the other ground state). The ground state $\mathbf{S}_{0,2}$ can also notably relax thermally toward the more stable $\mathbf{S}_{0,1}$ state. The overall scheme is displayed in Figure S48.

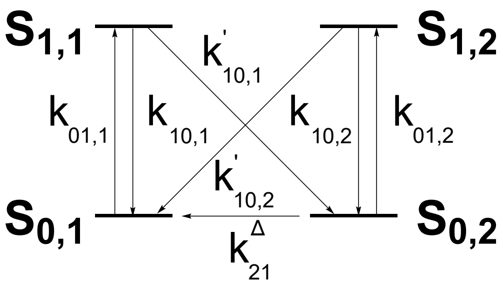

Figure S48: Kinetic model accounting for the behavior of a photo-convertible probe upon illumination. The arrows and the associated rate constants refer to the exchange processes. See Text.

Relying on the mechanism displayed in Figure S48, we write Eqs.(120–123) to describe the concentration evolutions:

$$\frac{dS_{0,1}}{dt} = -k_{01,1}S_{0,1} + k_{10,1}S_{1,1} + k_{21}^{\Delta}S_{0,2} + k_{10,2}^{'}S_{1,2} \tag{120}$$

$$\frac{dS_{0,2}}{dt} = k_{10,1}^{'}S_{1,1} - \left(k_{01,2} + k_{21}^{\Delta}\right)S_{0,2} + k_{10,2}S_{1,2} \tag{121}$$

$$\frac{dS_{1,1}}{dt} = k_{01,1}S_{0,1} - \left(k_{10,1} + k_{10,1}^{'}\right)S_{1,1} \tag{122}$$

$$\frac{dS_{1,2}}{dt} = k_{01,2}S_{0,2} - \left(k_{10,2} + k_{10,2}^{'}\right)S_{1,2}. \tag{123}$$

In Figure S48, the rate constants $k_{10,i}$ and $k_{10,i}^{'}$ are notably much larger than the rate constants $k_{01,i}$ and k_{21}^{Δ}. $k_{10,i}$ and $k_{10,i}^{'}$ are typically larger than 10^9s^{-1}.[6] In contrast, $k_{01,i}$ is in the 10 s^{-1} range under typical illumination conditions.[9] Moreover k_{21}^{Δ} is at most in the 10^6 s^{-1} range. Thus it is possible to apply the steady-state approximation to $\mathbf{S}_{1,1}$ and $\mathbf{S}_{1,2}$ beyond the nanosecond time scale. Under such conditions it is meaningful to consider that the states $\mathbf{S}_{0,i}$ and $\mathbf{S}_{1,i}$ are in a fast exchange so as to introduce an average species **i** for which $\mathbf{S}_{0,i}$ and $\mathbf{S}_{1,i}$ contribute in $p_{\mathbf{S}_{0,i}}$ and $p_{\mathbf{S}_{1,i}}$ respective proportions where

$$p_{\mathbf{S}_{0,i}} = \frac{1}{1+K_{01,i}} \tag{124}$$

$$p_{\mathbf{S}_{1,i}} = \frac{K_{01,i}}{1+K_{01,i}} \tag{125}$$

with

$$K_{01,i} = \frac{k_{01,i}}{k_{10,i} + k_{10,i}^{'}}. \tag{126}$$

Under the experimental conditions used in this study, the relative proportions in the excited and ground states are typically $p_{\mathbf{S}_{1,i}} \sim 10^{-8}$ and $p_{\mathbf{S}_{0,i}} \sim 1$.

Considering that the concentration in **i**, i, is equal to $S_{0,i} + S_{1,i}$, Eqs.(120–123) reduce to Eq.(127) beyond the time scale at which the steady-state approximation is valid.

$$\frac{d2}{dt} = -\frac{d1}{dt} = \left(\frac{K_{01,1}}{1+K_{01,1}}k_{10,1}^{'}\right)1 - \left(\frac{K_{01,2}}{1+K_{01,2}}k_{10,2}^{'} + \frac{1}{1+K_{01,2}}k_{21}^{\Delta}\right)2. \tag{127}$$

Considering that, far from saturation, $K_{01,i}$ is proportional to the photon flux I and much lower than 1, one can write:

$$\frac{K_{01,1}}{1+K_{01,1}}k_{10,1}^{'} \simeq K_{01,1}k_{10,1}^{'} = \frac{k_{10,1}^{'}}{k_{10,1}+k_{10,1}^{'}}\sigma_{01,1}I = k_{12}^{h\nu} \tag{128}$$

$$\frac{K_{01,2}}{1+K_{01,2}}k_{10,2}^{'} \simeq K_{01,2}k_{10,2}^{'} = \frac{k_{10,2}^{'}}{k_{10,2}+k_{10,2}^{'}}\sigma_{01,2}I = k_{21}^{h\nu} \tag{129}$$

$$\frac{1}{1+K_{01,2}}k_{21}^{\Delta} \simeq k_{21}^{\Delta}. \tag{130}$$

[9] Using $k_{01,i} = \sigma_{01,i}I^0$ by considering that the typical photon flux at the sample of an illumination setup is $I^0 = 10^{21}$ photons/m^2s and that the molecular cross section for light absorption $\sigma_{01,i} = 2.3\frac{\epsilon_i}{N_A}$ (N_A is the Avogadro number) is in the 10^{-20} m^2/molecule range for the considered probes.

Hence Eq.(127) can be alternatively written

$$\frac{d2}{dt} = -\frac{d1}{dt} = k_{12}^{h\nu}1 - \left(k_{21}^{h\nu} + k_{21}^{\Delta}\right)2 \tag{131}$$

in which we introduced the notations $k_{12}^{h\nu}$ and $k_{21}^{h\nu}$ to express that the corresponding terms are proportional to the incident photon flux. Eq.(131) is dynamically identical to Eqs.(2–3).

Supplementary References

[1] Aliénor Lahlou, Hessam Sepasi Tehrani, Ian Coghill, Yuriy Shpinov, Mrinal Mandal, Marie-Aude Plamont, Isabelle Aujard, Yuxi Niu, Ladislav Nedbal, Dusan Lazár, et al. Fluorescence to measure light intensity. *Nature Methods*, 20(12):1930–1938, 2023.

[2] Agnès Pellissier-Tanon, Raja Chouket, Ruikang Zhang, Aliénor Lahlou, Agathe Espagne, Annie Lemarchand, Vincent Croquette, Ludovic Jullien, and Thomas Le Saux. Resonances at fundamental and harmonic frequencies for selective imaging of sine-wave illuminated reversibly photoactivatable labels. *ChemPhysChem*, 23(23):e202200295, 2022.

[3] Ruikang Zhang, Raja Chouket, Marie-Aude Plamont, Zsolt Kelemen, Agathe Espagne, Alison G Tebo, Arnaud Gautier, Lionel Gissot, Jean-Denis Faure, Ludovic Jullien, et al. Macroscale fluorescence imaging against autofluorescence under ambient light. *Light: Science & Applications*, 7(1):97, 2018.

[4] J. Quérard, R. Zhang, Z. Kelemen, M.-A. Plamont, X. Xie, R. Chouket, I. Roemgens, Y. Korepina, S. Albright, E. Ipendey, M. Volovitch, H. L. Sladitschek, P. Neveu, L. Gissot, A. Gautier, J.-D. Faure, V. Croquette, T. Le Saux, and L. Jullien. Resonant out-of-phase fluorescence microscopy and remote imaging overcome spectral limitations. *Nat. Comm.*, 8:969, 2017.

[5] R. Chouket, A. Pellissier-Tanon, A. Lahlou, R. Zhang, D. Kim, M.-A. Plamont, M. Zhang, X. Zhang, P. Xu, N. Desprat, D. Bourgeois, A. Espagne, A. Lemarchand, T. Le Saux, and L. Jullien. Extra kinetic dimensions for label discrimination. *Nature communications*, 13(1):1–8, 2022.

[6] Bernard Valeur. *Molecular fluorescence: principles and applications*. Wiley-VCH, 2001.
